# Privacy for free in the overparameterized regime

**DOI:** 10.1073/pnas.2423072122

**Published:** 2025-04-11

**Authors:** Simone Bombari, Marco Mondelli

**Affiliations:** ^a^Institute of Science and Technology Austria, Klosterneuburg 3400, Austria

**Keywords:** differential privacy, deep learning, overparameterization, differentially private gradient descent, random features model

## Abstract

Differentially private gradient descent (DP-GD) is a popular algorithm to train deep learning models with provable guarantees on the privacy of the training data. In the last decade, the problem of understanding its performance cost with respect to standard GD has received remarkable attention from the research community, which has led to upper bounds on the excess population risk $R_{\;\text{P}}$ in different learning settings. However, such bounds typically degrade with overparameterization, i.e., as the number of parameters p gets larger than the number of training samples n—a regime which is ubiquitous in current deep-learning practice. As a result, the lack of theoretical insights leaves practitioners without clear guidance, leading some to reduce the effective number of trainable parameters to improve performance, while others use larger models to achieve better results through scale. In this work, we show that in the popular random features model with quadratic loss, for any sufficiently large p, privacy can be obtained for free, i.e., $\left|R_{\;\text{P}}\right|=o\left(1\right)$, not only when the privacy parameter ε has constant order but also in the strongly private setting ε=o(1). This challenges the common wisdom that overparameterization inherently hinders performance in private learning.

## Significance

In many deep learning applications, training datasets routinely include personal, sensitive information. Learning from these data is possible without creating privacy infringement via methods guaranteeing differential privacy, designed to provide provable protection to any individual user. However, differential privacy comes with a performance cost, and the cost is often believed to grow with the number of parameters of the learning model. Our work challenges this view, showing that overparameterization is not at odds with privacy. In fact, we prove that, for a class of overparameterized models having access to enough training samples, privacy even comes for free, i.e., with a small loss in performance. This result provides theoretical support to the development of differentially private models at scale.

Deep learning models are vulnerable to attacks directed to retrieve information about the training dataset (1, 2), which is concerning when sensitive data are included in the learning pipeline. To allow the usage of such data, differential privacy (DP) (3) consolidated as the golden standard for privacy. This framework comes with algorithms (4) that provide formal protection guarantees for each sample in the training set, which is safeguarded (up to some level) by any adversary with access to the trained model and the rest of the dataset. Specifically, neural networks are trained in a differentially private way via e.g. DP (stochastic) gradient descent (DP-GD) (4). This involves minimizing the training loss with additional “tweaks” to guarantee protection, which typically boil down to i) *clipping* the per-sample gradients before averaging, ii) perturbing the parameters updates with *random noise*, and iii) limiting the number of training iterations with *early stopping*. However, privacy guarantees often come with a performance cost with respect to standard GD (5). Furthermore, private training involves carefully tuning additional hyperparameters, e.g., clipping constant, noise magnitude, and number of training iterations, which increases the computational cost, also due to the higher training times and memory loads of DP optimization (6).

The challenging problem of optimizing neural networks with an assigned privacy guarantee has motivated a thriving field of research proposing architectures and training algorithms (5, 7, 8). Concurrently, theoretical studies have emerged with the scope of quantifying privacy–utility tradeoffs. Privacy is often defined via the pair of parameters (ε,δ): The impact of a single data point on the output of the algorithm is controlled by ε with probability 1−δ; see *Definition* 2.1. To provide meaningful protection, practitioners pick constant-order values of ε (ε∈{1,2,4,8}) and δ<1/n, where n is the number of training samples (9). Utility is typically measured as the degradation in generalization of the DP solution $\theta^{p}\in R^{p}$ compared to a nonprivate baseline $\theta^{*}\in R^{p}$, where p is the number of parameters of the model. Considering the standard supervised setting and denoting by $\left(x,y\right)∼P_{\mathrm{XY}}$ an input–label pair with distribution $P_{\mathrm{XY}}$, the excess population risk is defined as

[1] $$\begin{array}{c} R_{\;\text{P}}=E_{\left(x,y\right)∼P_{\mathrm{XY}}}\left[\ell (x,y,\theta^{p})\right]-E_{\left(x,y\right)∼P_{\mathrm{XY}}}\left[\ell (x,y,\theta^{*})\right], \end{array}$$

where $\ell \left(x,y,\theta \right)$ is the loss over the sample (x,y) of the model evaluated in θ. Intuitively, $R_{\;\text{P}}$ worsens with more stringent privacy requirements on $\theta^{p}$ (i.e., smaller values of ε,δ), and a rich line of work spanning over a decade has investigated the trade-off (9–15). Despite this flurry of research, existing results are unable to address the overparameterized regime, i.e., p=Ω(n), as bounds on $R_{\;\text{P}}$ become vacuous (see the comparison with previous work below). This is sometimes understood via the qualitative argument that the noise introduced by DP-GD increases with the dimension of the parameter space (16, 17), and it has lead to DP algorithms acting on lower dimensional subspaces (7, 8, 18, 19).

**Fig. 1.**
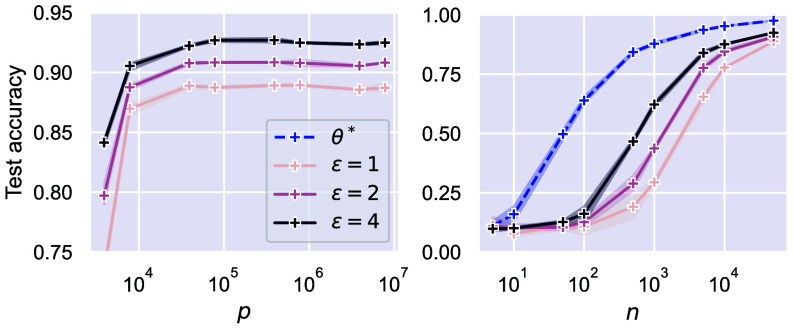
Test accuracy of DP-GD on MNIST for a 2-layer, fully connected ReLU network, plotted as a function of (*Left*) the number of parameters p with fixed n=50,000, and (*Right*) the number of training samples n with fixed hidden layer width =1,000. Further details on the experimental setting can be found in Section 3.

On the other hand, empirical evidence that larger models are beneficial on down-stream tasks requiring private fine-tuning is provided in refs. 20 and 21, which motivated theoretical studies giving refined privacy–utility tradeoffs (22). Perhaps surprisingly, the recent work (6) gives evidence of the benefits of scale even in the absence of public pretraining data, as the generalization performance improves with model size on CIFAR-10 and ImageNet, following an accurate hyperparameter search. In the *Left* panel of Fig. 1, we investigate the interplay between privacy and overparameterization in a simpler and more controllable setting: training a 2-layer, fully connected ReLU network on MNIST with DP-GD (*Algorithm* 1). We vary the network width, spanning both the underparameterized and overparameterized regime, questioning whether the algorithm suffers as the number of parameters grows. The plot shows that this is not the case: The test accuracy increases until the network is wide enough and then plateaus. Furthermore, the gap between the GD solution $\theta^{*}$ and the DP-GD one tends to vanish by increasing the number of training samples n; see the *Right* panel of Fig. 1.

For nonprivate optimization, the apparent contradiction between the excellent generalization of overparameterized models and the classical bias–variance tradeoff has been the subject of intense investigation, highlighting e.g. the role of benign overfitting (23, 24) and double descent (25, 26). The phenomenology discussed above is hard to explain given the current theoretical understanding of overparameterized private training. Thus, this calls for a framework able to i) provide generalization guarantees, and ii) characterize how the hyperparameters of DP-GD affect performance.

### 1.1. Informal Overview.

In this work, we provide privacy–utility guarantees $R_{\;\text{P}}=o\left(1\right)$ under overparameterization—not only when ε has constant order but also in the strongly private setting ε=o(1). We frame this result as achieving privacy for free in the overparameterized regime.

We consider a family of models where the number of parameters p can be significantly larger than the number of samples n and the input dimension d, as in Fig. 1. Specifically, we focus on the widely studied random features (RF) model (27) with quadratic loss, which takes the form

[2] $$\begin{array}{c} \ell \left(x,y,\theta \right)={\left(f_{\text{RF}}\left(x,\theta \right)-y\right)}^{2},\;f_{\text{RF}}\left(x,\theta \right)=\phi {\left(Vx\right)}^{⊤}\theta , \end{array}$$

where $f_{\text{RF}}$ is a generalized linear model, ϕ:R→R a nonlinearity applied component-wise to the vector $Vx\in R^{p}$, and $V\in R^{p\times d}$ a random weight matrix. The RF model can be regarded as a 2-layer network, where only the second layer θ is trained and V is frozen at initialization. Its appeal comes from the fact that it is simple enough to be analytically tractable and, at the same time, rich enough to exhibit properties occurring in more complex deep learning models (23, 26). The DP-trained solution $\theta^{p}$ is obtained via DP-GD (*Algorithm* 1), and the nonprivate baseline $\theta^{*}$ in Eq. **1** via (nonprivate) GD. At this point, we can present an informal version of our result (formally stated in Section 2).

Theorem 1 (informal). *Consider the RF model in Eq. **2** with input dimension* d *and number of parameters* p. *Let* n *be the number of training samples and* $R_{\;\text{P}}$ *be defined according to Eq. **1**, where* $\theta^{*}$ *is the solution of GD and* $\theta^{p}$ *is the* (ε,δ)-*differentially private solution of DP-GD (Algorithm 1). Then, for all sufficiently overparameterized models, under some technical conditions, the following holds with high probability*

[3] $$\begin{array}{c} \begin{array}{c} \left|R_{\;\text{P}}\right|=\tilde{O}\left(\frac{d}{n\varepsilon }+\sqrt{\frac{d}{n}}+\sqrt{\frac{n}{d^{3/2}}}\right)=o\left(1\right). \end{array} \end{array}$$

In words, in the regime $d\ll n\ll d^{3/2}$—considered e.g. in refs. 28 and 29; see Eq. **6** for details—we show that $\left|R_{\;\text{P}}\right|=o\left(1\right)$ as long as ε≫d/n. In fact, when $d\ll n\ll d^{3/2}$ and ε≫d/n, the three terms $\frac{d}{n\varepsilon }$, $\sqrt{\frac{d}{n}}$ and $\sqrt{\frac{n}{d^{3/2}}}$ appearing in the RHS of Eq. **3** become o(1). We make two observations: i) as d≪n, our result guarantees vanishing excess population risk, even with a strong privacy requirement ε=o(1); ii) the bound in Eq. **3** does not depend on p as we only require a lower bound on it (Eq. **6**). The dependence of $R_{\;\text{P}}$ on δ is only logarithmic and it is neglected in the notation $\tilde{O}\left(\cdot \right)$ that hides polylogarithmic factors in δ and n.

### 1.2. Comparison with Previous Work.

In the setting of convex unconstrained optimization, (12) focuses on generalized linear models (GLMs, i.e., ℓ(x,y,θ) is replaced by $\ell (\varphi {\left(x\right)}^{⊤}\theta ,y)$, where φ is the feature map), assumes L-Lipschitz loss with strongly convex regularization, and bounds the excess population risk with respect to the Bayes optimal solution $\theta^{*}$ as $R_{\;\text{P}}=\tilde{O}\left(L\right\|\theta^{*}{\|}_{2}/\sqrt{n}\varepsilon )$. (14) lifts the assumption on the strong convexity and improves the previous bound via the projector M on the column space of $E_{x∼P_{X}}\left[\varphi \left(x\right)\varphi {\left(x\right)}^{⊤}\right]$. (22) considers Lipschitz losses with $\ell_{2}$ regularization and recovers the bound of ref. 14 for GLMs. A similar approach is taken by Ma et al. (30) that removes the dependence on p at the cost of an additional factor $\mathrm{tr}(\tilde{H})$, where $\tilde{H}⪰\;\underset{\theta }{\sup}\nabla_{\theta }^{2}\;E_{\left(x,y\right)∼P_{\mathrm{XY}}}\left[\ell \left(x,y,\theta \right)\right]$. (31) considers GLMs and it also recovers the result of ref. 14 in the Lipschitz setting with ε=Ω(1).

Importantly, even for an RF model, existing bounds do not access the overparameterized regime: Taking ε of constant order makes the upper bounds on the excess population risk to read (at best) $R_{\;\text{P}}=O\left(1\right)$, which is vacuous as the performance of a trivial model outputting zero is of the same order. We now explain why this is the case. First, note that the RF model in Eq. **2** has a non-Lipschitz (quadratic) loss, which does not allow a direct application of most previous bounds. To ensure a fair comparison, one can estimate ${\left\|\theta^{*}\right\|}_{2}$ and evaluate the Lipschitz constant of the training loss restricted to a bounded set B with radius ${\left\|\theta^{*}\right\|}_{2}$. This provides the scaling of an effective Lipschitz constant of the model, as if the optimization was bounded to the set B. From our results in later sections, it can be shown that ${\left\|\theta^{*}\right\|}_{2}=\text{Θ}(\sqrt{n/p})$, which gives ${\left\|\theta^{*}\right\|}_{2}\sup_{\theta \in B}{\left\|\nabla_{\theta }l(x_{i},y_{i},\theta )\right\|}_{2}=\text{Θ}(n)$. This trivializes the bound in ref. 12 to $R_{\;\text{P}}=\tilde{O}\left(\sqrt{n}/\varepsilon \right)$. Furthermore, even by assuming that the loss function in Eq. **2** is Lipschitz, the result improves only by a factor $\sqrt{n}$, i.e., $R_{\;\text{P}}=\tilde{O}\left(1/\varepsilon \right)$, which is again trivial when ε=Θ(1). Similar considerations apply to the bounds in refs. 14, 22, and 31. As concerns the result in ref. 30, it can be verified that $\text{tr}(\tilde{H})\geq E_{x}\left[{\left\|\varphi (x)\right\|}_{2}^{2}\right]=\text{Θ}(p)$, which reintroduces the dependence on the number of parameters p, preventing an improvement upon (12). Finally, while (31) gives bounds for quadratic losses, the reasoning resembles the one above on the effective Lipschitz constant in B and it does not lead to an improvement with respect to the Lipschitz case. The detailed calculation of the quantities mentioned in this paragraph is deferred to *SI Appendix*, section 1.A, together with an additional review of the related literature [e.g., on constrained optimization (9, 13), parameter estimation (32, 33), and linear regression (15, 34)].

Algorithm 1 DP-GD

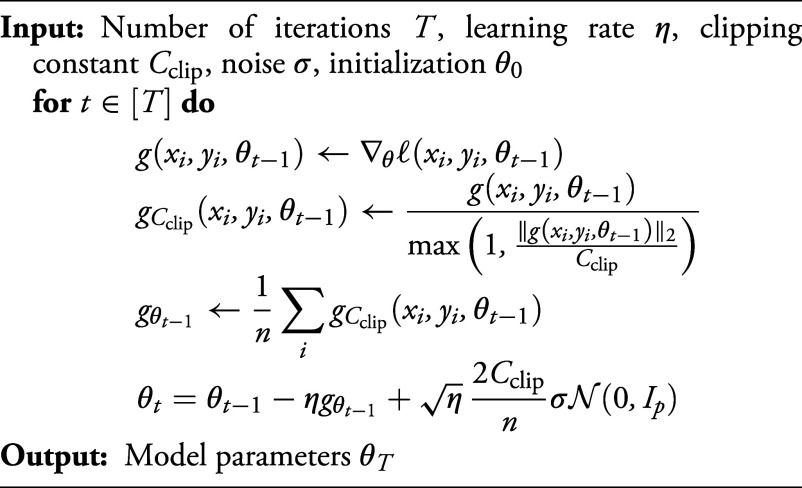

## 2. Setting and Main Result

### 2.1. Differential Privacy (DP) and DP-GD.

The definition of DP builds on the notion of adjacent datasets. In our setting, a dataset $D^{\prime }$ is said to be adjacent to a dataset D if they differ by only one sample.

Definition 2.1 [(*ε,δ*)-DP (3)]. A randomized algorithm $A:D\to R^{p}$ satisfies (ε,δ)-differential privacy if for any pair of adjacent datasets $D,D^{\prime }$∈D and for any $S\subseteq R^{p}$, we have

[4] $$\begin{array}{c} P\left(A(D)\in S\right)\leq e^{\varepsilon }P\left(A(D^{\prime })\in S\right)+\delta . \end{array}$$

Here, the probability is with respect to the randomness induced by the algorithm, and the inequality has to hold uniformly on all the adjacent datasets D and $D^{\prime }$.

A popular choice to enforce (ε,δ)-DP relies on DP-GD algorithms, which perturb individual updates during training, providing privacy guarantees based on the size of the perturbation and the number of iterations (4, 13, 35). In this work, we consider *Algorithm* 1, a variant of the well-established method in ref. 4 without stochastic batching (not considered for simplicity). Its privacy guarantees are below, with the complete argument deferred to *SI Appendix*, section 2.

Proposition 2.2 *For any* δ∈(0,1), ε∈(0,8log(1/δ)), *if we set*

[5] $$\begin{array}{c} \sigma \geq \sqrt{\eta T}\frac{\sqrt{8\log(1/\delta )}}{\varepsilon }, \end{array}$$

*then Algorithm 1 is* (ε,δ)-*differentially private*.

### 2.2. Problem Setup.

We consider *Algorithm* 1 in an overparameterized RF model. For simplicity, we set the initialization $\theta_{0}=0$. The random features matrix $V\in R^{p\times d}$ is i.i.d. Gaussian, i.e., $V_{i,j}{∼}_{i.i.d.}N\left(0,1/d\right)$. We assume the n training samples $\left\{\left(x_{1},y_{1}\right),\ldots ,\left(x_{n},y_{n}\right)\right\}$ to be i.i.d. taken from the joint distribution $P_{\mathrm{XY}}$, such that the labels $\left(y_{1},\ldots ,y_{n}\right)$ are bounded and the marginal $P_{X}$ satisfies the following properties: i) $x∼P_{X}$ is sub-Gaussian, with $∥x∥=O\left(1\right)$; ii) the data $x∼P_{X}$ are normalized, i.e., ${\left\|x\right\|}_{2}=\sqrt{d}$; iii) $\lambda_{\min}\left(E_{x∼P_{X}}\left[xx^{⊤}\right]\right)=\Omega \left(1\right)$, i.e., the second-moment matrix is well conditioned. Taken together, these requirements are implied by the stronger conditions in refs. 26 and 28. Furthermore, they are fulfilled by normalized multivariate Gaussians with well-conditioned covariance and by the normalized features of a class of fully connected neural networks (36). We note that the adjacency of *Definition* 2.1 is not subject to our distributional assumptions and *Proposition* 2.2 holds with D and $D^{\prime }$ differing by a sample in any arbitrary way.

The scaling of input data and random features V guarantees that the preactivations of the model (i.e., the entries of Vx) are of constant order. We then process the entries of Vx via an activation function ϕ:R→R, which we require to be nonlinear, Lipschitz continuous, and such that $\mu_{0}=\mu_{2}=0$, and $\mu_{1}\neq 0$, where $\mu_{k}$ denotes its k-th Hermite coefficient. This choice is motivated by theoretical convenience, and it covers a wide family of activations, including all odd ones (e.g., tanh). We believe that our result can be extended to a more general setting, as the one in ref. 28, at the cost of a more involved analysis.

We further consider the following scaling of the problem

[6] $$\begin{array}{c} n=O\left(\sqrt{p}\right),\;n=\omega \left(d\log^{2}d\right),\;n=o\left(\frac{d^{3/2}}{\log^{3}d}\right). \end{array}$$

To guarantee that the RF model interpolates the data, it suffices that p≫n (see e.g. refs. 28 and 37), and we expect our result to hold under this milder assumption on p as well. We also assume $\log n=\Theta \left(\log p\right)$, which is mild and could be relaxed at the expenses of a polylogarithmic dependence on p in our final result in *Theorem* 2. We finally remark that the regime $d\ll n\ll d^{3/2}$ corresponds to standard datasets, such as CIFAR-10 ($n=5\cdot 10^{4}$, $d\approx 3\cdot 10^{3}$), or ImageNet as considered in ref. 38 ($n\approx 1.3\cdot 10^{6}$, $d\approx 9\cdot 10^{4}$).

According to *Proposition* 2.2, we consider values of the privacy budget δ∈(0,1), ε∈(0,8log(1/δ)), and

[7] $$\begin{array}{c} \frac{\varepsilon }{\sqrt{\log(1/\delta )}}=\omega \left(\frac{d\log^{5}n}{n}\right). \end{array}$$

This lower bound on ε still allows for strong privacy regimes with ε=o(1), as n≫d from Eq. **6**. We set the hyperparameters of *Algorithm* 1 as

[8] $$\begin{array}{c} T=\frac{d\;\log^{2}n}{\eta p},\;C_{\text{clip}}=\sqrt{p}\log^{2}n, \end{array}$$

with σ given by the RHS in *Proposition* 2.2, which guarantees that *Algorithm* 1 is (ε,δ)-DP. We also define the nonprivate baseline as the solution of the gradient flow equation

[9] $$\begin{array}{c} \;\text{d}\hat{\theta }\left(t\right)=-\nabla L\left(\hat{\theta }\left(t\right)\right)\text{d}t,\;\theta^{*}=\;\lim_{t\to +\infty }\hat{\theta }\left(t\right), \end{array}$$

where $L\left(\theta \right)=\frac{1}{n}\sum_{i=1}^{n}\ell \left(\varphi {\left(x_{i}\right)}^{⊤}\theta -y_{i}\right)$ with $\varphi \left(x_{i}\right):=\phi \left(Vx_{i}\right)$ is the training loss (see section 5.1 of ref. 23 for details on the convergence). Then, our main result is formally stated below.

Theorem 2 *Consider the RF model in Eq. **2** with input dimension* d *and number of features* p. *Let* n *be the number of training samples and* $R_{\;\text{P}}$ *be defined in Eq. **1**, where* $\theta^{p}$ *is the solution* $\theta_{T}$ *of Algorithm 1, and* $\theta^{*}$ *is defined in Eq. **9**. Then, as* η *goes to 0, we have that*

$$\begin{array}{c} \left|R_{\;\text{P}}\right|=O\left(\frac{d}{n\varepsilon }\;\log^{5}n\sqrt{\text{log}(1/\delta )}+\sqrt{\frac{d}{n}}+\sqrt{\frac{n\;\log^{3}d}{d^{3/2}}}\right), \end{array}$$

*with probability at least* $1-2\exp\left(-c\log^{2}n\right)$, *where* c *is an absolute constant*.

Existing work studies $R_{\;\text{P}}$ by i) bounding the excess empirical risk of $\theta^{p}$ via convex optimization techniques, and by ii) extending the result to the excess population risk via stability arguments (9, 14, 22). In contrast, we consider the continuous process defined by *Algorithm* 1 as η→0, for the RF model [Proposition 2.7 of *SI Appendix*, section 2 ensures that the limit is (ϵ,δ)-DP]. This allows the use of probabilistic tools that provide a refined control on the trajectory of DP-GD. This approach has proven useful in the non private setting (28, 39), and in this work we apply it to DP learning. We also note that obtaining *Theorem* 2 still required overcoming significant technical barriers: While Mei et al. and Hu et al. (28, 39) establish the asymptotic test error of $\theta^{*}$ at convergence, we need a nonasymptotic control (in terms of n,d,p) on the whole DP-GD trajectory to understand the impact of clipping and early stopping. This in turn leads to a completely different proof strategy, as discussed in Section 4.

## 3. Numerical Results and Discussion

### 3.1. Overparameterization Not at Odds with Privacy.

**Fig. 2.**
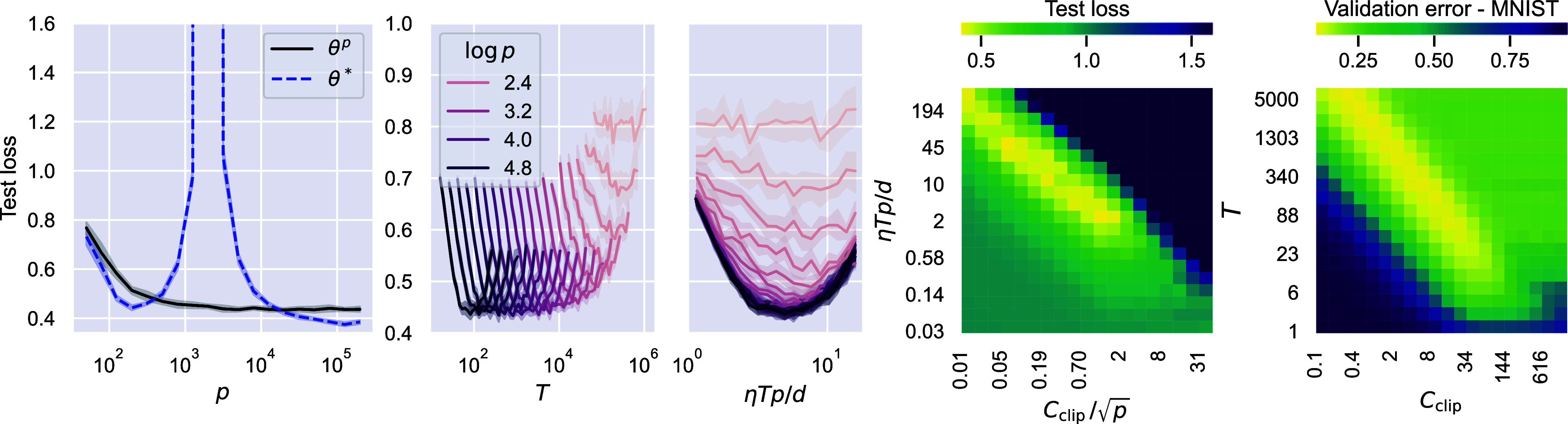
Experiments on RF models with tanh activation and synthetic data sampled from a standard Gaussian distribution with d=100 (first four panels), and on a 2-layer fully connected ReLU network trained with cross-entropy loss on MNIST (d=768, n=50,000) with privacy budget ε=1, δ=1/n (last panel). For the RF model, the learning task is given by $y=\mathrm{sign}(u^{⊤x})$, where $u\in R^{d}$ is a fixed vector sampled from the unit sphere, and we consider a fixed number of training samples n=2,000; $\theta^{p}$ is the solution of *Algorithm* 1 with ε=4, δ=1/n, and $\theta^{*}$ is the solution of GD, both with small enough learning rate η. *First panel*: Test loss of $\theta^{p}$ and $\theta^{*}$ for different number of parameters p. *Second panel*: Test loss of $\theta^{p}$ as a function of the number of training iterations T. *Third panel*: Same plot as in the second panel, with x-axis set to ηTp/d. *Fourth panel*: Test loss of $\theta^{p}$ for a fixed p=40,000, as a function of the hyperparameters $\left(C_{\text{clip}},T\right)$, in dark blue all values of the loss above 1.6. *Fifth panel*: Validation error for a fixed hidden-layer width set to 1,000 ($p∼10^{6}$), as a function of the hyperparameters $\left(C_{\text{clip}},T\right)$.

*Theorem* 2 proves that, in the RF model, overparameterization is not inherently detrimental to private learning. The first panel of Fig. 2 verifies this by plotting the test loss of an RF model trained on a synthetic dataset via DP-GD, as the number of parameters p increases. We also report the performance achieved by (nonprivate) GD, which provides the baseline $\theta^{*}$. While the test loss of $\theta^{*}$ displays the typical double-descent curve (25, 26, 40), with the expected peak at the interpolation threshold (p=n), the performance of $\theta^{p}$ steadily improves and, as p increases, it plateaus to a value close to the loss of $\theta^{*}$. This is in agreement with *Theorem* 2, which predicts a small performance gap between $\theta^{p}$ and $\theta^{*}$ for overparameterized models. Furthermore, the lack of an interpolation peak in the test loss of DP-GD points to the regularization offered by this algorithm and it resembles the effect of a ridge penalty; see ref. 41 for a connection between ridge and early stopping and also the discussion after Lemma 3.4 of *SI Appendix*, section 3.

### 3.2. Role of Hyperparameters.

Both in Fig. 1 and in the first panel of Fig. 2, the hyperparameters in DP-GD are chosen to maximize the validation performance. In the second panel of Fig. 2, we take $C_{\text{clip}}=0.5\sqrt{p}$ and report the test loss as a function of the number of iterations T, setting the noise according to *Proposition* 2.2 to guarantee the desired privacy budget. As suggested by Eq. **8**, the optimal T minimizing the test error decreases with p. More specifically, as we rescale the plot putting ηTp/d on the x-axis of the third panel, the curves collapse onto each other, confirming the accuracy of the proposed scaling. The heat-map in the fourth panel displays the results of a full hyperparameter grid search over $\left(C_{\text{clip}},T\right)$ for a fixed p and ε. We distinguish 4 regions in hyperparameters space: In the 1) *Top-Right*, we have very low utility due to the large noise, in the 2) *Bottom-Left* the test loss is close to 1 as at initialization, since the model does not have the time to learn, in the 3) *Bottom-Right*, we have larger $C_{\text{clip}}$ which could allow for faster convergence. However, as $C_{\text{clip}}$ becomes larger than the typical per-sample gradient size ($C_{\text{clip}}\gg \sqrt{p}$), the overly pessimistic injection of noise ultimately undermines utility. At the center of the panel, we have $C_{\text{clip}}∼\sqrt{p}$ and ηT∼d/p as in Eq. **8**, which lead to low test loss. Moving toward the 4) *Top-Left* there is no decrease in performance, which is in line with earlier empirical work (6, 20) noting that wide ranges of $C_{\text{clip}}$ result in optimal performance. A similar picture emerges from the training of 2-layer neural networks with DP-GD and cross-entropy loss on MNIST, as shown in the rightmost panel. The implementation of the experiments is publicly available at the GitHub repository https://github.com/simone-bombari/privacy-for-free.

### 3.3. Privacy for Free.

*Theorem* 2 proves that we can achieve privacy for free, which may seem surprising. The intuition is that in the RF model, when n≫d, there is a surplus of samples that can be used to learn privately. In fact, the test error of (nonprivate) GD plateaus when n is between d and $d^{3/2}$ (28, 39), hence Θ(d) samples are enough to achieve utility, with the remaining ones leading to privacy. The *Right* panel of Fig. 1 displays the phenomenon. On the *Left*, the performance of $\theta^{*}$ increases with n, while private algorithms have low utility. Moving toward the right, the performance of GD saturates, and the utility of DP-GD increases, approaching the nonprivate baseline. The plateau in the test loss of GD has been shown for kernel ridge regression (28, 39, 42) and the RF model exhibits it when $d^{l}\ll n\ll d^{l+1}$ for any l∈N, as long as p≫n; see figure 1 of ref. 39. We expect that DP-GD catches up with the performance of GD in any of these plateaus. However, as n approaches $d^{l+1}$, the test loss of GD sharply decreases and it is unclear whether DP-GD has the same rate of improvement. This suggests that our result could be extended to the regime $d^{l}\ll n\ll d^{l+1}$ with p≫n. In the present paper, we focus on $d\ll n\ll d^{3/2}$ and $p=\Omega (n^{2})$ due to the additional challenges in the analysis of clipping; see the discussion after Lemma 3.5 in *SI Appendix*, section 3.

## 4. Methods

### 4.1. A Continuous View on DP-GD.

Two challenges arise in the analysis of DP-GD as η→0: i) gradient clipping, and ii) noise injection. To overcome the former, we define a clipped loss $L_{C_{\text{clip}}}\left(\theta \right)$. As for the latter, we consider the stochastic differential equation (SDE) obtained by adding a Wiener process to the gradient flow. This motivates the scaling $\sqrt{\eta }$ of the SD of the noise, as done e.g. in ref. 43.

As in ref. 14, we note that clipping in *Algorithm* 1 can be reformulated as optimizing the surrogate loss $\ell_{i,C_{\text{clip}}}\left(\varphi {\left(x_{i}\right)}^{⊤}\theta -y_{i}\right)$, whose derivative for the i-th training sample reads

[10] $$\begin{array}{c} \ell_{i,C_{\text{clip}}}^{\prime }\left(z\right)=\ell^{\prime }\left(z\right)\min\left(1,\frac{C_{\text{clip}}}{\left|\ell^{\prime }\left(z\right)\right|{\varphi (x_{i})}_{2}}\right). \end{array}$$

In other words, running *Algorithm* 1 is equivalent to running the same algorithm, without the clipping step, on the clipped loss $L_{C_{\text{clip}}}\left(\theta \right)=\frac{1}{n}\sum_{i=1}^{n}\ell_{i,C_{\text{clip}}}\left(\varphi {\left(x_{i}\right)}^{⊤}\theta -y_{i}\right)$. Hence, we can write the t-th iteration of *Algorithm* 1 as

[11] $$\begin{array}{c} \theta_{t}-\theta_{t-1}=-\eta \nabla L_{C_{\text{clip}}}\left(\theta_{t-1}\right)+\sqrt{\eta }\frac{2C_{\text{clip}}}{n}\sigma N\left(0,I_{p}\right). \end{array}$$

This update rule corresponds to the Euler–Maruyama discretization scheme of the SDE

[12] $$\begin{array}{c} \;\text{d}\Theta \left(t\right)=-\nabla L_{C_{\;\text{clip}}}\left(\Theta \left(t\right)\right)\;\text{d}t+\Sigma \;\;\text{d}B\left(t\right), \end{array}$$

with discretization η (see section 10.2 of ref. 44). Here, B(t) is a p-dimensional Wiener process, $\Sigma :=2C_{\;\text{clip}}\sigma /n$, and the initial condition of Eq. **12** corresponds to the initialization of *Algorithm* 1, i.e., $\Theta \left(0\right)=\theta_{0}$. The strong convergence of the Euler–Maruyama method guarantees that, for any τ=ηT, $\theta_{T}$ from *Algorithm* 1 approaches Θ(τ) from Eq. **12** as η gets smaller. We note that previous work (45) has considered a similar SDE to analyze the effects of stochastic batching, separating the dynamics in a gradient flow plus a Wiener process. Thus, the approach developed here could prove useful also to study DP-SGD, after incorporating an additional independent Wiener process in Eq. **12**.

Going back to DP-GD, to circumvent the difficulty in explicitly solving Eq. **12**, we consider

[13] $$\begin{array}{c} \;\text{d}\hat{\Theta }\left(t\right)=-\nabla L\left(\hat{\Theta }\left(t\right)\right)\text{d}t+\Sigma \;\;\text{d}B\left(t\right), \end{array}$$

where L(θ) is the original (quadratic) training loss and B(t) is the same Wiener process as in Eq. **12**. The solution of the SDE in Eq. **13** is a multidimensional Ornstein–Uhlenbeck (OU) process which admits a closed form (see, e.g., section 4.4.4 in ref. 46). Let us then define

[14] $$\begin{array}{c} C:=\left\{\theta \;\;\text{s.t.}\;\|\nabla_{\theta }\ell \left(\varphi {\left(x_{i}\right)}^{⊤}\theta -y_{i}\right){\|}_{2}<C_{\text{clip}},\;\forall i\in \left[n\right]\right\}, \end{array}$$

where [n]={1,…,n}. The set C contains the parameters such that clipping does not happen, i.e., $L_{C_{\text{clip}}}\left(\theta \right)=L\left(\theta \right)$. If the entire path of Θ(t) happens in this region (i.e. Θ(t)∈C for all t∈[0,τ]), then $\Theta \left(\tau \right)=\hat{\Theta }\left(\tau \right)$. This is equivalent to

[15] $$\begin{array}{c} \hat{\Theta }\left(t\right)\in C,\;\text{for all}\;t\in \left[0,\tau \right], \end{array}$$

which is an easier event to control, as $\hat{\Theta }\left(t\right)$ is an OU process.

### 4.2. Analysis of Clipping.

We show that, by choosing the hyperparameters as in Eq. **8** and setting τ=Tη, the event in Eq. **15** happens with high probability. To do so, we decompose $\hat{\Theta }\left(t\right)=E_{B}\left[\hat{\Theta }\left(t\right)\right]+\tilde{\Theta }\left(t\right)=\hat{\theta }\left(t\right)+\tilde{\Theta }\left(t\right)$, where $\tilde{\Theta }\left(t\right):=\hat{\Theta }\left(t\right)-E_{B}\left[\hat{\Theta }\left(t\right)\right]$ and we use that the expectation of an OU process corresponds to the gradient flow $E_{B}\left[\hat{\Theta }\left(t\right)\right]=\hat{\theta }\left(t\right)$ of Eq. **9**. Then, the probability of the event in Eq. **15** is lower bounded by the probability that

[16] $$\begin{array}{c} \left|\varphi {\left(x_{i}\right)}^{⊤}\tilde{\Theta }\left(t\right)\right|+\left|\varphi {\left(x_{i}\right)}^{⊤}\hat{\theta }\left(t\right)-y_{i}\right|\leq \frac{C_{\text{clip}}}{2{\left\|\varphi (x_{i})\right\|}_{2}}, \end{array}$$

for all i∈[n] and t∈[0,τ]. As $C_{\;\text{clip}}=\sqrt{p}\log^{2}n$ (Eq. **8**) and ${\left\|\varphi (x_{i})\right\|}_{2}=\text{Θ}(\sqrt{p})$ with high probability (*SI Appendix*, Eqs. **296** and **297**), Eq. **16** follows from

[17] $$\begin{array}{c} |\varphi {\left(x_{i}\right)}^{⊤}\hat{\theta }\left(t\right)-y_{i}|=o\left(\log^{2}n\right),\;\left|\varphi {\left(x_{i}\right)}^{⊤}\tilde{\Theta }\left(t\right)\right|=o\left(\log^{2}n\right). \end{array}$$

To obtain the first inequality in Eq. **17**, we show in Lemma 3.1 of *SI Appendix*, section 3, that, jointly for all i∈[n],

[18] $$\begin{array}{c} \underset{t\in [0,\tau ]}{\sup}\left|\varphi {\left(x_{i}\right)}^{⊤}\hat{\theta }\left(t\right)-y_{i}\right|=O\left(\log n\right), \end{array}$$

with high probability. First, we study the stability of GD by proving that ${\left\|\theta^{*}-\theta_{-i}^{*}\right\|}_{2}=\tilde{O}\left(p^{-1/2}\right)$, where $\theta_{-i}^{*}$ is obtained after removing the i-th sample from the training set. Then, we control the entire trajectory of gradient flow for t∈[0,τ] via i) an explicit computation based on Lie’s product formula for the matrix exponential, and ii) a concentration bound over $x_{i}$ based on Dudley’s (chaining tail) inequality.

To obtain the second inequality in Eq. **17**, we show in Lemma 3.2 of *SI Appendix*, section 3 that, jointly for all i∈[n],

[19] $$\begin{array}{c} \underset{t\in [0,\tau ]}{\sup}\left|\varphi {\left(x_{i}\right)}^{⊤}\tilde{\Theta }\left(t\right)\right|=O\left(\log n\right), \end{array}$$

with high probability. We start by noticing that $\varphi {\left(x_{i}\right)}^{⊤}\tilde{\Theta }\left(t\right)$ evolves as a Gaussian random variable with time-dependent variance. The idea is to upper bound this variance with that of the auxiliary process $\;\text{d}z_{i}\left(t\right)=\varphi {\left(x_{i}\right)}^{⊤}\Sigma \;\;\text{d}B\left(t\right)$, which removes the attractive drift $-\nabla L(\hat{\Theta }\left(t\right))$ from Eq. **13**. Then, Sudakov-Fernique inequality gives that $E_{B}\left[\underset{t\in [0,\tau ]}{\sup}\left|\varphi {\left(x_{i}\right)}^{⊤}\tilde{\Theta }\left(t\right)\right|\right]\leq E_{z_{i}}\left[\underset{t\in [0,\tau ]}{\sup}\left|z_{i}\left(t\right)\right|\right]$. Since $z_{i}\left(t\right)$ is a Wiener process, the RHS of the previous equation is $O\left({\text{Σ}}^{2}\tau {\left\|\varphi (x_{i})\right\|}_{2}^{2}\right)$ via the reflection principle, and this upper bound is of constant order by Eqs. **7** and **8**. An application of the Borell-TIS inequality concludes the argument by giving that, with high probability, $\underset{t\in [0,\tau ]}{\sup}|\varphi {\left(x_{i}\right)}^{⊤}\tilde{\Theta }\left(t\right)|\leq E_{B}[\underset{t\in [0,\tau ]}{\sup}|\varphi {\left(x_{i}\right)}^{⊤}\tilde{\Theta }\left(t\right)\left|\right]+\log n$.

### 4.3. Analysis of Noise and Early Stopping.

As $\Theta \left(\tau \right)=\hat{\Theta }\left(\tau \right)$ with high probability (Eq. **15**), we study the utility of Θ(τ) via the closed form of the OU process $\hat{\Theta }\left(\tau \right)$. This boils down to controlling the effects of noise and early stopping, which are decoupled by the decomposition $\hat{\Theta }\left(\tau \right)=\hat{\theta }\left(\tau \right)+\tilde{\Theta }\left(\tau \right)$. In fact, $\tilde{\Theta }\left(\tau \right)$ is a mean-0 random variable (in the probability space of B) that captures the noise and $\hat{\theta }\left(\tau \right)$ is the deterministic component (with respect to B) that captures the early stopping. As $\varphi {\left(x_{i}\right)}^{⊤}\tilde{\Theta }\left(\tau \right)$ is Gaussian with variance increasing linearly in ${\left\|\varphi (x_{i})\right\|}_{2}^{2}$, τ, and $\Sigma^{2}$, we have that (see Lemma 3.3 of *SI Appendix*, section 3 for details)

[20] $$\begin{array}{c} E_{x∼P_{X}}\left[{\left(\varphi {\left(x\right)}^{⊤}\tilde{\Theta }\left(\tau \right)\right)}^{2}\right]=\tilde{O}\left(\frac{d^{2}}{\varepsilon^{2}n^{2}}\right), \end{array}$$

which implies that noise does not damage utility. It remains to show that early stopping is not detrimental. This is proved in Lemma 3.4 of *SI Appendix*, section 3, which informally states that

[21] $$\begin{array}{c} E_{x∼P_{X}}\left[{\left(\varphi {\left(x\right)}^{⊤}\left(\hat{\theta }\left(\tau \right)-\theta^{*}\right)\right)}^{2}\right]=\tilde{O}\left(\frac{d}{n}+\frac{n}{d^{3/2}}\right). \end{array}$$

The idea of the argument is to decompose the LHS of Eq. **21** in two orthogonal subspaces, i.e., $\varphi {\left(x\right)}^{⊤}P_{\Lambda }\left(\hat{\theta }\left(\tau \right)-\theta^{*}\right)$ and $\varphi {\left(x\right)}^{⊤}P_{\Lambda }^{⊥}\left(\hat{\theta }\left(\tau \right)-\theta^{*}\right)$. Here, $P_{\Lambda }\in R^{p\times p}$ is the projector on the space spanned by the top d eigenvectors of $\Phi^{⊤}\Phi$ and $\Phi \in R^{n\times p}$ is the feature matrix containing $\varphi (x_{i})$ in its i-th row. The rationale is that there is a spectral gap between the d-th and the (d+1)-th eigenvalue of the kernel $K=\Phi \Phi^{⊤}$, as proved in Lemma 4.5 of *SI Appendix*, section 4. We note that this result also uses the well conditioning of the data covariance ($\lambda_{\min}\left(E_{x∼P_{X}}\left[xx^{⊤}\right]\right)=\Omega \left(1\right)$); see the discussion right after the proof of Lemma 3.4 in *SI Appendix*, section 3. As a consequence of the spectral gap, $\|P_{\Lambda }\left(\hat{\theta }\left(\tau \right)-\theta^{*}\right){\|}_{2}$ is negligible, since in this subspace $\hat{\theta }\left(\tau \right)$ is already close to convergence, despite the early stopping. To control the other subspace, we resort to the bounds in Lemmas 4.14 and 4.15 of *SI Appendix*, section 4.

To conclude, denoting by $\hat{R}$ and $R^{*}$ the generalization error of $\hat{\Theta }\left(\tau \right)$ and $\theta^{*}$ respectively, Eqs. **20** and **21** guarantee that $|\hat{R}-R^{*}|=\tilde{O}\left(\frac{d}{n\varepsilon }+\sqrt{\frac{d}{n}}+\sqrt{\frac{n}{d^{3/2}}}\right)$. As $\Theta \left(\tau \right)=\hat{\Theta }\left(\tau \right)$ (due to Eq. **15**), the result of *Theorem* 2 follows.

## Supplementary Material

Appendix 01 (PDF)

## Data Availability

All codes for generating the figures have been deposited in GitHub (https://github.com/simone-bombari/privacy-for-free) (47).
