# Supplementary material for: Privacy for free in the overparameterized regime
Source: Proc Natl Acad Sci U S A. 2025 Apr 11;122(15):e2423072122. doi: 10.1073/pnas.2423072122 (PMC12012534; doi:10.1073/pnas.2423072122)
Supplement: Supplementary file 1 — Appendix 01 (PDF) [file pnas.2423072122.sapp.pdf]

Supporting Information for

Privacy for Free in the Over-Parameterized Regime

Simone Bombari, Marco Mondelli

Simone Bombari, Marco Mondelli
E-mail: simone.bombari@ista.ac.at, marco.mondelli@ista.ac.at

This PDF file includes:

Supporting text
SI References

Supporting Information Text

1. Further Discussion on Related Work

Differentially private empirical risk minimization is the problem of minimizing the empirical risk $\mathcal{L}(\theta)=\sum_{i=1}^n \ell(x_i,y_i,\theta)/n$ while also ensuring privacy guarantees, where (x_i,y_i) represents an input-label pair in the training set and ℓ is a loss function. Different algorithms have been proposed to tackle this problem, and the performance is measured through the excess risks of their solution θ^p with respect to the baseline θ^*. In particular, we can define the *excess empirical risk* as the difference in the training losses $\mathcal{R}_\mathrm{E}=\mathcal{L}(\theta^p)-\mathcal{L}(\theta^*)$, and the *excess population risk* $\mathcal{R}_\mathrm{P}$ as the difference in the test losses, see Eq. (1). These quantities capture the privacy-utility trade-off of the DP algorithm, as excess risks worsen with an increase in the privacy guarantees (*i.e.*, with smaller values of the privacy parameter ε). The seminal works (1, 2) quantitatively investigate this trade-off for strongly convex losses in the context of ε-DP (obtained by setting $\delta=0$), both for output and objective perturbation methods.

In the setting of *constrained optimization*, (3) extends the analysis of the objective perturbation in (2), providing a bound on the empirical excess risk of $\mathcal{R}_\mathrm{E}=\tilde{\mathcal{O}}(\sqrt{p}/(n\varepsilon))$ for constrained, strongly convex (ε,δ)-DP optimization (see their Theorem 4). The subsequent work (4) focuses on a variant of DP-GD previously studied in (5), and it bounds the excess empirical risk as $\mathcal{R}_\mathrm{E}=\beta L\,\tilde{\mathcal{O}}(\sqrt{p}/(n\varepsilon))$ (see their Theorem 2.4.2).* Here, β is the diameter of the (bounded) optimization domain $\mathcal{B}$, L is the Lipschitz constant of the loss with respect to the parameters of the model, and the baseline is defined as the training loss minimizer $\theta^*=\arg\min_{\theta\in\mathcal{B}}\mathcal{L}(\theta)$. We note that, in this setting, clipping is not necessary as the loss is Lipschitz. By relying on the algorithmic stability analysis in (6), (4) also provides an excess population risk bound of the form $\mathcal{R}_\mathrm{P}=\tilde{\mathcal{O}}(p^{1/4}/\sqrt{n\varepsilon})$ (see their Theorem F.2.2), where the baseline is the Bayes optimal solution $\theta^*=\arg\min_{\theta\in\mathcal{B}}\mathbb{E}_{(x,y)\sim\mathcal{P}_{XY}}\left[\ell(x,y,\theta)\right]$. Using again algorithmic stability (7), (8) later improve this bound to $\mathbb{E}[\mathcal{R}_\mathrm{P}]=\beta L\,\tilde{\mathcal{O}}(1/\sqrt{n}+\sqrt{p}/(n\varepsilon))$, where the expectation is taken with respect to the randomness of the training dataset and algorithm.

In the setting of (convex) *unconstrained optimization*, a line of work studies the problem of differentially private parameter estimation, in the case of mean-estimation (9), U-statistics (10), and M-estimators with strongly convex losses (11). While these results characterize the privacy cost via the distance from the true parameter ($\|\theta^p-\theta^*\|_2$ in our notation), (12, 13) are closer to our formulation as they also investigate the excess population risk in the setting of generalized linear models (GLMs). In particular, (13) removes the assumption on the strong convexity, sets θ^* to be the Bayes optimal solution, and denoting by M the projector on the column space of $\mathbb{E}_{x\sim\mathcal{P}_X}[\varphi(x)\varphi(x)^\top]$, improves the bound in (12) obtaining $\mathbb{E}[\mathcal{R}_\mathrm{P}]=L\,\|M\theta^*\|_2\;\tilde{\mathcal{O}}\left(1/\sqrt{n}+\sqrt{\min\left(\mathrm{rank}(M),n\right)/(\varepsilon n)}\right)$ (see their Theorem 3.2). Beyond the setting of GLMs, (14) considers Lipschitz losses with ℓ_2 regularization and introduces the notion of restricted Lipschitz continuity, which gives dimension-free bounds. However, in the setting of GLMs, this approach leads to the same bounds as in (13). A similar approach is taken in (15) that removes the dependence on p at the cost of an additional factor $\mathrm{tr}(\tilde{H})$, where $\tilde{H}\succeq\sup_\theta\nabla^2_\theta\,\mathbb{E}_{(x,y)\sim\mathcal{P}_{XY}}[\ell(x,y,\theta)]$. The work (16) considers GLMs with a loss that is not necessarily Lipschitz, and it also recovers the result of (13) in the Lipschitz case with $\varepsilon=\Omega(1)$.

The setting of *linear regression* reads $\ell(x,y,\theta)=(x^\top\theta-y)^2$, where $y=x^\top\theta^*+\sigma$, θ^* is the ground-truth solution and σ independent label noise. (17) assumes that each point is sampled from a standard Gaussian distribution and obtains a sample complexity of d/ε via gradient descent with adaptive clipping (18). This improves over (9, 19, 20), and it matches the result of earlier work in (21), which however proposes a computationally inefficient method. (22) relaxes the assumption on the data (sub-Weibull concentration) and improves the dependence of the sample complexity on the condition number of the covariance matrix. (23) removes the need for adaptive clipping, focusing on a setting where the clipping constant is large enough so that clipping will most likely never happen.

A. Evaluating previous bounds on the RF model. In the body we discussed how previous work in (12, 13) cannot successfully tackle the over-parameterized RF model, in the setting where $\varepsilon=\Theta(1)$. To do so, we characterized different quantities considered in their bounds, namely the Lipschitz constant of the loss with respect to the parameters L, the norm of the baseline solution $\|\theta^*\|_2$, the rank of the matrix M, defined as the projector on the column space of $\mathbb{E}_x[\varphi(x)\varphi(x)^\top]$, and the quantity $\|M\theta^*\|_2$. We will quantitatively discuss these in the following paragraphs.

Estimate of $\|\theta^*\|_2$. A quick upper-bound of this quantity can be obtained via the expression $\theta^*=\Phi^+Y$, which gives $\|\theta^*\|_2\le\left\|\Phi^+\right\|_{\mathrm{op}}\|Y\|_2$. Due to Lemma 4.5, we have that, with high probability, $\left\|\Phi^+\right\|_{\mathrm{op}}=\mathcal{O}\left(p^{-1/2}\right)$. Then, due to our hypothesis on the boundedness of the labels, we get $\|\theta^*\|_2=\mathcal{O}\left(\sqrt{n/p}\right)$. It turns out that this upper-bound can be shown to be tight in different settings, *e.g.* when the labels contain random noise independent from the data. In this case, an argument as the one in Theorem 2 of (24), together with our Lemma 4.5, would guarantee that $\|\theta^*\|_2=\Theta(\|\Phi^+\|_F)=\Theta(\sqrt{n/p})$.

Estimate of L via a bounded set $\mathcal{B}$ with radius $\beta=\Theta(\|\theta^*\|)$. Denoting with (x,y) a generic input-label pair we can write

$$L=\sup_{\theta\in\mathcal{B}}\left\|\nabla_\theta\ell(\varphi(x)^\top\theta-y)\right\|_2=\sup_{\theta\in\mathcal{B}}2\left\|\varphi(x)\right\|\left|\varphi(x)^\top\theta-y\right|=\Theta\left(\beta\left\|\varphi(x)\right\|_2^2\right), \tag{22}$$

where we used Cauchy-Schwartz in the last step, and the fact that $\|\varphi(x)\|_2\,\beta\gg|y|$ in our data scaling. Then, setting $\|\varphi(x)\|_2=\Theta(\sqrt{p})$ (which holds with high probability with respect to V, and both in expectation and in high probability

*The definition of empirical excess risk in (4) is not re-normalized by n. This makes the bounds of Theorem 2.4.2 therein larger by a factor n, which is removed here for the sake of comparison with other works.

with respect to x) and plugging the value $\beta = \Theta(\|\theta^*\|)$ obtained in the previous paragraph, we obtain $L = \Theta(\sqrt{np})$, and consequently $L \|\theta^*\|_2 = \Theta(n)$.

Estimate of L via the set $\mathcal{C}$. Alternatively, one could be interested in an RF model with Lipschitz loss, as the clipped loss defined in Eq. (10) with C_{clip} set as in Eq. (75). This choice would guarantee that the the Lipschitz constant of L of loss scales as, excluding poly-logarithmic factors, $\sqrt{p}$, which corresponds to the norm of the features $\|\varphi(x)\|_2$. In this setting, we can show that the solution of gradient flow on this loss corresponds to the solution found on the quadratic loss, as the dynamics fully happens in the set $\mathcal{C}$ where the two losses coincide (see Lemma 3.1). Thus, this allows to improve the previous estimate by a factor $\sqrt{n}$, as we now have $L \|\theta^*\|_2 = \Theta(\sqrt{p}\sqrt{n/p}) = \Theta(\sqrt{n})$, which is however not enough to obtain meaningful guarantees from (12).

Estimate of $\text{rank}(M)$ and $\|M\theta^*\|_2$ from (13). Defining with M_D the projector over the column space of $\Phi^\top\Phi$, under sufficient regularity of the data distribution $\mathcal{P}_X$, it can be shown that M_D spans a subspace of the one spanned by M (notice that this exact argument is used also in the proof of Theorem 3.2 of (13)). Then, since $M_D\theta^* = \theta^*$ and $\text{rank}(M_D) = n$ due to our Lemma 4.5, we also have that $\|M\theta^*\|_2 = \|\theta^*\|_2$ and $\min(\text{rank}(M), n) = n$. Then, in the over-parameterized RF model, the bound of (13) corresponds to the one in (12), and the previous discussion applies.

2. Proofs on Differential Privacy

We start by introducing the notion of *sensitivity*. Let $\mu : \mathcal{D} \to \mathbb{R}^p$ be an arbitrary, deterministic, p-dimensional function, where $\mathcal{D}$ represents the space of datasets. We define its ℓ_2 sensitivity to be

$$\Delta_2\mu = \sup_{D \text{ adjacent with } D'} \left\|\mu(D) - \mu(D')\right\|_2, \tag{23}$$

where D adjacent with D' means that the two datasets D and D' differ by only one training sample. The DP-GD algorithm is obtained through the composition of T independent Gaussian mechanisms (see (25), Theorem A.1), as every iteration includes a (clipped) gradient update and a Gaussian noise injection. In this case, we have that Algorithm 1 is a randomized mechanism $\mathcal{A}$ consisting of a sequence of adaptive (Gaussian) mechanisms $\mathcal{M}_1, \dots, \mathcal{M}_T$ where $\mathcal{M}_t : \mathbb{R}^p \times \mathcal{D} \to \mathbb{R}^p$ for all $t \in [T]$. One option is then to use the advanced composition Theorem (see Theorem 3.20 in (25)) to compute the privacy guarantees of the algorithm $\mathcal{A}$. Unfortunately, this approach is not fruitful in our case, as it would involve an additional $\log T$ term in our final result in Theorem 1.2, which would make the variance of the noise added at each iteration to diverge, in the limit of $\eta \to 0$, $T \to +\infty$, with $\eta T \to \tau$. Thus, to prove Proposition 1.2, we calculate the privacy guarantees through the moment accountant method, also used in (26) to show their Theorem 2 (which we restate in Theorem 2.1, for the sake of completeness and notation). This allows for a tighter tracking of the privacy budget, exploiting the independence between the Gaussian mechanisms at every iteration of Algorithm 1.

While our approach is conceptually similar to the one used in Theorem 1 of (26), in contrast with (26) we consider full batch gradient descent and make explicit the dependence on the learning rate η, given our choice of scaling for Algorithm 1. We rely on the notion of *privacy loss*, which describes the difference that a randomized mechanism has on two adjacent datasets. More specifically, given two adjacent datasets $D, D' \in \mathcal{D}$, a randomized mechanism $\mathcal{M}_t : \mathbb{R}^p \times \mathcal{D} \to \mathbb{R}^p$ and an auxiliary input $\theta_{t-1} \in \mathbb{R}^p$, the *privacy loss* at the output θ takes the form

$$\gamma(\theta; \mathcal{M}_t, \theta_{t-1}, D, D') = \log \frac{p\left(\mathcal{M}_t(\theta_{t-1}, D) = \theta\right)}{p\left(\mathcal{M}_t(\theta_{t-1}, D') = \theta\right)}, \tag{24}$$

where the notation $p\,(Z = \theta)$ indicates the value of the law of a random variable Z evaluated in θ. As in (26), we define the λ-th moment $\alpha_{\mathcal{M}_t}(\lambda; \theta_{t-1}, D, D')$ as the logarithm of the moment generating function of the privacy loss evaluated in λ, *i.e.*,

$$\alpha_{\mathcal{M}_t}(\lambda; \theta_{t-1}, D, D') = \log \mathbb{E}_{\theta \sim \mathcal{M}_t(\theta_{t-1}, D)} \left[\exp(\lambda\gamma(\theta; \mathcal{M}_t, \theta_{t-1}, D, D'))\right]. \tag{25}$$

Note that the expectation is taken with respect to the probability distribution of the parameters given by the mechanism applied to the dataset D. Then, we can define

$$\alpha_{\mathcal{M}_t}(\lambda) = \sup_{\theta_{t-1}, D \text{ adjacent with } D'} \alpha_{\mathcal{M}_t}(\lambda; \theta_{t-1}, D, D'), \tag{26}$$

where the supremum is taken over all possible θ_{t-1} and all the adjacent datasets D, D'.

We now re-state Theorem 2 of (26) which translates the moment accountant to (ε, δ) privacy guarantees. Next, we bound the sensitivity of each iteration of Algorithm 1 and, therefore, the corresponding value of $\alpha_{\mathcal{M}_t}(\lambda)$ (see Lemmas 2.1 and 2.2). The proof of Proposition 1.2 will then follow.

Theorem 2.1 (Theorem 2 of (26)). *Let $\mathcal{A}$ consist of a sequence of independent adaptive mechanisms $\mathcal{M}_1, \dots, \mathcal{M}_T$ where $\mathcal{M}_t : \mathbb{R}^p \times \mathcal{D} \to \mathbb{R}^p$. Let $\alpha_{\mathcal{M}_t}(\lambda)$ be defined according to* Eq. (26). *Then, the following properties hold.*

1. ***Composability.*** *For any λ,*

$$\alpha_{\mathcal{A}}(\lambda) \le \sum_{t=1}^{T} \alpha_{\mathcal{M}_t}(\lambda). \tag{27}$$

2. ***Tail bound.*** *For any* $\varepsilon > 0$, $\mathcal{A}$ *is* (ε, δ)*-differentially private for*

$$\delta = \inf_{\lambda} \exp(\alpha_{\mathcal{A}}(\lambda) - \lambda\varepsilon). \quad [28]$$

Lemma 2.1. *Consider the* t*-th iteration of Algorithm 1 on a dataset* D *without noise,* i.e.,

$$\mu_{\theta_{t-1},D} = \theta_{t-1} - \frac{\eta}{n} \sum_{(x_i,y_i)\in D} g_{C_{\mathrm{clip}}}(x_i, y_i, \theta_{t-1}). \quad [29]$$

Then, we have

$$\Delta_2\mu = \sup_{\theta_{t-1}\in\mathbb{R}^p, D \text{ adjacent with } D'} \left\|\mu_{\theta_{t-1},D} - \mu_{\theta_{t-1},D'}\right\|_2 \le \frac{2\eta C_{\mathrm{clip}}}{n}. \quad [30]$$

Proof. We have

$$\begin{aligned}
\Delta_2\mu &= \frac{\eta}{n} \sup_{\theta_{t-1}\in\mathbb{R}^p, D \text{ adjacent with } D'} \left\| \sum_{(x_i,y_i)\in D} g_{C_{\mathrm{clip}}}(x_i, y_i, \theta_{t-1}) - \sum_{(x_i,y_i)\in D'} g_{C_{\mathrm{clip}}}(x_i, y_i, \theta_{t-1}) \right\|_2 \\
&= \frac{\eta}{n} \sup_{\theta_{t-1}\in\mathbb{R}^p, (x,y),(x',y')\in(\mathcal{X},\mathcal{Y})} \left\|g_{C_{\mathrm{clip}}}(x, y, \theta_{t-1}) - g_{C_{\mathrm{clip}}}(x', y', \theta_{t-1})\right\|_2 \\
&\le \frac{\eta}{n} \sup_{\theta_{t-1}\in\mathbb{R}^p, (x,y),(x',y')\in(\mathcal{X},\mathcal{Y})} \left(\left\|g_{C_{\mathrm{clip}}}(x, y, \theta_{t-1})\right\|_2 + \left\|g_{C_{\mathrm{clip}}}(x', y', \theta_{t-1})\right\|_2\right) \\
&\le \frac{2\eta C_{\mathrm{clip}}}{n},
\end{aligned} \quad [31]$$

where the last inequality comes from $\left\|g_{C_{\mathrm{clip}}}(x, y, \theta_{t-1})\right\|_2 \le C_{\mathrm{clip}}$ for any $(x, y) \in (\mathcal{X}, \mathcal{Y})$ and any $\theta_{t-1} \in \mathbb{R}^p$. □

Lemma 2.2. *Let* $\mathcal{M}_t$ *be the randomized mechanism induced by the* t*-th iteration of Algorithm 1. Then, for every* $t \in [T]$ *we have*

$$\alpha_{\mathcal{M}_t}(\lambda) = \sup_{\theta_{t-1}, D \text{ adjacent with } D'} \alpha_{\mathcal{M}_t}(\lambda; \theta_{t-1}, D, D') \le \frac{\eta}{2\sigma^2}\left(\lambda + \lambda^2\right). \quad [32]$$

Proof. As $\mathcal{M}_t$ is a Gaussian mechanism with parameter $\varsigma = \sqrt{\eta}\,\frac{2C_{\mathrm{clip}}}{n}\sigma$, we have

$$p\left(\mathcal{M}_t(\theta_{t-1}, D) = \theta\right) = \frac{1}{\sqrt{(2\pi\varsigma^2)^p}} \exp\left(-\frac{\left\|\theta - \mu_{\theta_{t-1},D}\right\|_2^2}{2\varsigma^2}\right), \quad [33]$$

where $\mu_{\theta_{t-1},D}$ represents the update without noise

$$\mu_{\theta_{t-1},D} = \theta_{t-1} - \frac{\eta}{n} \sum_{(x_i,y_i)\in D} g_{C_{\mathrm{clip}}}(x_i, y_i, \theta_{t-1}). \quad [34]$$

Equivalently, Eq. (33) also holds for $\mathbb{P}\left(\mathcal{M}_t(\theta_{t-1}, D') = \theta\right)$, with $\mu_{\theta_{t-1},D'} = \theta_{t-1} - \eta g_{\theta_{t-1},D'}$. Then, we have that the privacy loss defined in Eq. (24) takes the form

$$\begin{aligned}
&\gamma(\theta; \mathcal{M}_t, \theta_{t-1}, D, D') \\
&= \log \frac{p\left(\mathcal{M}_t(\theta_{t-1}, D) = \theta\right)}{p\left(\mathcal{M}_t(\theta_{t-1}, D') = \theta\right)} \\
&= -\frac{1}{2\varsigma^2}\left(\left\|\theta - \mu_{\theta_{t-1},D}\right\|_2^2 - \left\|\theta - \mu_{\theta_{t-1},D'}\right\|_2^2\right) \\
&= -\frac{1}{2\varsigma^2}\left(2\theta^\top(\mu_{\theta_{t-1},D'} - \mu_{\theta_{t-1},D}) + \left\|\mu_{\theta_{t-1},D}\right\|_2^2 - \left\|\mu_{\theta_{t-1},D'}\right\|_2^2\right) \\
&= -\frac{1}{2\varsigma^2}\left(2(\theta - \mu_{\theta_{t-1},D})^\top(\mu_{\theta_{t-1},D'} - \mu_{\theta_{t-1},D}) - \left\|\mu_{\theta_{t-1},D}\right\|_2^2 + 2\mu_{\theta_{t-1},D}^\top\mu_{\theta_{t-1},D'} - \left\|\mu_{\theta_{t-1},D'}\right\|_2^2\right) \\
&= -\frac{1}{2\varsigma^2}\left(2(\theta - \mu_{\theta_{t-1},D})^\top\Delta_{\theta_{t-1},D,D'} - \left\|\Delta_{\theta_{t-1},D,D'}\right\|_2^2\right),
\end{aligned} \quad [35]$$

where we introduce the shorthand $\Delta_{\theta_{t-1},D,D'} = \mu_{\theta_{t-1},D'} - \mu_{\theta_{t-1},D}$. We can now compute the moment generating function of the privacy loss

$$\begin{aligned}
&\alpha_{\mathcal{M}_t}(\lambda; \theta_{t-1}, D, D') \\
&= \log \mathbb{E}_{\theta\sim\mathcal{M}_t(\theta_{t-1},D)} \left[\exp(\lambda\gamma(\theta; \mathcal{M}_t, \theta_{t-1}, D, D'))\right] \\
&= \log \left(\exp\left(\lambda \frac{\left\|\Delta_{\theta_{t-1},D,D'}\right\|_2^2}{2\varsigma^2}\right) \mathbb{E}_{\theta\sim\mathcal{M}(\theta_{t-1},D)} \left[\exp\left(-\lambda \frac{\left(\theta-\mu_{\theta_{t-1},D}\right)^\top \Delta_{\theta_{t-1},D,D'}}{\varsigma^2}\right)\right]\right) \\
&= \lambda \frac{\left\|\Delta_{\theta_{t-1},D,D'}\right\|_2^2}{2\varsigma^2} + \log \mathbb{E}_{\theta\sim\mathcal{N}(\mu_{\theta_{t-1},D},\varsigma^2 I_p)} \left[\exp\left(-\lambda \frac{\left(\theta-\mu_{\theta_{t-1},D}\right)^\top \Delta_{\theta_{t-1},D,D'}}{\varsigma^2}\right)\right] \\
&= \lambda \frac{\left\|\Delta_{\theta_{t-1},D,D'}\right\|_2^2}{2\varsigma^2} + \log \mathbb{E}_{\theta'\sim\mathcal{N}(0,I_p)} \left[\exp\left(-\lambda \frac{\theta'^\top \Delta_{\theta_{t-1},D,D'}}{\varsigma}\right)\right] \\
&= \lambda \frac{\left\|\Delta_{\theta_{t-1},D,D'}\right\|_2^2}{2\varsigma^2} + \log \mathbb{E}_{\rho\sim\mathcal{N}(0,1)} \left[\exp\left(-\lambda \frac{\left\|\Delta_{\theta_{t-1},D,D'}\right\|_2}{\varsigma}\rho\right)\right] \\
&= \lambda \frac{\left\|\Delta_{\theta_{t-1},D,D'}\right\|_2^2}{2\varsigma^2} + \lambda^2 \frac{\left\|\Delta_{\theta_{t-1},D,D'}\right\|_2^2}{2\varsigma^2} \\
&= \frac{\left\|\Delta_{\theta_{t-1},D,D'}\right\|_2^2}{2\varsigma^2} \left(\lambda+\lambda^2\right),
\end{aligned} \tag{36}$$

where we perform a change of variable in the fifth line, use the rotational invariance of the Gaussian distribution in the sixth line, and compute the moment generating function of a standard Gaussian on the seventh line. Thus, we can write

$$\alpha_{\mathcal{M}_t}(\lambda) = \sup_{\theta_{t-1},D \text{ adjacent with } D'} \alpha_{\mathcal{M}_t}(\lambda; \theta_{t-1}, D, D') = \frac{(\Delta_2\mu)^2}{2\varsigma^2} \left(\lambda+\lambda^2\right), \tag{37}$$

where

$$\Delta_2\mu = \sup_{\theta_{t-1},D \text{ adjacent with } D'} \left\|\Delta_{\theta_{t-1},D,D'}\right\|_2 \tag{38}$$

is the ℓ_2 sensitivity of the gradient updates in Algorithm 1. By Lemma 2.1, we have that $\Delta_2\mu \leq 2\eta C_{\text{clip}}/n$. Thus, plugging $\varsigma = \sqrt{\eta}\,\frac{2C_{\text{clip}}}{n}\sigma$ in Eq. (37), we readily get the desired result. □

Proof of Proposition 1.2. Algorithm 1 is a randomized algorithm $\mathcal{A}$ consisting of a sequence of independent adaptive mechanisms $\mathcal{M}_1, \dots, \mathcal{M}_T$ such that, for all $t \in [T]$, by Lemma 2.2, we have

$$\alpha_{\mathcal{M}_t}(\lambda) \leq \frac{\eta}{2\sigma^2}\left(\lambda+\lambda^2\right). \tag{39}$$

Thus, by composability (see Theorem 2.1), we have

$$\alpha_{\mathcal{A}}(\lambda) \leq \frac{\eta T}{2\sigma^2}\left(\lambda+\lambda^2\right). \tag{40}$$

Set $\delta \in (0,1)$ and $\varepsilon \in (0, 8\log(1/\delta))$, and suppose that

$$\frac{\eta T}{2\sigma^2} \leq \frac{\varepsilon^2}{16\log(1/\delta)}. \tag{41}$$

Then, considering $\lambda > 0$, we have that

$$\begin{aligned}
\exp\left(\alpha_{\mathcal{A}}(\lambda) - \lambda\varepsilon\right) &\leq \exp\left(\frac{\varepsilon^2}{16\log(1/\delta)}\left(\lambda+\lambda^2\right) - \lambda\varepsilon\right) \\
&\leq \exp\left(\frac{\varepsilon^2}{16\log(1/\delta)}\lambda^2 - \left(1 - \frac{\varepsilon}{16\log(1/\delta)}\right)\lambda\varepsilon\right) \\
&\leq \exp\left(\frac{\varepsilon^2}{16\log(1/\delta)}\lambda^2 - \frac{\lambda\varepsilon}{2}\right).
\end{aligned} \tag{42}$$

Setting $\lambda^* = 4\log(1/\delta)/\varepsilon$ we get

$$\begin{aligned}
\exp\left(\alpha_{\mathcal{A}}(\lambda^*) - \lambda^*\varepsilon\right) &\leq \exp\left(\frac{\varepsilon^2}{16\log(1/\delta)}\lambda^{*2} - \frac{\lambda^*\varepsilon}{2}\right) \\
&= \exp\left(\log(1/\delta) - 2\log(1/\delta)\right) \\
&= \exp\left(-\log(1/\delta)\right) = \delta.
\end{aligned} \tag{43}$$

Then, by the tail bound in Theorem 2.1, we have that $\mathcal{A}$ is (ε, δ)-differentially private. In our argument, we consider $\delta \in (0, 1)$, $\varepsilon \in (0, 8\log(1/\delta))$, and the inequality in Eq. (41), which can be rewritten as

$$\sigma \geq \sqrt{\eta T}\frac{\sqrt{8\log(1/\delta)}}{\varepsilon}, \tag{44}$$

which readily gives the desired result. □

Notice that stricter assumptions on the range of values allowed for ε would allow to improve the previous inequality, *i.e.*, to reduce the amount of required noise to guarantee (ε, δ)-DP. As our analysis does not track multiplicative, absolute constants, we will refrain from doing this in our work.

We now move to the proof that the clipping step in DP-GD can be replaced by the auxiliary clipped loss, defined via Eq. (10), here formally presented as Proposition 2.3. We then present the auxiliary Proposition 2.4 that will be useful in the discussion on the SDE defined in Eq. (12). Next, in Proposition 2.5 we formalize the convergence of Algorithm 1 in the limit of small learning rates $\eta \to 0$. To conclude, we state the auxiliary Lemma 2.6, useful for the final proof of Proposition 2.7, which will guarantee that the solution $\Theta(\tau)$ of Eq. (12) is (ε, δ)-DP.

Proposition 2.3. *For any $\theta \in \mathbb{R}^p$ and any clipping constant $C_{\text{clip}} > 0$, we have that $g_{C_{\text{clip}}}(x_i, y_i, \theta) = \nabla_\theta \ell_{i, C_{\text{clip}}}\left(\varphi(x_i)^\top \theta_{t-1} - y_i\right)$, where $g_{C_{\text{clip}}}(x_i, y_i, \theta)$ is defined in the clipping step of Algorithm 1.*

Proof. At every iteration t of Algorithm 1, we have

$$\begin{aligned} g_{C_{\text{clip}}}(x_i, y_i, \theta_{t-1}) &= g(x_i, y_i, \theta_{t-1}) / \max\left(1, \frac{\|g(x_i, y_i, \theta_{t-1})\|_2}{C_{\text{clip}}}\right) \\ &= \frac{\varphi(x_i)\ell'(\varphi(x_i)^\top \theta_{t-1} - y_i)}{\max\left(1, \frac{\|\varphi(x_i)\|_2 \left|\ell'\left(\varphi(x_i)^\top \theta_{t-1} - y_i\right)\right|}{C_{\text{clip}}}\right)} \\ &= \varphi(x_i)\ell'_{i, C_{\text{clip}}}\left(\varphi(x_i)^\top \theta_{t-1} - y_i\right) \\ &= \nabla_\theta \ell_{i, C_{\text{clip}}}\left(\varphi(x_i)^\top \theta_{t-1} - y_i\right). \quad \square \end{aligned} \tag{45}$$

□

Proposition 2.4. *Let $\ell : \mathbb{R} \to \mathbb{R}$ be a differentiable function with Lipschitz-continuous derivative. Then, for all $\theta \in \mathbb{R}^p$, we have*

$$\left\|\nabla \mathcal{L}_{C_{\text{clip}}}(\theta)\right\|_2 \leq C_{\text{clip}}. \tag{46}$$

Furthermore, there exists a constant K such that, for all $\theta, \theta' \in \mathbb{R}^p$, we have

$$\left\|\nabla \mathcal{L}_{C_{\text{clip}}}(\theta) - \nabla \mathcal{L}_{C_{\text{clip}}}(\theta')\right\|_2 \leq K \left\|\theta - \theta'\right\|_2, \tag{47}$$

with $K \leq L \sum_{i=1}^n \|\varphi(x_i)\|_2^2 / n$, where L is the Lipschitz constant of ℓ'.

Proof. Following the definition in Eq. (10), we have that, for all $i \in [n]$,

$$\nabla_\theta \ell_{i, C_{\text{clip}}}\left(\varphi(x_i)^\top \theta - y_i\right) = \nabla_\theta \ell\left(\varphi(x_i)^\top \theta - y_i\right) / \max\left(1, \frac{\left\|\nabla_\theta \ell\left(\varphi(x_i)^\top \theta - y_i\right)\right\|_2}{C_{\text{clip}}}\right), \tag{48}$$

which readily gives

$$\left\|\nabla_\theta \ell_{i, C_{\text{clip}}}\left(\varphi(x_i)^\top \theta - y_i\right)\right\|_2 \leq C_{\text{clip}}. \tag{49}$$

Then, we can conclude

$$\begin{aligned} \left\|\nabla \mathcal{L}_{C_{\text{clip}}}(\theta)\right\|_2 &= \left\|\nabla_\theta \frac{1}{n}\sum_{i=1}^n \ell_{i, C_{\text{clip}}}(\varphi(x_i)^\top \theta - y_i)\right\|_2 \\ &\leq \frac{1}{n}\sum_{i=1}^n \left\|\nabla_\theta \ell_{i, C_{\text{clip}}}(\varphi(x_i)^\top \theta - y_i)\right\|_2 \\ &\leq C_{\text{clip}}. \end{aligned} \tag{50}$$

For the second part of the claim, following a similar argument as before and exploiting the chain-rule, it is sufficient to show that, for every $i \in [n]$, $\ell_{i, C_{\text{clip}}}$ has Lipschitz-continuous derivative.

For all $z \in \mathbb{R}$ such that $\ell'(z) > 0$, we can write $\ell'_{i,C_{\text{clip}}}(z) = \min\left(\ell'(z), C_{\text{clip}}/\left\|\varphi(x_i)\right\|_2\right)$. Then, if ℓ' is Lipschitz-continuous, also $\ell'_{i,C_{\text{clip}}}$ is, as it can be written as composition of Lipschitz-continuous functions. A similar argument holds for all $z \in \mathbb{R}$ such that $\ell'(z) < 0$. Furthermore, for all z such that $|\ell'(z)| < C_{\text{clip}}/\left\|\varphi(x_i)\right\|_2$, we simply have $\ell'_{i,C_{\text{clip}}} = \ell'$. Thus, to prove that $\ell'_{i,C_{\text{clip}}}$ is Lipschitz-continuous, it suffices that ℓ' is Lipschitz-continuous, as assumed by the proposition. Notice that this argument also proves that the Lipschitz constant of $\ell'_{i,C_{\text{clip}}}$ is smaller or equal than the Lipschitz constant L of ℓ'. Thus, we have

$$\begin{aligned}\left\|\nabla\mathcal{L}_{C_{\text{clip}}}(\theta) - \nabla\mathcal{L}_{C_{\text{clip}}}(\theta')\right\|_2 &= \left\|\nabla\frac{1}{n}\sum_{i=1}^{n}\ell_{i,C_{\text{clip}}}(\varphi(x_i)^\top\theta - y_i) - \nabla\frac{1}{n}\sum_{i=1}^{n}\ell_{i,C_{\text{clip}}}(\varphi(x_i)^\top\theta' - y_i)\right\|_2 \\ &= \left\|\frac{1}{n}\sum_{i=1}^{n}\varphi(x_i)\left(\ell'_{i,C_{\text{clip}}}(\varphi(x_i)^\top\theta - y_i) - \ell'_{i,C_{\text{clip}}}(\varphi(x_i)^\top\theta' - y_i)\right)\right\|_2 \\ &\le \frac{1}{n}\sum_{i=1}^{n}\left\|\varphi(x_i)\right\|_2\left|\ell'_{i,C_{\text{clip}}}(\varphi(x_i)^\top\theta - y_i) - \ell'_{i,C_{\text{clip}}}(\varphi(x_i)^\top\theta' - y_i)\right| \\ &\le \frac{L}{n}\sum_{i=1}^{n}\left\|\varphi(x_i)\right\|_2^2\left\|\theta - \theta'\right\|_2,\end{aligned}$$

which proves the last statement. □

Due to Proposition 2.4, the SDE in Eq. (12) satisfies the assumptions A1-4 in Section 4.5 of (27). This means that Eq. (12) admits a unique strong solution $\Theta(t)$ (see Theorem 4.5.3 in (27)). Furthermore, we also have that the assumptions in (28) (see their Equations (1.5), (1.6), (1.7)) are verified, implying that at every fixed time $t > 0$, the probability density function $p(\Theta(t))$ is continuous with respect to the coordinate θ (see Theorem 1.2 in (28)). This property will be used later in Lemma 2.6 and Proposition 2.7.

In Section 3 of the body we mentioned that Eq. (11) is the Euler-Maruyama discretization of Eq. (12). This can be formalized defining a family of random variables $\{\theta_T^1, \theta_T^2, \dots,\}$, where θ_T^j is the output of Algorithm 1, with learning rate $\eta_j := \eta/j$, and number of iterations $T_j := jT$, with j being a positive integer. Algorithm 1 is defined such that, when performing the t-th iteration, we introduce the Gaussian random variable

$$\frac{2C_{\text{clip}}}{n}\sigma\left(B(t\eta/j) - B((t-1)\eta/j)\right), \tag{51}$$

where $B(t)$ is the standard p-dimensional Wiener process in Eq. (12). This is coherent with the definition of Algorithm 1, since these random variables are distributed as

$$\frac{2C_{\text{clip}}}{n}\sigma\mathcal{N}(0, \eta I/j) = \sqrt{\eta_j}\frac{2C_{\text{clip}}}{n}\sigma\mathcal{N}(0, I), \tag{52}$$

and they are independent with each other. Since Eq. (12) respects all the necessary assumptions in Theorem 10.2.2 in (27), we are ready to state the following:

Proposition 2.5. *Let $\tau = \eta T$. Then, for every $h > 0$, there exists j^*, such that for all $j > j^*$, we have*

$$\mathbb{E}\left[\left\|\theta_T^j - \Theta(\tau)\right\|_2\right] < h. \tag{53}$$

Proposition 2.5 formalizes in which sense the SDE in Eq. (12) is a continuous limit of Algorithm 1. This is done through the family of algorithms with progressively smaller learning rates and larger number of iterations. It is important to notice that all these algorithms provide the same DP guarantee, as $\eta_j T_j = \eta T$ for every j, and the result of Proposition 1.2 depends on the learning rate and number of iterations only through their product ηT.

Lemma 2.6. *We have that, for any open ball $S \subseteq \mathbb{R}^p$,*

$$\lim_{j\to\infty}\mathbb{P}\left(\theta_T^j \in S\right) = \mathbb{P}\left(\Theta_\tau \in S\right). \tag{54}$$

Proof. By contradiction, let the thesis be false. Then, there exists a ball $S(r_0, r)$ with center r_0 and radius r s.t. there is $h^* > 0$ and j arbitrarily large that satisfy

$$\left|\mathbb{P}\left(\theta_T^j \in S(r_0, r)\right) - \mathbb{P}\left(\Theta_\tau \in S(r_0, r)\right)\right| > h^*. \tag{55}$$

We first suppose that we have

$$\mathbb{P}\left(\theta_T^j \in S(r_0, r)\right) > \mathbb{P}\left(\Theta_\tau \in S(r_0, r)\right) + h^*, \tag{56}$$

for j arbitrarily large.

Then, there is a sequence of events ω_j s.t. $\mathbb{P}(\omega_j) > h^*$ and

$$\theta_T^j(\omega_j) \in S(r_0, r), \qquad \Theta_\tau(\omega_j) \notin S(r_0, r). \tag{57}$$

As mentioned previously in this section, the law $p(\Theta_\tau)$ is continuous, and therefore bounded in the closed set $S(r_0, r')$ for any r'. This implies that there exists $r^* > 0$, such that

$$\mathbb{P}\left(\Theta_\tau \notin S(r_0, r) \text{ and } \Theta_\tau \in S(r_0, r + r^*)\right) < \frac{h^*}{2}, \tag{58}$$

which in turn implies that there is a sequence of events ω_j^*, such that $\mathbb{P}\left(\omega_j^*\right) > h^*/2$ and

$$\theta_T^j(\omega_j^*) \in S(r_0, r), \qquad \Theta_\tau(\omega_j^*) \notin S(r_0, r + r^*). \tag{59}$$

Note that, for all such events, by triangle inequality, we have

$$\begin{aligned} \left\|\theta_T^j(\omega_j^*) - \Theta_\tau(\omega_j^*)\right\|_2 &\geq \left\|\Theta_\tau(\omega_j^*) - r_0\right\|_2 - \left\|\theta_T^j(\omega_j^*) - r_0\right\|_2 \\ &\geq (r + r^* - r_0) - (r - r_0) \\ &= r^*, \end{aligned} \tag{60}$$

which implies

$$\mathbb{E}\left[\left\|\theta_T^j - \Theta_\tau\right\|_2\right] \geq \mathbb{P}\left(\omega_j^*\right) \mathbb{E}\left[\left\|\theta_T^j - \Theta_\tau\right\|_2 \middle| \omega_j^*\right] \geq \frac{h^* r^*}{2}. \tag{61}$$

This last equation holds for arbitrarily large j, which provides the desired contradiction with Proposition 2.5.

If instead of Eq. (56) we have

$$\mathbb{P}\left(\Theta_\tau \in S(r_0, r)\right) > \mathbb{P}\left(\theta_T^j \in S(r_0, r)\right) + h^*, \tag{62}$$

for j arbitrarily large, then the argument follows in the same way. In fact, we exploit the continuity of the law $p(\Theta_\tau)$ to state that there exists $r^* > 0$, such that

$$\mathbb{P}\left(\Theta_\tau \notin S(r_0, r - r^*) \text{ and } \Theta_\tau \in S(r_0, r)\right) < \frac{h^*}{2}, \tag{63}$$

thus giving a sequence of events ω_j^*, such that $\mathbb{P}(\omega_j) > h^*/2$ and

$$\theta_T^j(\omega_j^*) \notin S(r_0, r), \qquad \Theta_\tau(\omega_j^*) \in S(r_0, r - r^*). \tag{64}$$

At this point, we can again apply the triangle inequality to obtain the same contradiction as in Eq. (61). □

Proposition 2.7. *Let $\ell : \mathbb{R} \to \mathbb{R}$ be a differentiable function with Lipschitz-continuous derivative. Then, for any $\delta \in (0, 1)$, $\varepsilon \in (0, 8\log(1/\delta))$, if we set*

$$\Sigma \geq \frac{2C_{\text{clip}}\sqrt{\tau}}{n} \frac{\sqrt{8\log(1/\delta)}}{\varepsilon}, \tag{65}$$

the solution $\Theta(\tau)$ of the SDE Eq. (12) *at time τ is (ε, δ)-differentially private.*

Proof. The assumption on Σ guarantees, due to Proposition 1.2, that for all $j > 0$, the discretizations θ_T^j of the SDE Eq. (12) are, for any $\delta \in (0, 1)$ and $\varepsilon \in (0, 8\log(1/\delta))$, (ε, δ)-differentially private, since

$$\sigma = \frac{n}{2C_{\text{clip}}}\Sigma \geq \sqrt{\tau}\frac{\sqrt{8\log(1/\delta)}}{\varepsilon} = \sqrt{\eta_j T_j}\frac{\sqrt{8\log(1/\delta)}}{\varepsilon}, \tag{66}$$

where the first step is due Eq. (65).

By contradiction, let's suppose Θ_τ is instead not (ε, δ)-DP. This means that there exists a point in the parameters space $\bar{\theta}$ such that, on adjacent datasets D and D', we have

$$p\left(\Theta_\tau(D) = \bar{\theta}\right) > e^\varepsilon p\left(\Theta_\tau(D') = \bar{\theta}\right) + \delta. \tag{67}$$

Since both the laws in the previous equation are continuous, we also have that there exists an open ball S centered in $\bar{\theta}$, such that

$$\mathbb{P}\left(\Theta_\tau(D) \in S\right) > e^\varepsilon \mathbb{P}\left(\Theta_\tau(D') \in S\right) + \delta, \tag{68}$$

which we rewrite as

$$\mathbb{P}\left(\Theta_\tau(D) \in S\right) = e^\varepsilon \mathbb{P}\left(\Theta_\tau(D') \in S\right) + \delta + h, \tag{69}$$

for some $h > 0$. Now, by Lemma 2.6, we have that there exists j^* large enough s.t.

$$\left|\mathbb{P}\left(\theta_T^{j^*}(D) \in S\right) - \mathbb{P}\left(\Theta_\tau(D) \in S\right)\right| < \frac{h}{2}, \qquad [70]$$

and

$$\left|\mathbb{P}\left(\theta_T^{j^*}(D') \in S\right) - \mathbb{P}\left(\Theta_\tau(D') \in S\right)\right| < \frac{he^{-\varepsilon}}{2}. \qquad [71]$$

Hence, putting together Eq. (69), Eq. (70), and Eq. (71), we have

$$\begin{aligned}\mathbb{P}\left(\theta_T^{j^*}(D) \in S\right) &> \mathbb{P}\left(\Theta_\tau(D) \in S\right) - \frac{h}{2} \\ &= e^{\varepsilon}\mathbb{P}\left(\Theta_\tau(D') \in S\right) + \delta + h - \frac{h}{2} \\ &> e^{\varepsilon}\left(\mathbb{P}\left(\theta_T^{j^*}(D') \in S\right) - \frac{he^{-\varepsilon}}{2}\right) + \delta + h - \frac{h}{2} \\ &= e^{\varepsilon}\mathbb{P}\left(\theta_T^{j^*}(D') \in S\right) + \delta.\end{aligned} \qquad [72]$$

The previous equation implies that the discretization $\theta_T^{j^*}$ is not (ε, δ)-DP, therefore contradicting the thesis of Theorem 1.2. This provides the desired result. □

3. Proof of Theorem 1

Notation. Random variables are defined on different probability spaces, which describe the randomness in the data, the random features and the private mechanisms. All complexity notations are understood for sufficiently large data size n, input dimension d and number of parameters p. We indicate with C and c absolute, strictly positive, numerical constants, that do not depend on the scalings of the problem and whose value may change from line to line. Given a positive number s, $[s]$ denotes the set of positive numbers from 1 to s. Given a vector v, $\|v\|_2$ denotes its Euclidean norm. Given a matrix A, $\|A\|_{\text{op}}$ denotes its operator norm, $\|A\|_F$ its Frobenius norm and A^+ its Moore-Penrose inverse. We use $X \in \mathbb{R}^{n\times d}$ to denote the data matrix (containing the i-th element of the training set $x_i \in \mathbb{R}^d$ in its i-th row), $\Phi \in \mathbb{R}^{n\times p}$ to denote the feature matrix (containing $\varphi(x_i) := \phi(Vx) \in \mathbb{R}^p$ in its i-th row) and $K := \Phi\Phi^\top \in \mathbb{R}^{n\times n}$ to denote the kernel associated with the feature map.

In the following, for convenience, we will re-state all the technical assumptions required for the main result, corresponding to the problem setup in Section 1 of the body.

Assumption 1 (Data distribution). *The training samples* $\{(x_1, y_1), \dots, (x_n, y_n)\}$ *are* n *i.i.d. samples from the joint distribution* $\mathcal{P}_{XY}$*, such that the marginal distribution* $\mathcal{P}_X$ *satisfies the following properties:*

1. $x \sim \mathcal{P}_X$ *is sub-Gaussian, with* $\|x\|_{\psi_2} = \mathcal{O}(1)$.
2. *The data* $x \sim \mathcal{P}_X$ *are normalized such that* $\|x\|_2 = \sqrt{d}$.
3. $\lambda_{\min}\left(\mathbb{E}_{x\sim\mathcal{P}_X}\left[xx^\top\right]\right) = \Omega(1)$, i.e., *the second-moment matrix of the data is well-conditioned.*

Furthermore, we assume all the labels $(y_1, \dots, y_n)$ *to be bounded.*

Assumption 2 (Activation function). *The activation function* $\phi : \mathbb{R} \to \mathbb{R}$ *is a non-linear, Lipschitz continuous function such that* $\mu_0 = \mu_2 = 0$ *and* $\mu_1 \neq 0$*, where* μ_k *denotes the* k*-th Hermite coefficient of* ϕ.

Assumption 3 (Parameter scaling).

$$n = \mathcal{O}\left(\sqrt{p}\right), \qquad \log n = \Theta\left(\log p\right), \qquad n = \omega\left(d\log^2 d\right), \qquad n = o\left(\frac{d^{3/2}}{\log^3 d}\right). \qquad [73]$$

Assumption 4 (Privacy budget).

$$\delta \in (0,1), \qquad \varepsilon \in (0, 8\log(1/\delta)), \qquad \frac{\varepsilon}{\sqrt{\log(1/\delta)}} = \omega\left(\frac{d\log^5 n}{n}\right). \qquad [74]$$

The statement of our main result will address the random variable $\Theta(\tau)$ defined by the continuous process in Eq. (12), obtained as the limit of Algorithm 1 for $\eta \to 0$. This random variable is defined via the hyper parameters

$$\tau = \frac{d\log^2 n}{p}, \qquad C_{\text{clip}} = \sqrt{p}\log^2 n, \qquad \Sigma = \frac{2C_{\text{clip}}\sqrt{\tau}}{n}\frac{\sqrt{8\log(1/\delta)}}{\varepsilon}. \qquad [75]$$

Theorem (cf. Theorem 1). *Consider the RF model in* Eq. (2) *with input dimension d and number of features p. Let n be the number of training samples and $\mathcal{R}_{\rm P}$ be defined in* Eq. (1)*, where θ^* is given by* Eq. (9) *and θ^p is the solution $\Theta(\tau)$ of the SDE* Eq. (12) *at time τ, with hyper-parameters set as in* Eq. (75)*. Let Assumptions 1, 2, 3, 4 hold. Then, we have that θ^p is (ε, δ)-differentially private, and that*

$$
\begin{aligned}
|\mathcal{R}_P| &= \mathcal{O}\left(\frac{d}{n\varepsilon}\log^5 n\sqrt{\log(1/\delta)} + \sqrt{\frac{d}{n}} + \sqrt{\frac{n\log^3 d}{d^{3/2}}}\right) \\
&= \tilde{\mathcal{O}}\left(\frac{d}{n\varepsilon} + \sqrt{\frac{d}{n}} + \sqrt{\frac{n}{d^{3/2}}}\right) = o(1),
\end{aligned}
\tag{76}
$$

with probability at least $1 - 2\exp\left(-c\log^2 n\right)$, where c is an absolute constant.

The strong convergence of the Euler-Maruyama discretization scheme guarantees that, for sufficiently small learning rates η, the solution θ_T of Algorithm 1 also satisfies Eq. (76), which establishes the utility of DP-GD. Then, in this section, we prove the upper bound on $\mathcal{R}_{\rm P}$ considering $\theta^p := \Theta(\tau)$, as specified in the previous statement, as this will in turn address the statement as reported in Section 1 of the body.

Technical outline. As discussed in Section 3 of the body, we circumvent the difficulty in explicitly solving the SDE in Eq. (12) (*i.e.*, in characterizing the probability density function of $\Theta(\tau)$) considering the SDE

$$
\mathrm{d}\hat{\Theta}(t) = -\nabla\mathcal{L}(\hat{\Theta}(t))\mathrm{d}t + \Sigma\,\mathrm{d}B(t) = -\frac{2\Phi^\top}{n}\left(\Phi\hat{\Theta}(t) - Y\right)\mathrm{d}t + \Sigma\,\mathrm{d}B(t),
\tag{77}
$$

where $\mathcal{L}(\theta)$ is the original (quadratic) training loss and $B(t)$ is the same standard Wiener process as in Eq. (12). The solution of the SDE in Eq. (77) is a multi-dimensional Ornstein–Uhlenbeck (OU) process which admits a closed form (see, e.g., Section 4.4.4 in (29)). Let us then define

$$
\mathcal{C} := \left\{\theta \quad \text{s.t.} \quad \|\nabla_\theta \ell(\varphi(x_i)^\top\theta - y_i)\|_2 < C_{\rm clip} \quad \text{for all } i \in [n]\right\},
\tag{78}
$$

which corresponds to the subset of the parameters space where *clipping does not happen*, *i.e.*, where $\mathcal{L}_{C_{\rm clip}}(\theta) = \mathcal{L}(\theta)$. If the full path of the process $\Theta(t)$ happens in this region (*i.e.* $\Theta(t) \in \mathcal{C}$ for all $t \in [0, \tau]$), then $\Theta(\tau) = \hat{\Theta}(\tau)$. This corresponds to the event

$$
\hat{\Theta}(t) \in \mathcal{C}, \text{ for all } t \in [0, \tau],
\tag{79}
$$

which is easier to control, as $\hat{\Theta}(t)$ is an OU process. The first part of our proof consists in showing that, for our choice of the hyper-parameters in Eq. (75), this event happens with high probability. To do so, we consider the decomposition

$$
\hat{\Theta}(t) = \mathbb{E}_B\left[\hat{\Theta}(t)\right] + \tilde{\Theta}(t) = \hat{\theta}(t) + \tilde{\Theta}(t),
\tag{80}
$$

where we introduce the notation $\tilde{\Theta}(t) := \hat{\Theta}(t) - \mathbb{E}_B[\hat{\Theta}(t)]$ and use that the expectation of a OU process corresponds to the gradient flow $\mathbb{E}_B[\hat{\Theta}(t)] = \hat{\theta}(t)$ defined in Eq. (9). Then, $\mathcal{C}$ can be characterized as

$$
\begin{aligned}
\mathcal{C} &:= \left\{\theta \quad \text{s.t.} \quad \left\|\nabla_\theta \ell(\varphi(x_i)^\top\theta - y_i)\right\|_2 < C_{\rm clip} \quad \text{for all } i \in [n]\right\} \\
&= \left\{\theta \quad \text{s.t.} \quad \left|\varphi(x_i)^\top\theta - y_i\right| < \frac{C_{\rm clip}}{2\left\|\varphi(x_i)\right\|_2} \quad \text{for all } i \in [n]\right\},
\end{aligned}
\tag{81}
$$

which implies that the probability of the event in Eq. (79) is lower bounded by the probability of the event

$$
\left|\varphi(x_i)^\top\tilde{\Theta}(t)\right| + \left|\varphi(x_i)^\top\hat{\theta}(t) - y_i\right| \le \frac{C_{\rm clip}}{2\left\|\varphi(x_i)\right\|_2}, \quad \text{for all } i \in [n], \quad \text{for all } t \in [0, \tau].
\tag{82}
$$

As we set $C_{\rm clip} = \sqrt{p}\log^2 n$ (see Eq. (75)) and $\left\|\varphi(x_i)\right\|_2 = \Theta(\sqrt{p})$ with high probability (see the argument in Eq. (295) and Eq. (296)), we therefore wish to show that, for all $i \in [n]$ and for all $t \in [0, \tau]$, $|\varphi(x_i)^\top\tilde{\Theta}(t)| = o(\log^2 n)$ and $|\varphi(x_i)^\top\hat{\theta}(t) - y_i| = o(\log^2 n)$ with high probability. The term $|\varphi(x_i)^\top\hat{\theta}(t) - y_i|$ is handled by the lemma below.

Lemma 3.1. *Let Assumptions 1, 2 and 3 hold. Then, we have that, jointly for all $i \in [n]$,*

$$
\sup_{t\in[0,\tau]}\left|\varphi(x_i)^\top\hat{\theta}(t) - y_i\right| = \mathcal{O}\left(\log n\right),
\tag{83}
$$

with probability at least $1 - 2\exp\left(-c\log^2 n\right)$ over V and X, where c is an absolute constant.

We now briefly sketch the argument to obtain Eq. (83), deferring the full proof to Section 3-A of the SI. As the initialization $\theta_0 = 0$, the solution of the gradient flow in Eq. (9) takes the form $\hat{\theta}(t) = (1 - e^{-\frac{2\Phi^\top \Phi}{n} t})\Phi^+ Y$. Since K is invertible with high probability (due to Lemma 4.5), the quantity of interest can be expressed as

$$y_i - \varphi(x_i)^\top \hat{\theta}(t) = \varphi(x_i)^\top e^{-\frac{2\Phi^\top \Phi}{n} t} \Phi^+ Y.$$

Our approach consists in iteratively controlling this quantity introducing auxiliary *leave-one-out* variables, *i.e.*, $\Phi_{-i} \in \mathbb{R}^{(n-1)\times p}$ and $Y_{-i} \in \mathbb{R}^{n-1}$, defined without the i-th sample. The first step involves showing that

$$\left\| \Phi^+ Y - \Phi_{-i}^+ Y_{-i} \right\|_2 = \tilde{\mathcal{O}}\left(p^{-1/2}\right),$$

which implies that we can focus the analysis on $\sup_{t\in[0,\tau]} |\varphi(x_i)^\top e^{-\frac{2\Phi^\top \Phi}{n} t} \Phi_{-i}^+ Y_{-i}|$. This result comes from the *stability* of GD, as shown in Lemma 3.5, and is a consequence of lower bounding the smallest eigenvalue of the kernel $\lambda_{\min}(K)$.

The second step aims to introduce a leave-one-out variable in the exponent, as we show that

$$\sup_{t\in[0,\tau]} \left| \varphi(x_i)^\top e^{-\frac{2\Phi^\top \Phi}{n} t} \Phi_{-i}^+ Y_{-i} \right| \le 2 \sup_{t\in[0,\tau]} \left| \varphi(x_i)^\top e^{-\frac{2\Phi_{-i}^\top \Phi_{-i}}{n} t} \Phi_{-i}^+ Y_{-i} \right|.$$

This result is achieved via an explicit computation based on *Lie's product formula* for the matrix exponential, which is carried through in Lemma 3.7.

Finally, since the only dependence on x_i is via the term $\varphi(x_i)^\top$, we can conclude the argument with *Dudley's (chaining tail) inequality*, upper bounding the covering number of the set described by the curve $\gamma(t) = V^\top e^{-\frac{2\Phi_{-i}^\top \Phi_{-i}}{n} t} \Phi_{-i}^+ Y_{-i}$ in Lemma 3.8.

Next, we handle the term $|\varphi(x_i)^\top \tilde{\Theta}(t)|$ via the lemma below, also proven in Section 3-A of the SI.

Lemma 3.2. *Let Assumptions 1, 2, and 4 hold. Then, we have that, jointly for all $i \in [n]$,*

$$\sup_{t\in[0,\tau]} \left| \varphi(x_i)^\top \tilde{\Theta}(t) \right| = \mathcal{O}(\log n), \qquad [84]$$

with probability at least $1 - 2\exp\left(-c\log^2 n\right) - 2n\exp(-cp)$ over B and V, where c is an absolute constant and B refers to the probability space of the private mechanism, i.e., *the noise in* Eq. (77).

To prove Eq. (84), we start by noticing that $\varphi(x_i)^\top \tilde{\Theta}(t)$ evolves as a Gaussian random variable with time-dependent variance. The idea is to upper bound this variance with that of the auxiliary process

$$\mathrm{d}z_i(t) = \varphi(x_i)^\top \Sigma \, \mathrm{d}B(t),$$

which is obtained by removing the *attractive drift* $[-2\Phi^\top(\Phi\hat{\Theta}(t) - Y)/n]$ from Eq. (77). Intuitively, the removal of the attractive drift increases the variance, as the process becomes less concentrated around the mean. This is formalized in Lemma 3.9, where an application of the *Sudakov-Fernique inequality* gives that

$$\mathbb{E}_B\left[\sup_{t\in[0,\tau]} \left|\varphi(x_i)^\top \tilde{\Theta}(t)\right|\right] \le \mathbb{E}_{z_i}\left[\sup_{t\in[0,\tau]} |z_i(t)|\right]. \qquad [85]$$

We note that the RHS of Eq. (85) is easier to control than the LHS, as $z_i(t)$ is a Wiener process and, therefore, its variance is bounded by $\mathcal{O}\left(\Sigma^2\tau \left\|\varphi(x_i)\right\|_2^2\right)$ via the *reflection principle*. Then, given our hyper-parameter choice in Eq. (75) and the lower bound on ε in Assumption 4, we have that $\Sigma^2\tau \left\|\varphi(x_i)\right\|_2^2 = \mathcal{O}(1)$. To conclude, we exploit the *Borell-TIS inequality* to show that, with high probability,

$$\sup_{t\in[0,\tau]} |\varphi(x_i)^\top \tilde{\Theta}(t)| \le \mathbb{E}_B[\sup_{t\in[0,\tau]} |\varphi(x_i)^\top \tilde{\Theta}(t)|] + \log n,$$

which concludes the argument.

The combination of Lemma 3.1 and 3.2 gives that the event in Eq. (79) happens with high probability, which allows us to study the utility of $\Theta(\tau)$ through the closed form of the OU process $\hat{\Theta}(\tau)$, as $\Theta(\tau) = \hat{\Theta}(\tau)$. This, in turn, boils down to controlling the effects of the noise and of the early stopping that are decoupled via the decomposition $\hat{\Theta}(\tau) = \hat{\theta}(\tau) + \tilde{\Theta}(\tau)$. In fact, on the one hand, $\tilde{\Theta}(\tau)$ is a mean-0 random variable in the probability space of B and it captures the effect of the noise; on the other hand, $\hat{\theta}(\tau)$ is the deterministic component (with respect to B) describing the flow and it captures the effect of the early stopping. We show that both the noise and the early stopping provide negligible damage to utility in the following two lemmas, whose proofs are contained in Section 3-B of the SI.

Lemma 3.3. *Let Assumptions 1, 2 and 4 hold, and let $d = o(p)$. Then, we have*

$$\mathbb{E}_{x\sim\mathcal{P}_X}\left[\left(\varphi(x)^\top\tilde{\Theta}(\tau)\right)^2\right] = \mathcal{O}\left(\frac{d^2\log^{10} n}{n^2}\frac{\log(1/\delta)}{\varepsilon^2}\right) = \tilde{\mathcal{O}}\left(\frac{d^2}{\varepsilon^2 n^2}\right) = o(1), \tag{86}$$

with probability at least $1-2\exp\left(-c\log^2 n\right)$ over V and B, where c is an absolute constant.

Similarly to Lemma 3.2, Lemma 3.3 also uses that $\varphi(x_i)^\top\tilde{\Theta}(\tau)$ is a Gaussian random variable with variance increasing linearly in $\|\varphi(x_i)\|_2^2$, τ, and Σ^2. Then, the claim in Eq. (86) is a consequence of the choice of the hyper-parameters in Eq. (75) and Assumption 4.

Lemma 3.4. *Let Assumptions 1, 2 and 3 hold. Then, we have*

$$\mathbb{E}_{x\sim\mathcal{P}_X}\left[\left(\varphi(x)^\top\left(\hat{\theta}(\tau)-\theta^*\right)\right)^2\right] = \mathcal{O}\left(\frac{d}{n}+\frac{n\log^3 d}{d^{3/2}}\right) = \tilde{\mathcal{O}}\left(\frac{d}{n}+\frac{n}{d^{3/2}}\right) = o(1), \tag{87}$$

with probability at least $1-2\exp\left(-c\log^2 n\right)$ over X and V, where c is an absolute constant.

The idea of the argument is to decompose the LHS of Eq. (87) through two disjoint subspaces, *i.e.*, $\varphi(x)^\top(P_\Lambda+P_\Lambda^\perp)(\hat{\theta}(\tau)-\theta^*)$, where $P_\Lambda\in\mathbb{R}^{p\times p}$ is the projector on the space spanned by the eigenvectors associated with the d largest eigenvalues of $\Phi^\top\Phi$. The rationale is that there is a *spectral gap* between the d-th and the $(d+1)$-th eigenvalue of the kernel K, when sorting them in non-increasing order, see Lemma 4.5. This, in turn, implies that the term $\|P_\Lambda(\hat{\theta}(\tau)-\theta^*)\|_2$ is negligible, since in this subspace $\hat{\theta}(\tau)$ is *already close to convergence*, despite the early stopping. To control the other subspace, we consider the decomposition $\phi(Vx)=\mu_1 Vx+\tilde{\phi}(Vx)$, where μ_1 is the first Hermite coefficient of ϕ. Then, we show that *(i)* $\mathbb{E}_x[(\tilde{\phi}(Vx)^\top P_\Lambda^\perp(\hat{\theta}(\tau)-\theta^*))^2]=\mathcal{O}(d/n+n\log^3 d/d^{3/2})$, exploiting the upper bound on $\|\mathbb{E}_x[\tilde{\phi}(Vx)\tilde{\phi}(Vx)^\top]\|_{\text{op}}$ in Lemma 4.15, and *(ii)* $\mathbb{E}_x[(x^\top V^\top P_\Lambda^\perp(\hat{\theta}(\tau)-\theta^*))^2]=\mathcal{O}\left(d/n+n\log^3 d/d^{3/2}\right)$ exploiting the bound on $\|V^\top P_\Lambda^\perp\Phi^+\|_{\text{op}}$ in Lemma 4.14.

Finally, denoting by $\hat{\mathcal{R}}$ and $\mathcal{R}^*$ the generalization error of $\hat{\Theta}(\tau)$ and θ^* respectively, Lemmas 3.3-3.4 (together with some additional manipulations) guarantee that

$$|\hat{\mathcal{R}}-\mathcal{R}^*| = \tilde{\mathcal{O}}\left(\frac{d}{n\varepsilon}+\sqrt{\frac{d}{n}}+\sqrt{\frac{n}{d^{3/2}}}\right).$$

As $\Theta(\tau)=\hat{\Theta}(\tau)$ (due to Lemmas 3.1-3.2), the result of Theorem 1 follows. The details are deferred to the end of Section 3-B of the SI.

A. Analysis of clipping. This section contains the proofs of Lemmas 3.1 and 3.2, including a number of auxiliary results required by the argument. We make use of the notation introduced in the previous section, including the leave-one-out variables ($\Phi_{-1}\in\mathbb{R}^{(n-1)\times p}$, $Y_{-1}\in\mathbb{R}^{n-1}$, $X_{-1}\in\mathbb{R}^{(n-1)\times d}$, $K_{-1}\in\mathbb{R}^{(n-1)\times(n-1)}$) where the first sample was removed, the OU process $\hat{\Theta}(\tau)=\hat{\theta}(\tau)+\tilde{\Theta}(\tau)$, and we also consider the hyper-parameter choice in Eq. (75). When stating that a random variable Z is sub-Gaussian (sub-exponential), we implicitly mean $\|Z\|_{\psi_2}=\mathcal{O}(1)$ ($\|Z\|_{\psi_1}=\mathcal{O}(1)$), *i.e.*, its sub-Gaussian (sub-exponential) norm does not increase with the scalings of the problem. Given a p.s.d. matrix A, $\lambda_j(A)$ denotes its j-th eigenvalue sorted in non-increasing order ($\lambda_{\max}(A)=\lambda_1(A)\geq\lambda_2(A)\geq\ldots\geq\lambda_s(A)=\lambda_{\min}(A)$). We will indicate with $P_\Lambda\in\mathbb{R}^{p\times p}$ the projector on the space spanned by the eigenvectors associated with the d largest eigenvalues of $\Phi^\top\Phi$. During the proof, we will avoid remarking the implicit conditioning on the high probability event described by Lemma 4.5, necessary for its definition. It is convenient to define the function $\tilde{\phi}(z):=\phi(z)-\mu_1 z$, where μ_1 is the first Hermite coefficient of ϕ. Note that this function is Lipschitz continuous and has the first 3 Hermite coefficients equal to 0, due to Assumption 2. We then define the shorthands $\tilde{\varphi}(x)=\tilde{\phi}(Vx)\in\mathbb{R}^p$, $\tilde{\Phi}\in\mathbb{R}^{n\times p}$ as the matrix containing $\tilde{\varphi}(x_i)\in\mathbb{R}^p$ in its i-th row, and $\tilde{K}=\tilde{\Phi}\tilde{\Phi}^\top\in\mathbb{R}^{n\times n}$.

Lemma 3.5. *Let Assumptions 1 and 2 hold, and let $n=\mathcal{O}\left(\sqrt{p}\right)$, $n\log^3 n=\mathcal{O}\left(d^{3/2}\right)$ and $n=\omega(d)$. Then, we have*

$$\left\|\Phi^+Y-\Phi_{-1}^+Y_{-1}\right\|_2=\mathcal{O}\left(\frac{\log n}{\sqrt{p}}\right), \tag{88}$$

with probability at least $1-2\exp\left(-c\log^2 n\right)$ over V and X, where c is an absolute constant.

Proof. From Lemma 4.5, we have that K is invertible with probability at least $1-2\exp\left(-c_1\log^2 n\right)$ over V and X. Conditioning on such high probability event, we have that also K_{-1} is invertible and, therefore, we can write $\Phi^+=\Phi^\top K^{-1}$ and $\Phi_{-1}^+=\Phi_{-1}^\top K_{-1}^{-1}$. Thus, from the proof of Lemma 4.1 in (30) (see their Equation (44)), we have

$$\Phi^+Y-\Phi_{-1}^+Y_{-1}=\frac{P_{\Phi_{-1}}^\perp\varphi(x_1)}{\left\|P_{\Phi_{-1}}^\perp\varphi(x_1)\right\|_2^2}\left(y_1-\varphi(x_1)^\top\Phi_{-1}^+Y_{-1}\right), \tag{89}$$

where $P_{\Phi_{-1}}$ is the projector over the span of the rows of Φ_{-1}.

To bound the term $\varphi(x_1)^\top \Phi_{-1}^+ Y_{-1}$, we decompose $\varphi(x_1) = \mu_1 V x_1 + \tilde{\varphi}(x_1)$. Thus, an application of the triangle inequality gives

$$\left|\varphi(x_1)^\top \Phi_{-1}^+ Y_{-1}\right| \le \left|\mu_1 x_1^\top V^\top \Phi_{-1}^+ Y_{-1}\right| + \left|\tilde{\varphi}(x_1)^\top \Phi_{-1}^+ Y_{-1}\right|. \quad [90]$$

We bound the two terms in Eq. (90) separately. As for the first term, we have that

$$\left\|V^\top \Phi_{-1}^+ Y_{-1}\right\|_2 \le \left\|V^\top \Phi_{-1}^+\right\|_{\text{op}} \left\|Y_{-1}\right\|_2 = \mathcal{O}(1),$$

where the second step is a consequence of Lemma 4.10, and holds with probability at least $1 - 2\exp\left(-c_2 \log^2 n\right)$ over V and X_{-1}. In fact, notice that considering Φ or Φ_{-1} in Lemma 4.10 does not change the argument, and therefore the final result. Then, conditioning on this high probability event, since x_1 is sub-Gaussian, we have that the first term of Eq. (90) reads

$$\left|\mu_1 x_1^\top V^\top \Phi_{-1}^+ Y_{-1}\right| = \mathcal{O}(\log n), \quad [91]$$

with probability at least $1 - 2\exp\left(-c_3 \log^2 n\right)$ over V and X.

As for the second term in Eq. (90), by triangle inequality, we have that

$$\left\|\left(\Phi_{-1}^+\right)^\top \tilde{\varphi}(x_1)\right\|_2 \le \left\|K_{-1}^{-1} \mathbb{E}_V\left[\Phi_{-1}\tilde{\varphi}(x_1)\right]\right\|_2 + \left\|K_{-1}^{-1}\left(\Phi_{-1}\tilde{\varphi}(x_1) - \mathbb{E}_V\left[\Phi_{-1}\tilde{\varphi}(x_1)\right]\right)\right\|_2. \quad [92]$$

We bound the two terms in Eq. (92) separately. As for the first term, using indices $i \in [n-1]$, we have

$$\mathbb{E}_V\left[\Phi_{-1}\tilde{\varphi}(x_1)\right]_i = p\,\mathbb{E}_v\left[\phi(x_{i+1}^\top v)\tilde{\phi}(x_1^\top v)\right] = p \sum_{l=3}^{+\infty} \mu_l^2 \left(\frac{x_{i+1}^\top x_1}{d}\right)^l, \quad [93]$$

where we use the Hermite decomposition of the functions ϕ and $\tilde{\phi}$. Recall that the first 3 Hermite coefficients of $\tilde{\phi}$ are 0, and the others correspond to the ones of ϕ, which we denote as μ_l. Since x_1 is sub-Gaussian and independent from the x_{i+1}-s, with $\left\|x_{i+1}\right\|_2 = \sqrt{d}$, we have that

$$\max_{i \in [n-1]} \left|x_{i+1}^\top x_1\right| \le \sqrt{d}\log n, \quad [94]$$

with probability at least $1 - 2\exp\left(-c_4 \log^2 n\right)$ over x_1. Thus, conditioning on this high probability event, Eq. (93) gives

$$\left|\mathbb{E}_V\left[\Phi_{-1}\tilde{\varphi}(x_1)\right]_i\right| \le p\left(\frac{\left|x_{i+1}^\top x_1\right|}{d}\right)^3 \sum_{l=3}^{+\infty} \mu_l^2 = \mathcal{O}\left(p\frac{\log^3 n}{d^{3/2}}\right), \quad [95]$$

which implies

$$\left\|K_{-1}^{-1}\mathbb{E}_V\left[\Phi_{-1}\tilde{\varphi}(x_1)\right]\right\|_2 \le \left\|K_{-1}^{-1}\right\|_{\text{op}} \left\|\mathbb{E}_V\left[\Phi_{-1}\tilde{\varphi}(x_1)\right]\right\|_2 = \mathcal{O}\left(\frac{\sqrt{n}\log^3 n}{d^{3/2}}\right), \quad [96]$$

with probability at least $1 - 2\exp\left(-c_5 \log^2 n\right)$ over X and V. Here, the last passage also uses Lemma 4.5, which provides a lower bound on $\lambda_{\min}\left(K_{-1}\right) \ge \lambda_{\min}(K) = \Omega(p)$.

As for the second term of Eq. (92), we have

$$\left[\Phi_{-1}\tilde{\varphi}(x_1)\right]_i - \left[\mathbb{E}_V\left[\Phi_{-1}\tilde{\varphi}(x_1)\right]\right]_i = \sum_{k=1}^{p}\left(\phi(x_{i+1}^\top v_k)\tilde{\phi}(x_1^\top v_k) - \mathbb{E}_{v_k}\left[\phi(x_{i+1}^\top v_k)\tilde{\phi}(x_1^\top v_k)\right]\right). \quad [97]$$

All terms in the previous sum are independent, sub-exponential, mean-0 random variables, since both ϕ and $\tilde{\phi}$ are Lipschitz. Thus, Bernstein inequality (see Theorem 2.8.1 of (31)) gives

$$\left|\left[\Phi_{-1}\tilde{\varphi}(x_1)\right]_i - \mathbb{E}_V\left[\Phi_{-1}\tilde{\varphi}(x_1)\right]_i\right| = \mathcal{O}\left(\log n\sqrt{p}\right), \quad [98]$$

with probability at least $1 - 2\exp\left(-c_6 \log^2 n\right)$ over V. Then, performing a union bound over all the indices $i \in [n-1]$, we obtain

$$\left\|K_{-1}^{-1}\left(\Phi_{-1}\tilde{\varphi}(x_1) - \mathbb{E}_V\left[\Phi_{-1}\tilde{\varphi}(x_1)\right]\right)\right\|_2 = \mathcal{O}\left(\frac{1}{p}\sqrt{np}\log n\right) = \mathcal{O}\left(\sqrt{\frac{n}{p}}\log n\right), \quad [99]$$

with probability at least $1 - 2\exp\left(-c_7 \log^2 n\right)$ over V and X. Then, Eq. (99) and Eq. (96) make Eq. (92) read

$$\left\|\left(\Phi_{-1}^+\right)^\top \tilde{\varphi}(x_1)\right\|_2 = \mathcal{O}\left(\frac{\sqrt{n}\log^3 n}{d^{3/2}} + \sqrt{\frac{n}{p}}\log n\right) = \mathcal{O}\left(\frac{\log n}{\sqrt{n}}\right), \quad [100]$$

with probability at least $1 - 2\exp\left(-c_8 \log^2 n\right)$ over V and X, where the last step is a consequence of $p = \Omega(n^2)$. Plugging Eq. (100) and Eq. (91) in Eq. (90) provides the upper bound

$$\left|\varphi(x_1)^\top \Phi_{-1}^+ Y_{-1}\right| = \mathcal{O}(\log n), \quad [101]$$

which holds with probability at least $1-2\exp\left(-c_9\log^2 n\right)$ over V and X.

To bound the term $\left\|P^{\perp}_{\Phi_{-1}}\varphi(x_1)\right\|_2$ in Eq. (89), we can use Lemma B.1 in (30), which gives

$$\left\|P^{\perp}_{\Phi_{-1}}\varphi(x_1)\right\|_2 \geq \sqrt{\lambda_{\min}\left(K\right)}=\Omega(\sqrt{p}), \tag{102}$$

where the last step is a consequence of Lemma 4.5, and holds with probability at least $1-2\exp\left(-c_{10}\log^2 n\right)$ over V and X. The combination of Eq. (89), Eq. (101) and Eq. (102) readily gives the desired result. □

After performing a union bound on all $i\in[n]$, the result of Lemma 3.5 guarantees that, with high probability,

$$\left\|\Phi^+Y-\Phi^+_{-i}Y_{-i}\right\|_2=\mathcal{O}\left(\frac{\log n}{\sqrt{p}}\right), \qquad \text{for all } i\in[n].$$

This will be key in our later proof of Lemma 3.1, where we will use Lemma 3.5 and the fact that it holds with probability at least $1-2\exp\left(-c\log^2 n\right)$. We note that the proof strategy of Lemma 3.5 is specifically designed to obtain such a probability guarantee, and it differs from previous work, especially in the argument to show

$$\|(\Phi^+_{-i})^\top\tilde{\varphi}(x_i)\|_2=\mathcal{O}\left(\frac{\log n}{\sqrt{n}}\right), \qquad \text{for all } i\in[n],$$

see Eq. (100). Note that, for a fixed i, this can be proved via Markov inequality through an upper bound on the second moment of the LHS of the equation above, which in turn reduces to an upper bound on $\|\mathbb{E}_x[\tilde{\varphi}(x)\tilde{\varphi}(x)^\top]\|_{\text{op}}$. This resembles the strategy followed by (32) (see their Proposition 7.(b)) to prove that this term is negligible when estimating the test loss of the RF model, which is their object of interest. However, this approach would lead to vacuous probability guarantees after performing a union bound on all the training samples, due to the polynomial tail bound given by Markov inequality. To solve the issue, we pursue our desired result via the argument between Eq. (92) and Eq. (96), which leads to

$$\frac{\sqrt{n}\log^3 n}{d^{3/2}}=O\left(\frac{\log n}{\sqrt{n}}\right),$$

and therefore to our assumption $d^{3/2}\gg n$, see Eq. (6). Similarly, in the argument between Eq. (97) and Eq. (99), we upper bound $\|(\Phi^+_{-i})^\top\tilde{\varphi}(x_i)-\mathbb{E}_V\left[(\Phi^+_{-i})^\top\tilde{\varphi}(x_i)\right]\|_2$ via Bernstein inequality on the separate indices of the argument vector, which at the end relies on the bound

$$\sqrt{\frac{n}{p}\log n}=O\left(\frac{\log n}{\sqrt{n}}\right),$$

leading to our assumption $p=\Omega(n^2)$, see Eq. (6).

Lemma 3.6. *Let Assumptions 1 and 2 hold, and let $n=o\left(p/\log^4 p\right)$, $n=\omega\left(d\log^2 d\right)$, and $n=\mathcal{O}\left(d^{3/2}/\log^3 d\right)$. Then, we have that, with probability at least $1-2\exp\left(-c\log^2 n\right)$ over X and V,*

$$\frac{\varphi(x_1)^\top}{\|\varphi(x_1)\|_2}e^{-\frac{2\Phi^\top\Phi}{n}t}\frac{\varphi(x_1)}{\|\varphi(x_1)\|_2}<\frac{\varphi(x_1)^\top}{\|\varphi(x_1)\|_2}e^{-\frac{2\varphi(x_1)\varphi(x_1)^\top}{n}t}\frac{\varphi(x_1)}{\|\varphi(x_1)\|_2}, \tag{103}$$

holds uniformly for all $t\in(0,\tau]$, where c is an absolute constant.

Proof. Let P_Λ be the projector on the space spanned by the eigenvectors associated with the d largest eigenvalues of $\Phi^\top\Phi$, P_0 be the projector on the kernel of $\Phi^\top\Phi$, and $P_\lambda=I-P_\Lambda-P_0$. By definition, we have that $P_0\varphi(x_1)=0$. Thus, since both P_Λ and P_λ are projectors on eigenspaces of $\Phi^\top\Phi$, we can write

$$\begin{aligned}\frac{\varphi(x_1)^\top}{\|\varphi(x_1)\|_2}e^{-\frac{2\Phi^\top\Phi}{n}t}\frac{\varphi(x_1)}{\|\varphi(x_1)\|_2}&=\frac{\varphi(x_1)^\top P_\lambda}{\|\varphi(x_1)\|_2}e^{-\frac{2P_\lambda\Phi^\top\Phi P_\lambda}{n}t}\frac{P_\lambda\varphi(x_1)}{\|\varphi(x_1)\|_2}+\frac{\varphi(x_1)^\top P_\Lambda}{\|\varphi(x_1)\|_2}e^{-\frac{2P_\Lambda\Phi^\top\Phi P_\Lambda}{n}t}\frac{P_\Lambda\varphi(x_1)}{\|\varphi(x_1)\|_2}\\&\leq e^{-\lambda t}\frac{\|P_\lambda\varphi(x_1)\|_2^2}{\|\varphi(x_1)\|_2^2}+e^{-\Lambda t}\frac{\|P_\Lambda\varphi(x_1)\|_2^2}{\|\varphi(x_1)\|_2^2},\end{aligned} \tag{104}$$

where we have defined the shorthands

$$\Lambda=\frac{2\lambda_d(K)}{n}, \qquad \lambda=\frac{2\lambda_{\min}(K)}{n}. \tag{105}$$

Note that the last step of Eq. (104) holds as $e^{-\lambda t}$ and $e^{-\Lambda t}$ are the largest eigenvalues of $e^{-\frac{2\Phi^\top\Phi}{n}t}$ in the subspaces P_λ and P_Λ are respectively projecting on. Furthermore, the RHS of Eq. (103) reads

$$\frac{\varphi(x_1)^\top}{\|\varphi(x_1)\|_2}e^{-\frac{2\varphi(x_1)\varphi(x_1)^\top}{n}t}\frac{\varphi(x_1)}{\|\varphi(x_1)\|_2}=e^{-\frac{2\|\varphi(x_1)\|_2^2}{n}t}. \tag{106}$$

In remaining part of the proof, we use the following inequality

$$1 - \frac{cz}{e} \geq e^{-cz}, \tag{107}$$

which holds for any $c > 0$, and $0 < z \leq 1/c$. We also use that, for all $z > 0$,

$$1 - cz < e^{-cz}. \tag{108}$$

We prove the inequality in Eq. (103) in two disjoint intervals.

1. $\underline{0 < t \leq 1/\Lambda}$:

   In this interval, we can use Eq. (104) and apply Eq. (107) twice (note that $1/\Lambda \leq 1/\lambda$), obtaining

$$\begin{aligned}\frac{\varphi(x_1)^\top}{\left\|\varphi(x_1)\right\|_2} e^{-\frac{2\Phi^\top\Phi}{n}t} \frac{\varphi(x_1)}{\left\|\varphi(x_1)\right\|_2} &\leq e^{-\lambda t} \frac{\left\|P_\lambda \varphi(x_1)\right\|_2^2}{\left\|\varphi(x_1)\right\|_2^2} + e^{-\Lambda t} \frac{\left\|P_\Lambda \varphi(x_1)\right\|_2^2}{\left\|\varphi(x_1)\right\|_2^2} \\ &\leq \left(1 - \frac{\Lambda t}{e}\right) \frac{\left\|P_\Lambda \varphi(x_1)\right\|_2^2}{\left\|\varphi(x_1)\right\|_2^2} + \left(1 - \frac{\lambda t}{e}\right) \frac{\left\|P_\lambda \varphi(x_1)\right\|_2^2}{\left\|\varphi(x_1)\right\|_2^2} \\ &= 1 - \frac{\Lambda t}{e} \frac{\left\|P_\Lambda \varphi(x_1)\right\|_2^2}{\left\|\varphi(x_1)\right\|_2^2} - \frac{\lambda t}{e} \frac{\left\|P_\lambda \varphi(x_1)\right\|_2^2}{\left\|\varphi(x_1)\right\|_2^2}.\end{aligned} \tag{109}$$

   Applying Eq. (108) to Eq. (106) we obtain

$$\frac{\varphi(x_1)^\top}{\left\|\varphi(x_1)\right\|_2} e^{-\frac{2\varphi(x_1)\varphi(x_1)^\top}{n}t} \frac{\varphi(x_1)}{\left\|\varphi(x_1)\right\|_2} > 1 - \frac{2\left\|\varphi(x_1)\right\|_2^2}{n} t. \tag{110}$$

   Then, on this interval, Eq. (109) and Eq. (110) imply that proving the following

$$1 - \frac{\Lambda t}{e} \frac{\left\|P_\Lambda \varphi(x_1)\right\|_2^2}{\left\|\varphi(x_1)\right\|_2^2} - \frac{\lambda t}{e} \frac{\left\|P_\lambda \varphi(x_1)\right\|_2^2}{\left\|\varphi(x_1)\right\|_2^2} \overset{?}{\leq} 1 - \frac{2\left\|\varphi(x_1)\right\|_2^2}{n} t, \tag{111}$$

   is enough to prove the thesis. This, in turn, can be shown proving that

$$\Lambda = \frac{2\lambda_d(K)}{n} \overset{?}{\geq} \frac{2e\left\|\varphi(x_1)\right\|_2^4}{n\left\|P_\Lambda \varphi(x_1)\right\|_2^2}, \tag{112}$$

   where the ? remarks that this inequality still has to be proved.

   Now, by Lemmas 4.5 and 4.12, we jointly have

$$\lambda_d(K) = \Omega\left(\frac{pn}{d}\right) = \Omega(p \log n), \qquad \left\|P_\Lambda \varphi(x_1)\right\|_2^2 = \Omega(p), \tag{113}$$

   with probability at least $1 - 2\exp\left(-c_2 \log^2 n\right)$ over X and V. Then, conditioning on this high probability event, the LHS of Eq. (112) is $\Omega(p \log n\,/n)$, while its RHS is $\mathcal{O}\left(p/n\right)$ (recall that $\left\|\varphi(x_1)\right\|_2^2 = \mathcal{O}\left(p\right)$). Thus, Eq. (112) holds for n sufficiently large, which gives that the desired result holds in the interval $0 < t \leq 1/\Lambda$, with probability at least $1 - 2\exp\left(-c_3 \log^2 n\right)$ over X and V (where c_3 is eventually smaller than c_2, to make this probability being 0 for the n-s that are not large enough).

2. $\underline{1/\Lambda < t \leq \tau}$:

   We have

$$\begin{aligned}\frac{\varphi(x_1)^\top}{\left\|\varphi(x_1)\right\|_2} e^{-\frac{2\Phi^\top\Phi}{n}t} \frac{\varphi(x_1)}{\left\|\varphi(x_1)\right\|_2} &\leq e^{-\lambda t} \frac{\left\|P_\lambda \varphi(x_1)\right\|_2^2}{\left\|\varphi(x_1)\right\|_2^2} + e^{-\Lambda t} \frac{\left\|P_\Lambda \varphi(x_1)\right\|_2^2}{\left\|\varphi(x_1)\right\|_2^2} \\ &\leq \frac{\left\|P_\lambda \varphi(x_1)\right\|_2^2}{\left\|\varphi(x_1)\right\|_2^2} + e^{-1} \frac{\left\|P_\Lambda \varphi(x_1)\right\|_2^2}{\left\|\varphi(x_1)\right\|_2^2} \\ &= 1 - \left(1 - e^{-1}\right) \frac{\left\|P_\Lambda \varphi(x_1)\right\|_2^2}{\left\|\varphi(x_1)\right\|_2^2}.\end{aligned} \tag{114}$$

   Applying Eq. (108) to Eq. (106) we obtain

$$\frac{\varphi(x_1)^\top}{\left\|\varphi(x_1)\right\|_2} e^{-\frac{2\varphi(x_1)\varphi(x_1)^\top}{n}t} \frac{\varphi(x_1)}{\left\|\varphi(x_1)\right\|_2} > 1 - \frac{2\left\|\varphi(x_1)\right\|_2^2}{n} t \geq 1 - \frac{2\left\|\varphi(x_1)\right\|_2^2}{n} \tau. \tag{115}$$

Then, on this interval, Eq. (114) and Eq. (115) imply that proving the following

$$\left(1-e^{-1}\right)\frac{\left\|P_\Lambda\varphi(x_1)\right\|_2^2}{\left\|\varphi(x_1)\right\|_2^2}\overset{?}{\geq}\frac{2\left\|\varphi(x_1)\right\|_2^2}{n}\tau, \tag{116}$$

is enough to prove the thesis. By Lemma 4.12, we have that the LHS of the previous equation is $\Theta(1)$ with probability at least $1-2\exp\left(-c_4\log^2 n\right)$ over X and V. For the RHS, since $\left\|\varphi(x_1)\right\|_2^2=\mathcal{O}\left(p\right)$ with probability at least $1-2\exp\left(-c_5 p\right)$ over V (see the argument carried out in Eq. (295) and Eq. (296)), we have that

$$\frac{2\left\|\varphi(x_1)\right\|_2^2}{n}\tau=\mathcal{O}\left(\frac{p}{n}\frac{d\log^2 n}{p}\right)=o(1). \tag{117}$$

Thus, Eq. (116) holds for n sufficiently large, which gives that the desired result holds in the interval $1/\Lambda<t\leq\tau$, with probability at least $1-2\exp\left(-c_6\log^2 n\right)$ over X and V (where c_6 is set to make this probability being 0 for the n-s that are not large enough).

□

Lemma 3.7. *Let Assumptions 1 and 2 hold, and let $n=o\left(p/\log^4 p\right)$, $n=\omega\left(d\log^2 d\right)$, and $n=\mathcal{O}\left(d^{3/2}/\log^3 d\right)$. Then, we have*

$$\sup_{t\in[0,\tau]}\left|\varphi(x_1)^\top e^{-\frac{2\Phi^\top\Phi}{n}t}\Phi_{-1}^+Y_{-1}\right|\leq 2\sup_{t\in[0,\tau]}\left|\varphi(x_1)^\top e^{-\frac{2\Phi_{-1}^\top\Phi_{-1}}{n}t}\Phi_{-1}^+Y_{-1}\right|, \tag{118}$$

with probability at least $1-2\exp\left(-c\log^2 n\right)$ over X and V, where c is an absolute constant.

Proof. Note that, for any positive number s,

$$e^{-\frac{2\varphi(x_1)\varphi(x_1)^\top}{ns}t}=e^{-\frac{\left\|2\varphi(x_1)\right\|_2^2}{ns}t}\frac{\varphi(x_1)\varphi(x_1)^\top}{\left\|\varphi(x_1)\right\|_2^2}+P^\perp_{\varphi(x_1)}, \tag{119}$$

where $P^\perp_{\varphi(x_1)}\in\mathbb{R}^{p\times p}$ is the orthogonal projector to the space spanned by $\varphi(x_1)$, *i.e.*, $P^\perp_{\varphi(x_1)}=I-\varphi(x_1)\varphi(x_1)^\top/\left\|\varphi(x_1)\right\|_2^2$. Thus, we can write

$$e^{-\frac{2\varphi(x_1)\varphi(x_1)^\top}{ns}t}=I+\alpha(s)\frac{\varphi(x_1)\varphi(x_1)^\top}{\left\|\varphi(x_1)\right\|_2^2}, \tag{120}$$

where we introduced the shorthand

$$-1<\alpha(s)=-\left(1-e^{-\frac{2\left\|\varphi(x_1)\right\|_2^2}{ns}t}\right)<0. \tag{121}$$

Let us also introduce

$$\Pi(s)=\left(e^{-\frac{2\varphi(x_1)\varphi(x_1)^\top}{ns}t}e^{-\frac{2\Phi_{-1}^\top\Phi_{-1}}{ns}t}\right)^s\in\mathbb{R}^{p\times p},\qquad\chi(s)=\left|\varphi(x_1)^\top\Pi(s)\Phi_{-1}^+Y_{-1}\right|\in\mathbb{R}, \tag{122}$$

defined for any s being a positive natural number. As $\varphi(x_1)\varphi(x_1)^\top+\Phi_{-1}^\top\Phi_{-1}=\Phi^\top\Phi$, an application of Lie's product formula gives

$$\lim_{s\to\infty}\Pi(s)=e^{-\frac{2\Phi^\top\Phi}{n}t}, \tag{123}$$

and therefore

$$\lim_{s\to\infty}\chi(s)=\left|\varphi(x_1)^\top e^{-\frac{2\Phi^\top\Phi}{n}t}\Phi_{-1}^+Y_{-1}\right|. \tag{124}$$

Plugging Eq. (120) in the expression of $\Pi(s)$ gives

$$\Pi(s)=\left(\left(I+\alpha(s)\frac{\varphi(x_1)\varphi(x_1)^\top}{\left\|\varphi(x_1)\right\|_2^2}\right)A(s)\right)^s, \tag{125}$$

where we define

$$A(s)=e^{-\frac{2\Phi_{-1}^\top\Phi_{-1}}{ns}t}\in\mathbb{R}^{p\times p}. \tag{126}$$

Note that $\Pi(s)$ can be expanded as

$$\Pi(s)=\left(I+\alpha(s)\frac{\varphi(x_1)\varphi(x_1)^\top}{\left\|\varphi(x_1)\right\|_2^2}\right)\sum_{l=1}^{s-1}\Pi_l(s)A(s)^{s-l}+\left(I+\alpha(s)\frac{\varphi(x_1)\varphi(x_1)^\top}{\left\|\varphi(x_1)\right\|_2^2}\right)A(s)^s, \tag{127}$$

where we define

$$\Pi_l(s) = \left(A(s)\left(I + \alpha(s)\frac{\varphi(x_1)\varphi(x_1)^\top}{\|\varphi(x_1)\|_2^2}\right)\right)^{l-1} A(s)\alpha(s)\frac{\varphi(x_1)\varphi(x_1)^\top}{\|\varphi(x_1)\|_2^2}. \quad [128]$$

In words, $\Pi_l(s)$ includes all the terms where the last term containing $\alpha(s)$ is taken at the $(l+1)$-th factor of Eq. (125). This gives

$$\begin{aligned}
\chi(s) &= \left|\varphi(x_1)^\top \Pi(s)\Phi_{-1}^+ Y_{-1}\right| \\
&= \left|(1+\alpha(s))\,\varphi(x_1)^\top \sum_{l=1}^{s-1}\Pi_l(s)A(s)^{s-l}\Phi_{-1}^+Y_{-1} + (1+\alpha(s))\,\varphi(x_1)^\top A(s)^s\Phi_{-1}^+Y_{-1}\right| \\
&= \left|(1+\alpha(s))\,\alpha(s)\sum_{l=1}^{s-1}\pi_l(s)\varphi(x_1)^\top A(s)^{s-l}\Phi_{-1}^+Y_{-1} + (1+\alpha(s))\,\varphi(x_1)^\top A(s)^s\Phi_{-1}^+Y_{-1}\right| \\
&\le \left|(1+\alpha(s))\,\alpha(s)\sum_{l=1}^{s-1}\pi_l(s)\right| \max_{l\in[s-1]}\left|\varphi(x_1)^\top A(s)^{s-l}\Phi_{-1}^+Y_{-1}\right| + \left|(1+\alpha(s))\,\varphi(x_1)^\top A(s)^s\Phi_{-1}^+Y_{-1}\right|,
\end{aligned} \quad [129]$$

where we introduce the shorthand

$$\begin{aligned}
\pi_l(s) &= \frac{\varphi(x_1)^\top}{\|\varphi(x_1)\|_2}\left(A(s)\left(I+\alpha(s)\frac{\varphi(x_1)\varphi(x_1)^\top}{\|\varphi(x_1)\|_2^2}\right)\right)^{l-1}A(s)\frac{\varphi(x_1)}{\|\varphi(x_1)\|_2} \\
&= \frac{\left(A(s)^{1/2}\varphi(x_1)\right)^\top}{\|\varphi(x_1)\|_2}M_l(s)\frac{A(s)^{1/2}\varphi(x_1)}{\|\varphi(x_1)\|_2},
\end{aligned} \quad [130]$$

and $M_l(s)$ is the p.s.d. matrix defined as

$$M_l(s) = \left(A(s)^{1/2}\left(I+\alpha(s)\frac{\varphi(x_1)\varphi(x_1)^\top}{\|\varphi(x_1)\|_2^2}\right)A(s)^{1/2}\right)^{l-1}. \quad [131]$$

Then, note that Eq. (124) and Eq. (129) give

$$\begin{aligned}
&\left|\varphi(x_1)^\top e^{-\frac{2\Phi^\top\Phi}{n}t}\Phi_{-1}^+Y_{-1}\right| \\
&\le \limsup_{s\to\infty}\left(\left|(1+\alpha(s))\,\alpha(s)\sum_{l=1}^{s-1}\pi_l(s)\right|\max_{l\in[s-1]}\left|\varphi(x_1)^\top e^{-\frac{2\Phi_{-1}^\top\Phi_{-1}}{ns}(s-l)t}\Phi_{-1}^+Y_{-1}\right|\right) \\
&\quad + \limsup_{s\to\infty}\left(|1+\alpha(s)|\left|\varphi(x_1)^\top e^{-\frac{2\Phi_{-1}^\top\Phi_{-1}}{ns}st}\Phi_{-1}^+Y_{-1}\right|\right) \\
&\le \limsup_{s\to\infty}\left|(1+\alpha(s))\,\alpha(s)\sum_{l=1}^{s-1}\pi_l(s)\right|\sup_{t'\in(0,t)}\left|\varphi(x_1)^\top e^{-\frac{2\Phi_{-1}^\top\Phi_{-1}}{n}t'}\Phi_{-1}^+Y_{-1}\right| \\
&\quad + \limsup_{s\to\infty}|1+\alpha(s)|\left|\varphi(x_1)^\top e^{-\frac{2\Phi_{-1}^\top\Phi_{-1}}{n}t}\Phi_{-1}^+Y_{-1}\right| \\
&\le \left(1+\limsup_{s\to\infty}\left|\alpha(s)\sum_{l=1}^{s-1}\pi_l(s)\right|\right)\sup_{t'\in(0,t)}\left|\varphi(x_1)^\top e^{-\frac{2\Phi_{-1}^\top\Phi_{-1}}{n}t'}\Phi_{-1}^+Y_{-1}\right|,
\end{aligned} \quad [132]$$

where in the last line we used $\lim_{s\to\infty}\alpha(s)=0$, which follows from Eq. (121). We will now upper bound the first factor of this last expression, for all $t\in(0,\tau]$, as we will treat the case $t=0$ separately. Note that, following Eq. (130), we have

$$\begin{aligned}
\pi_l(s) &= \frac{\left(A(s)^{1/2}\varphi(x_1)\right)^\top}{\|\varphi(x_1)\|_2}M_l(s)\frac{A(s)^{1/2}\varphi(x_1)}{\|\varphi(x_1)\|_2} \\
&= \frac{\left\|A(s)^{1/2}\varphi(x_1)\right\|_2^2}{\|\varphi(x_1)\|_2^2}\frac{\left(A(s)^{1/2}\varphi(x_1)\right)^\top}{\|A(s)^{1/2}\varphi(x_1)\|_2}M_s(s)^{(l-1)/(s-1)}\frac{A(s)^{1/2}\varphi(x_1)}{\|A(s)^{1/2}\varphi(x_1)\|_2} \\
&\le \frac{\left\|A(s)^{1/2}\varphi(x_1)\right\|_2^2}{\|\varphi(x_1)\|_2^2}\left(\frac{\left(A(s)^{1/2}\varphi(x_1)\right)^\top}{\|A(s)^{1/2}\varphi(x_1)\|_2}M_s(s)\frac{A(s)^{1/2}\varphi(x_1)}{\|A(s)^{1/2}\varphi(x_1)\|_2}\right)^{(l-1)/(s-1)} \\
&=: \frac{\left\|A(s)^{1/2}\varphi(x_1)\right\|_2^2}{\|\varphi(x_1)\|_2^2}\mu(s)^{(l-1)/(s-1)},
\end{aligned} \quad [133]$$

where the second line follows directly from Eq. (131), and the third line is a consequence of Jensen inequality, since $M_s(s)$ is p.s.d. and $l \leq s$.

From Eq. (126), we have that $\lim_{s\to\infty} A(s) = I$. Thus,

$$\begin{aligned}\lim_{s\to\infty} M_s(s) &= \lim_{s\to\infty} \left(A(s)^{1/2} \left(I + \alpha(s) \frac{\varphi(x_1)\varphi(x_1)^\top}{\|\varphi(x_1)\|_2^2} \right) A(s)^{1/2} \right)^{s-1} \\ &= \lim_{s\to\infty} A(s)^{1/2} \Pi(s) \left(\left(I + \alpha(s) \frac{\varphi(x_1)\varphi(x_1)^\top}{\|\varphi(x_1)\|_2^2} \right) A(s) \right)^{-1} A(s)^{1/2} \\ &= \lim_{s\to\infty} \Pi(s),\end{aligned} \tag{134}$$

and

$$\begin{aligned}\lim_{s\to\infty} \mu(s) &= \lim_{s\to\infty} \frac{\left(A(s)^{1/2}\varphi(x_1)\right)^\top}{\|A(s)^{1/2}\varphi(x_1)\|_2} \lim_{s\to\infty} M_s(s) \lim_{s\to\infty} \frac{A(s)^{1/2}\varphi(x_1)}{\|A(s)^{1/2}\varphi(x_1)\|_2} \\ &= \frac{\varphi(x_1)^\top}{\|\varphi(x_1)\|_2} \left(\lim_{s\to\infty} \Pi(s) \right) \frac{\varphi(x_1)}{\|\varphi(x_1)\|_2} \\ &= \frac{\varphi(x_1)^\top}{\|\varphi(x_1)\|_2} e^{-\frac{2\Phi^\top\Phi}{n}t} \frac{\varphi(x_1)}{\|\varphi(x_1)\|_2} \\ &< \frac{\varphi(x_1)^\top}{\|\varphi(x_1)\|_2} e^{-\frac{2\varphi(x_1)\varphi(x_1)^\top}{n}t} \frac{\varphi(x_1)}{\|\varphi(x_1)\|_2} \\ &= e^{-\frac{2\|\varphi(x_1)\|_2^2}{n}t},\end{aligned} \tag{135}$$

where the third line follows from Eq. (123) and Lemma 3.6 guarantees that, with probability at least $1 - 2\exp\left(-c\log^2 n\right)$ over X and V, the fourth line uniformly holds for all $t \in (0, \tau)$. We will condition on this event until the end of the proof.

The previous limit implies that there exists s^* such that, for all $s > s^*$, we have $\mu(s) < e^{-\frac{2\|\varphi(x_1)\|_2^2}{n}t}$. Thus, for such s, we also have

$$0 < \mu(s)^{1/(s-1)} < \mu(s)^{1/s} \leq e^{-\frac{2\|\varphi(x_1)\|_2^2}{ns}t} = 1 + \alpha(s) < 1. \tag{136}$$

Then, for such s, Eq. (133) leads to

$$\begin{aligned}\sum_{l=1}^{s-1} \pi_l(s) &\leq \frac{\left\|A(s)^{1/2}\varphi(x_1)\right\|_2^2}{\|\varphi(x_1)\|_2^2} \sum_{l=1}^{s-1} \mu(s)^{(l-1)/(s-1)} \\ &= \frac{\left\|A(s)^{1/2}\varphi(x_1)\right\|_2^2}{\|\varphi(x_1)\|_2^2} \frac{1-\mu(s)}{1-\mu(s)^{1/(s-1)}} \\ &< \frac{\left\|A(s)^{1/2}\varphi(x_1)\right\|_2^2}{\|\varphi(x_1)\|_2^2} \frac{1-\mu(s)}{-\alpha(s)},\end{aligned} \tag{137}$$

where we solve the geometric series in the second line and use Eq. (136) in the third one. This gives

$$\begin{aligned}&\limsup_{s\to\infty} \left| \alpha(s) \sum_{l=1}^{s-1} \pi_l(s) \right| \\ &\leq \lim_{s\to\infty} \left| \frac{\alpha(s)}{-\alpha(s)} \right| \lim_{s\to\infty} \frac{\left\|A(s)^{1/2}\varphi(x_1)\right\|_2^2}{\|\varphi(x_1)\|_2^2} \lim_{s\to\infty} |1-\mu(s)| \leq 1.\end{aligned} \tag{138}$$

Plugging this last result in Eq. (132), we get

$$\left| \varphi(x_1)^\top e^{-\frac{2\Phi^\top\Phi}{n}t} \Phi_{-1}^+ Y_{-1} \right| \leq 2 \sup_{t'\in(0,t)} \left| \varphi(x_1)^\top e^{-\frac{2\Phi_{-1}^\top\Phi_{-1}}{n}t'} \Phi_{-1}^+ Y_{-1} \right|, \tag{139}$$

which, taking the supremum of $t \in (0, \tau)$, and extending by continuity to $t = 0$, leads to the desired result. □

For the next lemma, it is convenient to define the ϵ-covering number of a separable set T as follows

$$\mathcal{N}(T,\epsilon) := \inf \left\{ |T_0| \; \text{ such that } \; T_0 \subseteq T, \; \text{ and } \; T \subseteq \bigcup_{t_0 \in T_0} \bar{S}(t_0, \epsilon) \right\}, \tag{140}$$

where $|T_0|$ denotes the cardinality of the set T_0, and $\bar{S}(t_0,\epsilon)$ denotes the closed Euclidean ball with center t_0 and radius ϵ. We denote with $\operatorname{diam}(T) = \sup_{u,u' \in T} \left\| u - u' \right\|_2$ the diameter of T.

Lemma 3.8. *Let Assumptions 1 and 2 hold, and let $n = o\left(p/\log^4 p\right)$, $n \log^3 n = \mathcal{O}\left(d^{3/2}\right)$ and $n = \omega(d)$. Let $T \subseteq \mathbb{R}^d$ be the set described by the curve $\gamma : \mathbb{R} \to \mathbb{R}^d$ defined as*

$$\gamma(t) = V^\top e^{-\frac{2\Phi_{-1}^\top \Phi_{-1}}{n} t} \Phi_{-1}^+ Y_{-1}, \tag{141}$$

for $t \in [0,\tau]$. Then, T is separable with respect to the Euclidean norm and, denoting with $\mathcal{N}(T,\epsilon)$ its ϵ-covering number and with $\operatorname{diam}(T)$ its diameter, we jointly have that

$$\int_0^\infty \sqrt{\log \mathcal{N}(T,\epsilon)} \mathrm{d}\epsilon = \mathcal{O}\left(\log n\right), \qquad \operatorname{diam}(T) = \mathcal{O}\left(1\right), \tag{142}$$

with probability at least $1 - 2\exp\left(-c\log^2 n\right)$ over X_{-1} and V, where c is an absolute constant.

Proof. T is described by a continuous function γ applied to $t \in [0,\tau]$. Since the interval $[0,\tau]$ is separable, we also have that T is separable.

Furthermore, note that $e^{-\frac{2\Phi_{-1}^\top \Phi_{-1}}{n} t} \Phi_{-1}^+ = \Phi_{-1}^+ e^{-\frac{2K_{-1}}{n} t}$. Then, for all $t, t' \in [0,\tau]$, we have

$$\left\| \gamma(t) - \gamma(t') \right\|_2 = \left\| V^\top \Phi_{-1}^+ e^{-\frac{2K_{-1}}{n} t} \left(I - e^{-\frac{2K_{-1}}{n}(t'-t)} \right) Y_{-1} \right\|_2. \tag{143}$$

Assuming, without loss of generality, $t' \geq t$, we have that

$$\left\| \gamma(t) - \gamma(t') \right\|_2 \leq \left\| V^\top \Phi_{-1}^+ \right\|_{\mathrm{op}} \left\| Y_{-1} \right\|_2 = \mathcal{O}\left(1\right), \tag{144}$$

where the last step is a consequence of Lemma 4.10 and it holds with probability at least $1 - 2\exp\left(-c_1 \log^2 n\right)$ over X_{-1} and V (note that the argument of Lemma 4.10 goes through equivalently when considering Φ_{-1} instead of Φ). This proves the desired result on the diameter.

Following a similar strategy, we can prove that, for all $t' > t$ s.t. $|t - t'| \leq \Delta$,

$$\begin{aligned} \left\| \gamma(t) - \gamma(t') \right\|_2 &\leq \left\| V^\top \Phi_{-1}^+ \right\|_{\mathrm{op}} \left\| I - e^{-\frac{2K_{-1}}{n}(t'-t)} \right\|_{\mathrm{op}} \left\| Y_{-1} \right\|_2 \\ &\leq \left\| V^\top \Phi_{-1}^+ \right\|_{\mathrm{op}} \left(1 - e^{-\frac{2\lambda_{\max}\left(K_{-1}\right)}{n}(t'-t)} \right) \left\| Y_{-1} \right\|_2 \\ &\leq \left\| V^\top \Phi_{-1}^+ \right\|_{\mathrm{op}} \frac{2\lambda_{\max}\left(K_{-1}\right)}{n} (t'-t) \left\| Y_{-1} \right\|_2 \\ &\leq \left\| V^\top \Phi_{-1}^+ \right\|_{\mathrm{op}} \left\| Y_{-1} \right\|_2 \frac{2\lambda_{\max}\left(K_{-1}\right)}{n} \Delta, \end{aligned} \tag{145}$$

where the second step is a consequence of the fact that $1 - e^{-\frac{2K_{-1}}{n}(t'-t)}$ is a p.s.d. matrix with eigenvalues given by $1 - e^{-\frac{2\lambda_i\left(K_{-1}\right)}{n}(t'-t)}$, and the third step follows from the inequality $z \geq 1 - e^{-z}$, for $z \geq 0$. Since $\left\| \varphi(x_i) \right\|_2^2 = \mathcal{O}\left(p\right)$ jointly for all $i \in [n]$ with probability at least $1 - 2\exp\left(-c_2 p\right)$ over V (see the argument carried out in Eq. (295) and Eq. (296)), we have that $\lambda_{\max}\left(K_{-1}\right) = \left\| \Phi_{-1} \right\|_{\mathrm{op}}^2 \leq \left\| \Phi_{-1} \right\|_F^2 = \mathcal{O}\left(np\right)$. Thus, using again Lemma 4.10, we get that, for every $\Delta > 0$ and for all $t' > t$ s.t. $|t - t'| \leq \Delta$,

$$\left\| \gamma(t) - \gamma(t') \right\|_2 \leq C_1 p \Delta, \tag{146}$$

with probability at least $1 - 2\exp\left(-c_3 \log^2 n\right)$ over X_{-1} and V, where C_1 is an absolute constant independent from the scalings of the problems and from Δ. This means that we can cover T using $\lceil \tau/\Delta \rceil = \left\lceil d \log^2 n / (p\Delta) \right\rceil$ balls with radius $C_1 p \Delta$. Then, setting $\epsilon = C_1 p \Delta$, this implies

$$\mathcal{N}(T,\epsilon) \leq \left\lceil \frac{d \log^2 n}{p} \frac{C_1 p}{\epsilon} \right\rceil = \left\lceil \frac{C_1 d \log^2 n}{\epsilon} \right\rceil. \tag{147}$$

Notice that, for $\epsilon \geq \mathrm{diam}(T)$, we simply have $\mathcal{N}(T,\epsilon)=1$. For smaller values of $\epsilon \leq \mathrm{diam}(T) \leq C_2$, the previous expression reads $\mathcal{N}(T,\epsilon) \leq \frac{C_3 d \log^2 n}{\epsilon}$, where C_3 is another absolute constant set large enough such that $\frac{C_3 d \log^2 n}{\epsilon} \geq 2$, for all $\epsilon \leq C_2$ (this allows to remove the ceiling function), and $C_3 d \log^2 n \geq eC_2$ (which will be used in the next inequality). Then, we have

$$
\begin{aligned}
\int_0^{\infty} \sqrt{\log \mathcal{N}(T,\epsilon)} \mathrm{d}\epsilon &\leq \int_0^{C_2} \sqrt{\log \mathcal{N}(T,\epsilon)} \mathrm{d}\epsilon \\
&\leq \int_0^{C_2} \sqrt{\log\left(\frac{C_3 d \log^2 n}{\epsilon}\right)} \mathrm{d}\epsilon \\
&\leq \int_0^{C_2} \sqrt{\log\left(\frac{eC_2}{\epsilon}\right) + \log\left(\frac{C_3 d \log^2 n}{eC_2}\right)} \mathrm{d}\epsilon \\
&\leq \int_0^{C_2} \sqrt{2\log\left(\frac{eC_2}{\epsilon}\right)} \mathrm{d}\epsilon + \int_0^{C_2} \sqrt{2\log\left(\frac{C_3 d \log^2 n}{eC_2}\right)} \mathrm{d}\epsilon \\
&\leq \sqrt{2} \int_0^{C_2} \log\left(\frac{eC_2}{\epsilon}\right) \mathrm{d}\epsilon + C_2 \sqrt{2\log\left(\frac{C_3 d \log^2 n}{eC_2}\right)} \\
&= \sqrt{2} C_2 + C_4 \log n,
\end{aligned}
\qquad [148]
$$

which concludes the argument. □

Proof of Lemma 3.1. Throughout the proof, we condition on the event occurring with probability at least $1-2\exp\left(-c_1 \log^2 n\right)$ over V and X given by Lemma 4.5. This guarantees that K is invertible and, therefore, that $\Phi^+ = \Phi^\top K^{-1}$, which implies that the LHS of the statement reads $y_i - \varphi(x_i)^\top \hat{\theta}(t) = \varphi(x_i)^\top e^{-\frac{2\Phi^\top \Phi}{n} t} \Phi^+ Y$.

Due to symmetry, we prove the statement for $i=1$, and the thesis will then be true for all $i \in [n]$ by performing a union bound on the n events. A simple application of the triangle inequality gives

$$
\sup_{t\in[0,\tau]} \left| \varphi(x_1)^\top e^{-\frac{2\Phi^\top \Phi}{n} t} \Phi^+ Y \right| \leq \sup_{t\in[0,\tau]} \left| \varphi(x_1)^\top e^{-\frac{2\Phi^\top \Phi}{n} t} \left(\Phi^+ Y - \Phi_{-1}^+ Y_{-1} \right) \right| + \sup_{t\in[0,\tau]} \left| \varphi(x_1)^\top e^{-\frac{2\Phi^\top \Phi}{n} t} \Phi_{-1}^+ Y_{-1} \right|. \qquad [149]
$$

The first term on the RHS can be bounded as follows

$$
\sup_{t\in[0,\tau]} \left| \varphi(x_1)^\top e^{-\frac{2\Phi^\top \Phi}{n} t} \left(\Phi^+ Y - \Phi_{-1}^+ Y_{-1} \right) \right| \leq \left\| \varphi(x_1) \right\|_2 \left\| \Phi^+ Y - \Phi_{-1}^+ Y_{-1} \right\|_2 = \mathcal{O}(\log n), \qquad [150]
$$

where the last step is a consequence of Lemma 3.5 and $\left\| \varphi(x_1) \right\|_2 = \mathcal{O}\left(\sqrt{p}\right)$ (see Eq. (295) and Eq. (296)), and it holds with probability at least $1-2\exp\left(-c_2 \log^2 n\right)$ over V and X. To bound the second term on the RHS of Eq. (149), we first consider the term

$$
\sup_{t\in[0,\tau]} \left| \varphi(x_1)^\top e^{-\frac{2\Phi_{-1}^\top \Phi_{-1}}{n} t} \Phi_{-1}^+ Y_{-1} \right| \leq \sup_{t\in[0,\tau]} \mu_1 \left| x_1^\top \gamma(t) \right| + \sup_{t\in[0,\tau]} \left| \tilde{\varphi}(x_1)^\top \Phi_{-1}^+ e^{-\frac{2K_{-1}}{n} t} Y_{-1} \right|, \qquad [151]
$$

where we define the shorthand $\gamma(t) = V^\top e^{-\frac{2\Phi_{-1}^\top \Phi_{-1}}{n} t} \Phi_{-1}^+ Y_{-1}$. Using Eq. (100), the second term reads

$$
\sup_{t\in[0,\tau]} \left| \tilde{\varphi}(x_1)^\top \Phi_{-1}^+ e^{-\frac{2K_{-1}}{n} t} Y_{-1} \right| \leq \left\| \left(\Phi_{-1}^+ \right)^\top \tilde{\varphi}(x_1) \right\|_2^2 \left\| Y_{-1} \right\|_2^2 = \mathcal{O}(\log n), \qquad [152]
$$

with probability at least $1-2\exp\left(-c_3 \log^2 n\right)$ over X and V. For the first term of Eq. (151), we apply the following decomposition

$$
\sup_{t\in[0,\tau]} \left| x_1^\top \gamma(t) \right| \leq \sup_{t\in[0,\tau]} \left| \left(x_1 - \mathbb{E}_X[x_1] \right)^\top \gamma(t) \right| + \sup_{t\in[0,\tau]} \left| \mathbb{E}_X[x_1]^\top \gamma(t) \right|. \qquad [153]
$$

The second term can be easily bounded as follows

$$
\sup_{t\in[0,\tau]} \left| \mathbb{E}_X[x_1]^\top \gamma(t) \right| \leq \left\| \mathbb{E}_X[x_1] \right\|_2 \sup_{t\in[0,\tau]} \left\| \gamma(t) \right\|_2 \leq \left\| \mathbb{E}_X[x_1] \right\|_2 \left\| V^\top \Phi_{-1}^+ \right\|_{\mathrm{op}} \left\| Y_{-1} \right\|_2 = \mathcal{O}(1), \qquad [154]
$$

where the last step is a consequence of Lemmas 4.1 and 4.10, and it holds with probability at least $1-2\exp\left(-c_4 \log^2 n\right)$ over X_{-1} and V. For the first term of Eq. (153), note that the stochastic process $\left(x_1 - \mathbb{E}_X[x_1] \right)^\top \gamma(t)$ is separable, mean-0, and sub-Gaussian, *i.e.*,

$$
\left\| \left(x_1 - \mathbb{E}[x_1] \right)^\top (u-u') \right\|_{\psi_2} \leq \left\| x^\top (u-u') \right\|_{\psi_2} + \left\| \mathbb{E}[x] \right\|_2 \left\| u-u' \right\|_2 \leq K \left\| u-u' \right\|_2, \qquad [155]
$$

for all $u, u' \in T$ (where we use Lemma 4.1 and the fact that x is sub-Gaussian by Assumption 1). Furthermore, by Lemma 3.8, with probability at least $1 - 2\exp\left(-c_5 \log^2 n\right)$ over X_{-1} and V, we have that, for $t \in [0, \tau]$, $\gamma(t)$ describes a separable set $T \subset \mathbb{R}^d$ such that $\int_0^\infty \sqrt{\log \mathcal{N}(T, \epsilon)}\, \mathrm{d}\epsilon = \mathcal{O}(\log n)$ and $\mathrm{diam}(T) = \mathcal{O}(1)$. Then, conditioning on such event, we can use Dudley's (or chaining tail) inequality (see Theorem 5.29 in (33)), which gives

$$\sup_{t\in[0,\tau]} \left|(x_1 - \mathbb{E}_X[x_1])^\top \gamma(t) - (x_1 - \mathbb{E}_X[x_1])^\top \gamma(0)\right| \leq C_1 \left(\int_0^\infty \sqrt{\log \mathcal{N}(T, \epsilon)}\, \mathrm{d}\epsilon + \mathrm{diam}(T) \log n\right) = \mathcal{O}(\log n), \tag{156}$$

with probability at least $1 - 2\exp\left(-c_6 \log^2 n\right)$ over x_1. Furthermore, we have that

$$\left|(x_1 - \mathbb{E}_X[x_1])^\top \gamma(0)\right| \leq \left|x_1^\top \gamma(0)\right| + \left|\mathbb{E}_X[x_1]^\top \gamma(0)\right| \leq C_2 \log n \left\|\gamma(0)\right\|_2 + \left\|\mathbb{E}_X[x_1]\right\|_2 \left\|\gamma(0)\right\|_2 \leq C_3 \left\|\gamma(0)\right\|_2 \log n = \mathcal{O}(\log n), \tag{157}$$

where the second step holds with probability $1 - 2\exp\left(-c_7 \log^2 n\right)$ over x_1, since it is sub-Gaussian, the third step follows from Lemma 4.1, and the last step follows from the same argument used in Eq. (154), due to Lemma 4.10, and holds with probability $1 - 2\exp\left(-c_8 \log^2 n\right)$ over X_{-1} and V.

Plugging Eq. (156), Eq. (157), and Eq. (154) in Eq. (153) gives $\sup_{t\in[0,\tau]} \left|x_1^\top \gamma(t)\right| = \mathcal{O}(\log n)$ with probability at least $1 - 2\exp\left(-c_9 \log^2 n\right)$ over X and V. Then, this last result, together with Eq. (152) and Eq. (151), leads to

$$\sup_{t\in[0,\tau]} \left|\varphi(x_1)^\top e^{-\frac{2\Phi^\top\Phi}{n}t} \Phi_{-1}^+ Y_{-1}\right| \leq 2 \sup_{t\in[0,\tau]} \left|\varphi(x_1)^\top e^{-\frac{2\Phi_{-1}^\top\Phi_{-1}}{n}t} \Phi_{-1}^+ Y_{-1}\right| = \mathcal{O}(\log n), \tag{158}$$

with probability at least $1 - 2\exp\left(-c_{10} \log^2 n\right)$, where the first step holds because of Lemma 3.7.

Plugging Eq. (150) and Eq. (158) in Eq. (149), and performing a union bound over all the indices $i \in [n]$, leads to the desired result. We finally remark that this result does not require the condition $\log n = \Theta(\log p)$ in Assumption 3. □

Lemma 3.9. *For all $i \in [n]$, we have that*

$$\mathbb{E}_B\left[\left(\varphi(x_i)^\top \tilde{\Theta}(t) - \varphi(x_i)^\top \tilde{\Theta}(s)\right)^2\right] \leq \Sigma^2 \left|t - s\right| \left\|\varphi(x_i)\right\|_2^2. \tag{159}$$

Proof. Let us define the matrix $O \in \mathbb{R}^{p\times p}$ as the orthogonal matrix such that $\Phi^\top\Phi = ODO^\top$, with $D \in \mathbb{R}^{p\times p}$ being a p.s.d. diagonal matrix with eigenvalues sorted in non-increasing way (which we indicate as D_k, $k \in [p]$, for simplicity). Then, we have

$$\mathbb{E}_B\left[\left(\varphi(x_i)^\top \tilde{\Theta}(t) - \varphi(x_i)^\top \tilde{\Theta}(s)\right)^2\right] = \mathbb{E}_B\left[\left(\varphi(x_i)^\top OO^\top \tilde{\Theta}(t) - \varphi(x_i)^\top OO^\top \tilde{\Theta}(s)\right)^2\right]. \tag{160}$$

Note that, multiplying both sides of Eq. (77) by $O^\top$ we get

$$\mathrm{d}\left(O^\top \hat{\Theta}(t)\right) = -\frac{2D}{n}\left(O^\top \hat{\Theta}(t)\right) \mathrm{d}t + \frac{2O^\top\Phi^\top Y}{n} \mathrm{d}t + \Sigma\, \mathrm{d}\left(O^\top B(t)\right). \tag{161}$$

Since D is diagonal, we have that each coordinate of $O^\top\hat{\Theta}(t)$ evolves as an independent, one dimensional, OU process (without drift, eventually), since each coordinate of $O^\top B(t)$ evolves as an independent Wiener processes, for rotational invariance of the Gaussian measure. This implies that, for all $s, t \in [0, \tau]$, and for all $k \neq k'$, we have

$$\mathbb{E}_B\left[[O^\top\tilde{\Theta}(t)]_k [O^\top\tilde{\Theta}(s)]_{k'}\right] = 0. \tag{162}$$

This, together with Eq. (160), gives

$$\begin{aligned}
\mathbb{E}_B\left[\left(\varphi(x_i)^\top \tilde{\Theta}(t) - \varphi(x_i)^\top \tilde{\Theta}(s)\right)^2\right] &= \mathbb{E}_B\left[\left(\sum_{k=1}^p [O^\top\varphi(x_i)]_k [O^\top\tilde{\Theta}(t)]_k - [O^\top\varphi(x_i)]_k [O^\top\tilde{\Theta}(s)]_k\right)^2\right] \\
&= \sum_{k=1}^p \mathbb{E}_B\left[\left([O^\top\varphi(x_i)]_k [O^\top\tilde{\Theta}(t)]_k - [O^\top\varphi(x_i)]_k [O^\top\tilde{\Theta}(s)]_k\right)^2\right] \\
&= \sum_{k=1}^p [O^\top\varphi(x_i)]_k^2\, \mathbb{E}_B\left[\left([O^\top\tilde{\Theta}(t)]_k - [O^\top\tilde{\Theta}(s)]_k\right)^2\right].
\end{aligned} \tag{163}$$

Since $[O^\top\hat{\Theta}(t)]_k$ describes an OU process with drift $\Delta_k = \frac{2D_k}{n}$, standard results on the variance and the auto-correlation of OU processes (see Section 4.4.4 in (29)) read (supposing $\Delta_k \neq 0$)

$$\mathbb{E}_B\left[[O^\top\tilde{\Theta}(t)]_k^2\right] = \frac{\Sigma^2}{2\Delta_k}\left(1 - e^{-2\Delta_k t}\right), \tag{164}$$

and

$$\mathbb{E}_B\left[[O^\top\tilde{\Theta}(t)]_k[O^\top\tilde{\Theta}(s)]_k\right]=\frac{\Sigma^2}{2\Delta_k}\left(e^{-\Delta_k|t-s|}-e^{-\Delta_k(t+s)}\right), \tag{165}$$

which gives

$$\mathbb{E}_B\left[\left([O^\top\tilde{\Theta}(t)]_k-[O^\top\tilde{\Theta}(s)]_k\right)^2\right]=\frac{\Sigma^2}{\Delta_k}\left(1-\frac{e^{-2\Delta_k t}+e^{-2\Delta_k s}}{2}-e^{-\Delta_k|t-s|}+e^{-\Delta_k(t+s)}\right). \tag{166}$$

Then, assuming $t\geq s$ without loss of generality, and introducing the shorthand $\delta=t-s$, we have

$$\begin{aligned}\mathbb{E}_B\left[\left([O^\top\tilde{\Theta}(t)]_k-[O^\top\tilde{\Theta}(s)]_k\right)^2\right]&=\frac{\Sigma^2}{\Delta_k}\left(1-e^{-2\Delta_k s}\left(\frac{1+e^{-2\Delta_k\delta}-2e^{-\Delta_k\delta}}{2}\right)-e^{-\Delta_k\delta}\right)\\&=\frac{\Sigma^2\left(1-e^{-\Delta_k\delta}\right)}{\Delta_k}\left(1-e^{-2\Delta_k s}\left(\frac{1-e^{-\Delta_k\delta}}{2}\right)\right)\\&\leq\frac{\Sigma^2\left(1-e^{-\Delta_k\delta}\right)}{\Delta_k}\\&\leq\Sigma^2\delta=\Sigma^2|t-s|,\end{aligned} \tag{167}$$

where the inequality in the fourth line holds as $1-e^{-x}\leq x$ for all $x\geq 0$. Note that, when $[O^\top\hat{\Theta}(t)]_k$ does not have a drift term ($\Delta_k=0$), it is a Wiener process, which implies that $\mathbb{E}_B\left[\left([O^\top\tilde{\Theta}(t)]_k-[O^\top\tilde{\Theta}(s)]_k\right)^2\right]=\Sigma^2|t-s|$ (see Section 3.8.1 in (29)).

Thus, plugging Eq. (167) in Eq. (163) we get

$$\begin{aligned}\mathbb{E}_B\left[\left(\varphi(x_i)^\top\tilde{\Theta}(t)-\varphi(x_i)^\top\tilde{\Theta}(s)\right)^2\right]&=\sum_{k=1}^{p}[O^\top\varphi(x_i)]_k^2\,\mathbb{E}_B\left[\left([O^\top\tilde{\Theta}(t)]_k-[O^\top\tilde{\Theta}(s)]_k\right)^2\right]\\&\leq\sum_{k=1}^{p}[O^\top\varphi(x_i)]_k^2\,\Sigma^2\,|t-s|\\&=\Sigma^2\,|t-s|\,\|\varphi(x_i)\|_2^2,\end{aligned} \tag{168}$$

which gives the desired result. ☐

Proof of Lemma 3.2. Consider the auxiliary Wiener process $z_i(t)\in\mathbb{R}$ such that (see Section 3.8.1 of (29))

$$\mathbb{E}_{z_i}\left[(z_i(t)-z_i(s))^2\right]=\Sigma^2\,|t-s|\,\|\varphi(x_i)\|_2^2\,. \tag{169}$$

By the reflection principle in standard Wiener processes (see Proposition 3.7 in (34)), we have

$$\mathbb{P}_{z_i}\left(\sup_{t\in[0,\tau]}z_i(t)\geq a\right)=2\,\mathbb{P}_{z_i}\left(z_i(\tau)\geq a\right)\leq 4\exp\left(-\frac{a^2}{\Sigma^2\tau\,\|\varphi(x_i)\|_2^2}\right). \tag{170}$$

By the same argument carried out in Eq. (295) and Eq. (296), we have that $\|\varphi(x_i)\|_2^2=\mathcal{O}(p)$ with probability at least $1-2\exp(-c_1p)$ over V. Thus, there exists a constant C for which we can write

$$\Sigma^2\tau\,\|\varphi(x_i)\|_2^2\leq 4C\frac{d\log^6 n}{n^2}\,\frac{8\log(1/\delta)}{\varepsilon^2}\,\frac{d\log^2 n}{p}\,p=32C\frac{d^2\log^8 n}{n^2}\,\frac{\log(1/\delta)}{\varepsilon^2}=\mathcal{O}(1), \tag{171}$$

where the last step is a consequence of Assumption 4. Then, Eq. (170) reads $\mathbb{P}_{z_i}\left(\sup_{t\in[0,\tau]}z_i(t)\geq a\right)\leq 4\exp\left(-c_2a^2\right)$, which implies

$$\mathbb{E}_{z_i}\left[\sup_{t\in[0,\tau]}|z_i(t)|\right]=\int_0^{+\infty}\mathbb{P}_{z_i}\left(\sup_{t\in[0,\tau]}|z_i(t)|\geq a\right)\mathrm{d}a\leq\int_0^{+\infty}2\mathbb{P}_{z_i}\left(\sup_{t\in[0,\tau]}z_i(t)\geq a\right)\mathrm{d}a\leq C_1, \tag{172}$$

where C_1 is an absolute constant, and the statement holds with probability at least $1-2\exp(-c_1p)$ over V. Note that, by Lemma 3.9, for all $s,t\in[0,\tau]$, we have

$$\mathbb{E}_B\left[\sup_{t\in[0,\tau]}\left|\varphi(x_i)^\top\tilde{\Theta}(t)\right|\right]\leq\Sigma^2\,|t-s|\,\|\varphi(x_i)\|_2^2=\mathbb{E}_{z_i}\left[(z_i(t)-z_i(s))^2\right]. \tag{173}$$

Then, since $\varphi(x_i)^\top\tilde{\Theta}(t)$ and $z_i(t)$ are two mean-0 Gaussian processes, due to Sudakov-Fernique inequality (see Theorem 7.2.11 of (31)), we have that, for all finite subsets $T_0\subset[0,\tau]$,

$$\mathbb{E}_B\left[\sup_{t\in T_0}\left|\varphi(x_i)^\top\tilde{\Theta}(t)\right|\right]\leq\mathbb{E}_{z_i}\left[\sup_{t\in T_0}|z_i(t)|\right]. \tag{174}$$

Differently from Theorem 7.2.11 of (31), we consider the supremum of the absolute value of the processes. The result can in fact be extended to this case by simply looking at both $\varphi(x_i)^\top\tilde\Theta(t)$ and $-\varphi(x_i)^\top\tilde\Theta(t)$. To extend to the previous equation to the full interval $[0,\tau]$, we have that the Kolmogorov continuity theorem (see Theorem 1.8 in (34)) guarantees that $\varphi(x_i)^\top\tilde\Theta(t)$ is almost surely continuous (as its α-moment is bounded by the α-moment of $z_i(t)$ via Eq. (173) since they are both Gaussian processes). Then, in the compact time interval $[0,\tau]$, we have uniform continuity, *i.e.*, for every $h>0$, there exists r, such that, for every $t\in[0,\tau]$, $\left|\varphi(x_i)^\top\tilde\Theta(t+r)-\varphi(x_i)^\top\tilde\Theta(t)\right|<h$, with probability 1 over B. Then, Eq. (174) can be extended by continuity to

$$\mathbb{E}_B\left[\sup_{t\in[0,\tau]}\left|\varphi(x_i)^\top\tilde\Theta(t)\right|\right]\leq\mathbb{E}_{z_i}\left[\sup_{t\in[0,\tau]}\left|z_i(t)\right|\right]\leq C_1, \qquad [175]$$

where the last step is a consequence of Eq. (172).

Note that, by Lemma 3.9, since we have $\varphi(x_i)^\top\tilde\Theta(0)=0$,

$$\sigma_\tau^2:=\sup_{t\in[0,\tau]}\mathbb{E}_B\left[\left(\varphi(x_i)^\top\tilde\Theta(t)\right)^2\right]\leq\Sigma^2\tau\left\|\varphi(x_i)\right\|_2^2=\mathcal{O}(1), \qquad [176]$$

where in the last step we used Eq. (171). Then, the Borell-TIS inequality (see Theorem 2.1.1 of (35)) allows us to write, for all $a>0$,

$$\mathbb{P}_B\left(\sup_{t\in[0,\tau]}\left|\varphi(x_i)^\top\tilde\Theta(t)\right|>\mathbb{E}_B\left[\sup_{t\in[0,\tau]}\left|\varphi(x_i)^\top\tilde\Theta(t)\right|\right]+a\right)<\exp\left(-\frac{a^2}{2\sigma_\tau^2}\right)\leq2\exp\left(-c_3a^2\right). \qquad [177]$$

Thus, setting $a=\log n$, we get

$$\sup_{t\in[0,\tau]}\left|\varphi(x_i)^\top\tilde\Theta(t)\right|\leq\mathbb{E}_B\left[\sup_{t\in[0,\tau]}\left|\varphi(x_i)^\top\tilde\Theta(t)\right|\right]+\log n\leq C_1+\log n=\mathcal{O}(\log n), \qquad [178]$$

with probability at least $1-2\exp\left(-c_3\log^2 n\right)-2\exp\left(-c_1p\right)$ over V and B, where we use Eq. (175) in the second step. Our argument holds for any choice of i. Then, the thesis holds uniformly on every $i\in[n]$ with probability at least $1-2\exp\left(-c_4\log^2 n\right)-2n\exp\left(-c_4p\right)$ over B and V, which provides the desired result. □

B. Analysis of noise and early stopping. This section contains the proofs of Lemmas 3.3 and 3.4, as well as of our main Theorem 1, including a number of auxiliary results required by the argument. We make use of the notation introduced in the previous sections and consider the hyper-parameter choice in Eq. (75).

Proof of Lemma 3.3. We denote with $A\in\mathbb{R}^{p\times p}$ the p.s.d. matrix such that $A^2=A^\top A=\mathbb{E}_x\left[\varphi(x)\varphi(x)^\top\right]$ (the definition is well posed as $\mathbb{E}_x\left[\varphi(x)\varphi(x)^\top\right]$ is p.s.d.). Thus,

$$\left\|A\right\|_F^2=\operatorname{tr}\left(A^\top A\right)=\operatorname{tr}\left(\mathbb{E}_x\left[\varphi(x)\varphi(x)^\top\right]\right)=\mathbb{E}_x\left[\operatorname{tr}\left(\varphi(x)\varphi(x)^\top\right)\right]=\mathbb{E}_x\left[\left\|\varphi(x)\right\|_2^2\right], \qquad [179]$$

where we use the linearity of the trace in the third step, and its cyclic property in the fourth step. Furthermore, we can write

$$\left\|A\right\|_F^2=\mathbb{E}_x\left[\left\|\varphi(x)\right\|_2^2\right]\leq2\left\|\phi(\mathbf{0})\right\|_2^2+2L^2\mathbb{E}_x\left[\left\|Vx\right\|_2^2\right]\leq2\left\|\phi(\mathbf{0})\right\|_2^2+2L^2\left\|V\right\|_{\text{op}}^2\mathbb{E}_x\left[\left\|x\right\|_2^2\right]=\mathcal{O}(p), \qquad [180]$$

where we use that ϕ is Lipschitz in the second step, we denote with $\mathbf{0}\in\mathbb{R}^p$ a vector of zeros, and the last step follows from the bound on $\left\|V\right\|_{\text{op}}$ given by Lemma 4.2, which holds with probability at least $1-2\exp\left(-c_1d\right)$ over V (high probability event over which we will condition until the end of the proof). The introduction of A allows us to rewrite the LHS of Eq. (86) as $\mathbb{E}_x\left[\left(\varphi(x)^\top\tilde\Theta(\tau)\right)^2\right]=\tilde\Theta(\tau)^\top\mathbb{E}_x\left[\varphi(x)\varphi(x)^\top\right]\tilde\Theta(\tau)=\left\|A\tilde\Theta(\tau)\right\|_2^2$, which gives

$$\mathbb{E}_x\left[\left(\varphi(x)^\top\tilde\Theta(\tau)\right)^2\right]=\left\|A\tilde\Theta(\tau)\right\|_2^2=\left\|AOO^\top\tilde\Theta(\tau)\right\|_2^2=\left\|AO\tilde\Delta\rho\right\|_2^2, \qquad [181]$$

where $\tilde\Delta\in\mathbb{R}^{p\times p}$ is a p.s.d. diagonal matrix s.t. $\tilde\Delta^2$ contains $\mathbb{E}_B\left[\left[O^\top\tilde\Theta(\tau)\right]_k^2\right]$ in its k-th entry (O is the same matrix considered in Lemma 3.9), and

$$\rho:=\tilde\Delta^{-1}O^\top\tilde\Theta(\tau)\in\mathbb{R}^p \qquad [182]$$

is a standard Gaussian vector in the probability space of B (since each coordinate of $O^\top\hat\Theta(t)$ evolves as an independent, one dimensional, OU process, by the argument following Eq. (161)). Then, by Theorem 6.3.2 in (31), we have

$$\left\|\left\|AO\tilde\Delta\rho\right\|_2-\left\|AO\tilde\Delta\right\|_F\right\|_{\psi_2}\leq C_1\left\|AO\tilde\Delta\right\|_{\text{op}}\leq C_1\left\|AO\tilde\Delta\right\|_F, \qquad [183]$$

where the sub-Gaussian norm is intended in the probability space of ρ. This can be turned into the tail inequality (see Proposition 2.5.2 in (31))

$$\mathbb{P}_B\left(\left\|AO\tilde\Delta\rho\right\|_2\geq\left\|AO\tilde\Delta\right\|_F+\sqrt{p\Sigma^2\tau}\log n\right)\leq2\exp\left(-c_2\frac{p\Sigma^2\tau\log^2 n}{\left\|AO\tilde\Delta\right\|_F^2}\right). \qquad [184]$$

Since we have $\left\|AO\tilde{\Delta}\right\|_F^2 \leq \|A\|_F^2 \left\|O\tilde{\Delta}\right\|_{\text{op}}^2 \leq \|A\|_F^2 \left\|\tilde{\Delta}\right\|_{\text{op}}^2 \leq C_2 p\Sigma^2\tau$, where the last step follows from Eq. (180) (C_2 is an absolute constant) and the argument in Eq. (167), Eq. (184) gives

$$\left\|AO\tilde{\Delta}\rho\right\|_2^2 = \mathcal{O}\left(p\Sigma^2\tau\log^2 n\right) = \mathcal{O}\left(\frac{d^2\log^{10} n}{n^2}\frac{\log(1/\delta)}{\varepsilon^2}\right) = \tilde{\mathcal{O}}\left(\frac{d^2}{\varepsilon^2 n^2}\right) = o(1), \quad [185]$$

with probability at least $1-2\exp\left(-c_2\log^2 n\right)$ over B, where the last step is a consequence of Assumption 4. Plugging the last equation in Eq. (181) provides the desired result. □

Proof of Lemma 3.4. Consider the projector P_Λ on the space spanned by the eigenvectors associated with the d largest eigenvalues of $\Phi^\top\Phi$. Then, let us decompose the LHS of Eq. (87) as

$$\begin{aligned}
&\mathbb{E}_x\left[\left(\varphi(x)^\top\left(\hat{\theta}(\tau)-\theta^*\right)\right)^2\right] \\
&\leq 2\mathbb{E}_x\left[\left(\varphi(x)^\top P_\Lambda\left(\hat{\theta}(\tau)-\theta^*\right)\right)^2\right] + 2\mathbb{E}_x\left[\left(\varphi(x)^\top P_\Lambda^\perp\left(\hat{\theta}(\tau)-\theta^*\right)\right)^2\right] \\
&\leq 2\mathbb{E}_x\left[\left(\varphi(x)^\top P_\Lambda\left(\hat{\theta}(\tau)-\theta^*\right)\right)^2\right] + 4\mathbb{E}_x\left[\left(\tilde{\varphi}(x)^\top P_\Lambda^\perp\left(\hat{\theta}(\tau)-\theta^*\right)\right)^2\right] + 4\mu_1^2\mathbb{E}_x\left[\left(x^\top V^\top P_\Lambda^\perp\left(\hat{\theta}(\tau)-\theta^*\right)\right)^2\right].
\end{aligned} \quad [186]$$

We now bound the last three terms separately. As usual, we condition on the high probability event given by Lemma 4.5, which guarantees that K is invertible and, therefore, $\hat{\theta}(t) = (1-e^{-\frac{2\Phi^\top\Phi}{n}t})\Phi^+ Y$, with probability at least $1-2\exp\left(-c_2\log^2 n\right)$ over V and X, where $\Phi^+ = \Phi^\top K^{-1}$. For the first term of Eq. (186), exploiting the fact that P_Λ is a projector over an eigenspace of $\Phi^\top\Phi$, we can write

$$P_\Lambda\left(\theta^*-\hat{\theta}(\tau)\right) = P_\Lambda e^{-\frac{2\Phi^\top\Phi}{n}\tau}\Phi^+ Y = P_\Lambda e^{-P_\Lambda\frac{2\Phi^\top\Phi}{n}P_\Lambda\tau}P_\Lambda\Phi^+ Y, \quad [187]$$

where $\left\|P_\Lambda e^{-P_\Lambda\frac{2\Phi^\top\Phi}{n}P_\Lambda\tau}P_\Lambda\right\|_{\text{op}} = e^{-\frac{2\lambda_d(K)}{n}\tau} = \mathcal{O}\left(e^{-c_3\log^2 n}\right)$, where c_3 is a small enough absolute constant, and the last step is a consequence of Lemma 4.5 and $\tau = d\log^2 n/p$, and it holds with probability at least $1-2\exp\left(-c_4\log^2 n\right)$ over V and X. Then, using $ne^{-c_3\log^2 n} = \mathcal{O}(1)$ we have

$$\left\|P_\Lambda\left(\hat{\theta}(\tau)-\theta^*\right)\right\|_2 \leq \left\|P_\Lambda e^{-P_\Lambda\frac{2\Phi^\top\Phi}{n}P_\Lambda\tau}P_\Lambda\right\|_{\text{op}}\left\|\Phi^+\right\|_{\text{op}}\|Y\|_2 = \mathcal{O}\left(\frac{1}{\sqrt{pn}}\right), \quad [188]$$

where the second step holds because of Lemma 4.5. Then, using Eq. (180), the first term in Eq. (186) reads

$$\mathbb{E}_x\left[\left(\varphi(x)^\top P_\Lambda\left(\hat{\theta}(\tau)-\theta^*\right)\right)^2\right] \leq \mathbb{E}_x\left[\|\varphi(x)\|_2^2\right]\left\|P_\Lambda\left(\hat{\theta}(\tau)-\theta^*\right)\right\|_2^2 = \mathcal{O}\left(p\frac{1}{pn}\right) = \mathcal{O}\left(\frac{1}{n}\right), \quad [189]$$

with probability at least $1-2\exp\left(-c_5\log^2 n\right)$ over V and X.

For the second term of Eq. (186), we have

$$\begin{aligned}
\mathbb{E}_x\left[\left(\tilde{\varphi}(x)^\top P_\Lambda^\perp\left(\hat{\theta}(\tau)-\theta^*\right)\right)^2\right] &\leq \left\|\mathbb{E}_x\left[\tilde{\varphi}(x)\tilde{\varphi}(x)^\top\right]\right\|_{\text{op}}\left\|\Phi^+\right\|_{\text{op}}^2\|Y\|_2^2 \\
&= \mathcal{O}\left(\left(\log^4 n + \frac{p\log^3 d}{d^{3/2}}\right)1\frac{1}{p}n\right) \\
&= \mathcal{O}\left(\frac{n}{\sqrt{p}}\frac{\log^4 n}{\sqrt{p}} + \frac{n\log^3 d}{d^{3/2}}\right) \\
&= \mathcal{O}\left(\frac{d}{n} + \frac{n\log^3 d}{d^{3/2}}\right) = o(1).
\end{aligned} \quad [190]$$

Here, the second line follows from Lemmas 4.15 and 4.5, and it holds with probability at least $1-2\exp\left(-c_6\log^2 n\right)$ over X and V.

For the third term of Eq. (186), since x is distributed according to $\mathcal{P}_X$, it is sub-Gaussian and $\|x\|_{\psi_2} = \mathcal{O}(1)$. Then, we can bound its second moment (see (31), Proposition 2.5.2) as follows

$$\begin{aligned}
\mathbb{E}_x\left[\left(x^\top V^\top P_\Lambda^\perp\left(\hat{\theta}(\tau)-\theta^*\right)\right)^2\right] &\leq C_1\left\|V^\top P_\Lambda^\perp\left(\hat{\theta}(\tau)-\theta^*\right)\right\|_2^2 \\
&= C_1\left\|V^\top P_\Lambda^\perp\Phi^+ e^{-\frac{2K}{n}\tau}Y\right\|_2^2 \\
&\leq C_1\left\|V^\top P_\Lambda^\perp\Phi^+\right\|_{\text{op}}^2\|Y\|_2^2 \\
&= \mathcal{O}\left(\frac{d}{n} + \frac{n^2\log^6 d}{d^3}\right) \\
&= \mathcal{O}\left(\frac{d}{n} + \frac{n\log^3 d}{d^{3/2}}\right) = o(1),
\end{aligned} \quad [191]$$

where C_1 is an absolute constant and the fourth line holds with probability at least $1 - 2\exp\left(-c_7 \log^2 n\right)$ over X and V, because of Lemma 4.14. Plugging Eq. (189), Eq. (190), and Eq. (191) in Eq. (186) provides the desired result. □

We would like to remark that a key aspect of the previous argument is the existence of a *spectral gap* between $\lambda_d(K)$ and $\lambda_{d+1}(K)$. This result, proved in Lemma 4.5, critically uses that $\lambda_{\min}\left(X^\top X\right) = \Omega(n)$, which in turn relies on our assumption $\lambda_{\min}\left(\mathbb{E}\left[x^\top x\right]\right) = \Omega(1)$, *i.e.*, that the data covariance is well-conditioned. The spectral gap is what allows us to define a proper early stopping time, which implicitly connects to the spectrum of K via the previous argument. We note that, if $\lambda_{\min}\left(\mathbb{E}\left[x^\top x\right]\right) = o(1)$, then K might lose its spectral gap, a new argument would be required to set the early stopping time, and we eventually expect the final bound on the excess population risk to depend on the condition number of the covariance (as it does also in prior related work (17, 22)).

When discussing Figure 2, we have commented on the regularizing effect of DP-GD which can be connected to the introduction of a ridge penalty. In fact, adding Gaussian noise and early stopping does not allow DP-GD to interpolate the training samples in a similar way to ℓ_2 regularization. The connection between ℓ_2 regularization and early stopping can be made quantitative by inspecting the argument of the previous Lemma 3.4: there, we show that the gradient flow at early stopping is close to the gradient flow at convergence when looking only at the sub-space S_Λ spanned by the eigenvectors of $\Phi^\top\Phi$ associated to the top-d eigenvalues (see Eq. (188)), while the same is not true in the orthogonal sub-space $S_\Lambda^\perp$. When we look at the ridge solution

$$\theta^*_\lambda = \left(\Phi^\top\Phi + \lambda I\right)^{-1}\Phi^\top Y,$$

if λ is chosen such that $\lambda_{d+1}(\Phi^\top\Phi) \ll \lambda \ll \lambda_d(\Phi^\top\Phi)$, we have a similar effect: in the sub-space S_Λ, θ^*_λ is approximately the same as the un-regularized θ^*, while we still avoid over-fitting due to the differences in the orthogonal sub-space $S_\Lambda^\perp$.

Proof of Theorem 1. First, notice that since our model has a quadratic loss, following the first two equations in Assumption 4 we can apply Proposition 2.7. Then, the hyper-parameter Σ in Eq. (75) is sufficiently large to guarantee that the solution $\Theta(\tau)$ of the SDE Eq. (12) at time τ is (ε, δ)-differentially private.

Let us introduce the shorthand $R(x) = \varphi(x)^\top\left(\hat{\Theta}(\tau) - \theta^*\right)$, and denote the generalization error of $\hat{\Theta}(\tau)$ and θ^* by $\hat{\mathcal{R}}$ and $\mathcal{R}^*$, respectively. Then, we have that

$$\begin{aligned}
\left(\hat{\mathcal{R}} - \mathcal{R}^*\right)^2 &= \left(\mathbb{E}_{(x,y)\sim\mathcal{P}_{XY}}\left[\left(\varphi(x)^\top\hat{\Theta}(\tau) - y\right)^2\right] - \mathbb{E}_{(x,y)\sim\mathcal{P}_{XY}}\left[\left(\varphi(x)^\top\theta^* - y\right)^2\right]\right)^2 \\
&= \left(\mathbb{E}_{(x,y)\sim\mathcal{P}_{XY}}\left[R(x)\left(\left(\varphi(x)^\top\hat{\Theta}(\tau) - y\right) + \left(\varphi(x)^\top\theta^* - y\right)\right)\right]\right)^2 \\
&\le \mathbb{E}_x\left[R(x)^2\right]\mathbb{E}_{(x,y)\sim\mathcal{P}_{XY}}\left[\left(\varphi(x)^\top\hat{\Theta}(\tau) + \varphi(x)^\top\theta^* - 2y\right)^2\right] \\
&= \mathbb{E}_x\left[R(x)^2\right]\mathbb{E}_{(x,y)\sim\mathcal{P}_{XY}}\left[\left(R(x) + 2\varphi(x)^\top\theta^* - 2y\right)^2\right] \\
&\le 2\mathbb{E}_x\left[R(x)^2\right]^2 + 2\mathbb{E}_x\left[R(x)^2\right]\mathbb{E}_{(x,y)\sim\mathcal{P}_{XY}}\left[\left(2\varphi(x)^\top\theta^* - 2y\right)^2\right] \\
&\le 2\mathbb{E}_x\left[R(x)^2\right]^2 + 16\mathbb{E}_x\left[R(x)^2\right]\mathbb{E}_x\left[\left(\varphi(x)^\top\theta^*\right)^2\right] + 16\mathbb{E}_x\left[R(x)^2\right]\mathbb{E}_y\left[y^2\right],
\end{aligned} \tag{192}$$

where we factorize the difference of two squares in the second line and use Cauchy-Schwartz inequality in the third line. By Lemmas 3.3 and 3.4, we have that

$$\begin{aligned}
\mathbb{E}_x\left[R(x)^2\right] &= \mathbb{E}_x\left[\left(\varphi(x)^\top\left(\hat{\theta}(\tau) + \tilde{\Theta}(\tau) - \theta^*\right)\right)^2\right] \\
&\le 2\mathbb{E}_x\left[\left(\varphi(x)^\top\tilde{\Theta}(\tau)\right)^2\right] + 2\mathbb{E}_x\left[\left(\varphi(x)^\top\left(\hat{\theta}(\tau) - \theta^*\right)\right)^2\right] \\
&= \mathcal{O}\left(\frac{d^2\log^{10} n}{n^2}\frac{\log(1/\delta)}{\varepsilon^2} + \frac{d}{n} + \frac{n\log^3 d}{d^{3/2}}\right) = o\left(1\right),
\end{aligned} \tag{193}$$

with probability at least $1 - 2\exp\left(-c_1\log^2 n\right)$ over X, V and B. Furthermore, by Lemma 4.16, we also have that $\mathbb{E}_x\left[\left(\varphi(x)^\top\theta^*\right)^2\right] = \mathcal{O}\left(1\right)$ with probability at least $1 - 2\exp\left(-c_2\log^2 n\right)$ over X and V. Since also $\mathbb{E}_y\left[y^2\right] = \mathcal{O}\left(1\right)$ by Assumption 1, plugging Eq. (193) in Eq. (192) gives

$$\left(\hat{\mathcal{R}} - \mathcal{R}^*\right)^2 = \mathcal{O}\left(\frac{d^2\log^{10} n}{n^2}\frac{\log(1/\delta)}{\varepsilon^2} + \frac{d}{n} + \frac{n\log^3 d}{d^{3/2}}\right) = o(1), \tag{194}$$

with probability at least $1 - 2\exp\left(-c_3\log^2 n\right)$ over X, V and B. Then, since $\sqrt{a+b+c} \le \sqrt{a} + \sqrt{b} + \sqrt{c}$, we have that, with

this same probability, we have

$$\begin{aligned}\left|\hat{\mathcal{R}}-\mathcal{R}^*\right| &= \mathcal{O}\left(\frac{d}{n\varepsilon}\ \log^5 n\sqrt{\log(1/\delta)}+\sqrt{\frac{d}{n}}+\sqrt{\frac{n\log^3 d}{d^{3/2}}}\right)\\ &= \tilde{\mathcal{O}}\left(\frac{d}{n\varepsilon}+\sqrt{\frac{d}{n}}+\sqrt{\frac{n}{d^{3/2}}}\right)=o(1).\end{aligned} \tag{195}$$

To conclude the argument, we can now use Lemmas 3.1 and 3.2, which guarantee that, jointly for all $i\in[n]$, we have that

$$\sup_{t\in[0,\tau]}\left(\left|\varphi(x_i)^\top\hat{\Theta}(t)\right|+\left|\varphi(x_i)^\top\hat{\theta}(t)-y_i\right|\right)\leq C_1\log n, \tag{196}$$

with probability at least $1-2\exp(-c_4\log^2 n)$ over X, V, and B, for some absolute constant C_1. Since we have, with probability at least $1-2\exp\left(-c_5 p\right)$ over V, that $\left\|\varphi(x_i)\right\|_2\leq C_2\sqrt{p}$ for some absolute constant C_2 (see the argument carried out in Eq. (295) and Eq. (296)) jointly for every $i\in[n]$, the previous equation also reads

$$\begin{aligned}\sup_{t\in[0,\tau]}\left(\left|\varphi(x_i)^\top\hat{\Theta}(t)\right|+\left|\varphi(x_i)^\top\hat{\theta}(t)-y_i\right|\right)&\leq C_1\log n\leq\frac{C_2\sqrt{p}}{\left\|\varphi(x_i)\right\|_2}C_1\log n\\ &\leq\frac{\sqrt{p}\log^2 n}{2\left\|\varphi(x_i)\right\|_2}=\frac{C_{\text{clip}}}{2\left\|\varphi(x_i)\right\|_2}\end{aligned} \tag{197}$$

with probability at least $1-2\exp(-c_6\log^2 n)$ over X, V, and B, where c_6 may depend on C_1 and C_2 due to the step in the second line. Hence, due to the argument between Eq. (78) and Eq. (82), we have that, with the same probability,

$$\Theta(\tau)=\hat{\Theta}(\tau), \tag{198}$$

which in turn implies $\hat{\mathcal{R}}=\mathcal{R}$, where $\mathcal{R}$ is the generalization error of $\Theta(\tau)$. This, together with Eq. (195), concludes the proof. ☐

4. Auxiliary Lemmas

In this section we will consider further standard notation. We will denote by $\mathbf{1}:\mathbb{R}\to\{0,1\}$ the indicator function, *i.e.* $\mathbf{1}(z)=1$ if $z>0$, and 0 otherwise. Given a matrix A, we indicate with $\sigma_{\min}(A)=\sqrt{\lambda_{\min}\left(A^\top A\right)}$ its smallest singular value. Given a p.s.d. matrix A, $\lambda_j(A)$ denotes its j-th eigenvalue sorted in non-increasing order ($\lambda_{\max}\left(A\right)=\lambda_1(A)\geq\lambda_2(A)\geq\ldots\geq\lambda_s(A)=\lambda_{\min}\left(A\right)$). Given two matrices $A\in\mathbb{R}^{s\times s_1}$ and $B\in\mathbb{R}^{s\times s_2}$, we denote by $A*B=\left[(A_{1:}\otimes B_{1:}),\ldots,(A_{s:}\otimes B_{s:})\right]^\top\in\mathbb{R}^{s\times s_1 s_2}$ their row-wise Kronecker product (also known as Khatri-Rao product), and $A_{j:}$ denotes the j-th row of A. Given a natural number l, we denote $A^{*l}=A*A^{*(l-1)}$, with $A^{*1}=A$. We remark that $\left[\left(A^{*l}\right)\left(A^{*l}\right)^\top\right]_{ij}=\left(\left[AA^\top\right]_{ij}\right)^l$. Given $A\in\mathbb{R}^{s\times s}$ and $B\in\mathbb{R}^{s\times s}$, we use the notation $A\preceq B$ ($B\succeq A$) to indicate that $B-A$ is p.s.d. As in Section 3 of the SI, we will indicate with $P_\Lambda\in\mathbb{R}^{p\times p}$ the projector on the space spanned by the eigenvectors associated with the d largest eigenvalues of $\Phi^\top\Phi$. In the following Lemmas, we will avoid remarking the implicit conditioning on the high probability event described by Lemma 4.5, necessary for its definition.

Lemma 4.1. *Let Assumption 1 hold, and let $x\sim\mathcal{P}_X$. Then, we have that*

$$\left\|\mathbb{E}[x]\right\|_2=\mathcal{O}\left(1\right), \tag{199}$$

and

$$\left\|\mathbb{E}[xx^\top]\right\|_{\text{op}}=\mathcal{O}\left(1\right). \tag{200}$$

Proof. The first statement is a direct consequence of x being sub-Gaussian. In fact, this implies

$$\mathbb{E}\left[\left(x^\top\frac{\mathbb{E}[x]}{\left\|\mathbb{E}[x]\right\|_2}\right)^2\right]=\mathcal{O}\left(1\right), \tag{201}$$

as the second moment of sub-Gaussian random variables is bounded. Thus, we have

$$\left\|\mathbb{E}[x]\right\|_2^2=\mathbb{E}\left[x^\top\frac{\mathbb{E}[x]}{\left\|\mathbb{E}[x]\right\|_2}\right]^2\leq\mathbb{E}\left[\left(x^\top\frac{\mathbb{E}[x]}{\left\|\mathbb{E}[x]\right\|_2}\right)^2\right]=\mathcal{O}\left(1\right), \tag{202}$$

which provides the desired result.

For the second statement, we have, for every $u \in \mathbb{R}^d$ such that $\|u\|_2 = 1$,

$$u^\top \mathbb{E}\left[xx^\top\right] u = \mathbb{E}\left[\left(x^\top u\right)^2\right] = \mathcal{O}(1), \tag{203}$$

where the second step holds as the second moment of sub-Gaussian random variables is bounded. Since $\mathbb{E}\left[xx^\top\right]$ is p.s.d., its operator norm is $\sup_{\|u\|_2=1} u^\top \mathbb{E}\left[xx^\top\right] u$, and the second statement readily follows. □

Lemma 4.2. *Let Assumption 1 hold, and let $d = o(n)$ and $d = o(p)$. Then, we have that*

$$\lambda_{\min}\left(X^\top X\right) = \Theta(n), \tag{204}$$

and

$$\|X\|_{\text{op}} = \mathcal{O}\left(\sqrt{n}\right), \tag{205}$$

with probability at least $1 - 2\exp(-cd)$ over X, where c is an absolute constant.

Also, we have that

$$\lambda_{\min}\left(V^\top V\right) = \Theta\left(\frac{p}{d}\right), \tag{206}$$

and

$$\|V\|_{\text{op}} = \mathcal{O}\left(\sqrt{\frac{p}{d}}\right), \tag{207}$$

with probability at least $1 - 2\exp(-cd)$ over V.

Proof. Notice that $X \in \mathbb{R}^{n\times d}$ is a matrix with i.i.d. sub-Gaussian rows with second moment matrix $\Psi = \mathbb{E}_{x\sim\mathcal{P}_X}\left[xx^\top\right] \in \mathbb{R}^{d\times d}$. Thus, by Remark 5.40 in (36), we have that

$$\left\|X^\top X - n\Psi\right\|_{\text{op}} = \mathcal{O}\left(n\frac{d}{n}\right) = o(n), \tag{208}$$

with probability at least $1 - 2\exp(-c_2 d)$. Then, conditioning on this high probability event, by Weyl's inequality, we have

$$\lambda_{\min}\left(X^\top X\right) \le \left\|X^\top X\right\|_{\text{op}} \le \|n\Psi\|_{\text{op}} + \left\|X^\top X - n\Psi\right\|_{\text{op}} = \mathcal{O}(n), \tag{209}$$

where the last step is a consequence of Lemma 4.1. This proves the upper bound in the first statement as well as the second statement. Furthermore, again by Weyl's inequality, we have

$$\lambda_{\min}\left(X^\top X\right) \ge \lambda_{\min}(n\Psi) - \left\|X^\top X - n\Psi\right\|_{\text{op}} = \Omega(n), \tag{210}$$

where the last step is a consequence of $\lambda_{\min}(\Psi) = \Omega(1)$, true by Assumption 1. Merging this with Eq. (209) concludes the proof of the first statement.

The third statement can be proven in the same exact way, considering $\sqrt{d}V \in \mathbb{R}^{p\times d}$, a matrix with i.i.d. standard Gaussian (and therefore sub-Gaussian) rows with second moment matrix equal to the identity $I \in \mathbb{R}^{d\times d}$. Finally, the fourth statement is a consequence of Theorem 4.4.5 of (31), and the scaling $d = o(p)$, and holds with probability $1 - 2\exp(-c_3 d)$. This concludes the proof and provides the desired results. □

Lemma 4.3. *Let Assumptions 1 and 2 hold, and let $n\log^3 n = o\left(d^{3/2}\right)$ and $n = \omega(d)$. Then with probability at least $1 - 2\exp\left(-c\log^2 n\right)$ over X, all the following hold*

$$\lambda_d(\mathbb{E}_V[K]) = \Omega\left(\frac{pn}{d}\right), \tag{211}$$

$$\lambda_{d+1}(\mathbb{E}_V[K]) = \mathcal{O}(p), \tag{212}$$

$$\lambda_n(\mathbb{E}_V[K]) = \lambda_{\min}(\mathbb{E}_V[K]) = \Omega(p), \tag{213}$$

where c is an absolute constant.

Proof. As $K_{ij} = \varphi(x_i)^\top \varphi(x_j)$, we have

$$\mathbb{E}_V[K]_{ij} = \sum_{k=1}^{p} \mathbb{E}_{v_k}[\phi(v_k^\top x_i)\phi(v_k^\top x_j)] = p\,\mathbb{E}_v[\phi(v^\top x_i)\phi(v^\top x_j)], \tag{214}$$

where in the last step we exploited that the v_k-s are identically distributed, and introduced the shorthand v to indicate a random variable distributed as v_1. Since $\|x_i\| = \sqrt{d}$ for all $i \in [n]$, and $v \sim \mathcal{N}(0, 1/d)$, we have that $\rho_1 := v^\top x_i$ and $\rho_2 := v^\top x_j$ are two standard Gaussian variable with correlation $x_i^\top x_j / d$. Thus, exploiting the Hermite expansion of ϕ, we can write

$$\mathbb{E}_V[K]_{ij} = p \sum_{l=0}^{+\infty} \mu_l^2 \frac{\left(x_i^\top x_j\right)^l}{d^l} = p \sum_{l=0}^{\infty} \mu_l^2 \frac{\left[\left(X^{*l}\right)\left(X^{*l}\right)^\top\right]_{ij}}{d^l}, \tag{215}$$

where μ_l is the l-th Hermite coefficient of ϕ. Since ϕ is such that $\mu_l = 0$ for $l = \{0, 2\}$, $\mu_1 \neq 0$, and there exists $l \geq 3$ such that $\mu_l \neq 0$ (since ϕ is non-linear), we can write the previous sum with the two non-0 terms

$$\mathbb{E}_V[K]_{ij} = \frac{\mu_1^2 p}{d} X X^\top + p \sum_{l=3}^{+\infty} \mu_l^2 \frac{\left[\left(X^{*l}\right)\left(X^{*l}\right)^\top\right]_{ij}}{d^l}. \tag{216}$$

We analyze the terms of the sum separately. By Lemma 4.2 we have that, with probability at least $1 - 2\exp(-c_1 d)$ over X,

$$\lambda_{\min}\left(X^\top X\right) = \Theta(n). \tag{217}$$

As $X \in \mathbb{R}^{n \times d}$, with $d < n$, this means that (conditioning on this high probability event) $XX^\top$ is a matrix with rank equal to d, and that its first d eigenvalues (when sorted in non-increasing order) are all $\Omega(n)$. This implies that

$$M_1 := \frac{\mu_1^2 p}{d} X X^\top \tag{218}$$

has the first d eigenvalues of order $\Omega(pn/d)$, and the remaining $n - d$ equal to 0.

We now consider the second term of Eq. (216):

$$M_2 = p \sum_{l=3}^{\infty} \mu_l^2 \frac{\left[\left(X^{*l}\right)\left(X^{*l}\right)^\top\right]_{ij}}{d^l}. \tag{219}$$

Let us define

$$\tilde{M}_2 = p \left(\sum_{l=3}^{\infty} \mu_l^2\right) I = C_1 p I, \tag{220}$$

corresponding to the diagonal elements of M_2, where C_1 is a natural constant depending only on ϕ. Notice that C is positive, as ϕ is non-linear. For $i \neq j$, since x_i is sub-Gaussian and independent from x_j, we have

$$\mathbb{P}\left(\left|x_i^\top x_j\right| > \log n \sqrt{d}\right) < 2\exp\left(-c_2 \log^2 n\right). \tag{221}$$

Performing a union bound we also get

$$\mathbb{P}\left(\max_{i,j}\left|x_i^\top x_j\right| > \log n \sqrt{d}\right) < 2n^2 \exp\left(-c_2 \log^2 n\right) < 2\exp\left(-c_3 \log^2 n\right). \tag{222}$$

This implies that

$$\begin{aligned}
\left\|M_2 - \tilde{M}_2\right\|_{\text{op}} &\leq \left\|M_2 - \tilde{M}_2\right\|_F \\
&= p \left\| \sum_{l=3}^{\infty} \mu_l^2 \left(\frac{\left(X^{*l}\right)\left(X^{*l}\right)^\top}{d^l} - I\right)\right\|_F \\
&\leq p \sum_{l=3}^{\infty} \mu_l^2 \left\| \frac{\left(X^{*l}\right)\left(X^{*l}\right)^\top}{d^l} - I\right\|_F \\
&\leq p \sum_{l=3}^{\infty} \mu_l^2 \sqrt{n^2 \left(\frac{\max_{i,j}\left|x_i^\top x_j\right|^l}{d^l}\right)^2} \\
&\leq p \sqrt{n^2 \left(\frac{\max_{i,j}\left|x_i^\top x_j\right|^3}{d^3}\right)^2} \sum_{l=3}^{\infty} \mu_l^2 \\
&= \mathcal{O}\left(p \frac{n \log^3 n}{d^{3/2}}\right) \\
&= o(p).
\end{aligned} \tag{223}$$

Merging Eq. (220) and Eq. (223) together, a standard application of Weyl's inequality gives

$$\lambda_{\max}\left(M_2\right)=\Theta(p), \qquad \lambda_{\min}\left(M_2\right)=\Theta(p), \tag{224}$$

with probability at least $1-2\exp\left(-c_3\log^2 n\right)$.

Thus, applying Weyl's inequality, we have that

$$\begin{aligned}
\lambda_i\left(M_1+M_2\right) &\geq \lambda_i\left(M_1\right)-\lambda_{\max}\left(M_2\right)=\Omega(pn/d), \qquad \text{for } i\in[d],\\
\lambda_i\left(M_1+M_2\right) &\leq \lambda_i\left(M_1\right)+\lambda_{\max}\left(M_2\right)=\mathcal{O}\left(p\right), \qquad \text{for } d<i\leq n,
\end{aligned} \tag{225}$$

where in both lines we used our argument in Eq. (218) and Eq. (224). Since $\mathbb{E}_V[K]=M_1+M_2$, we have proved that the spectrum of $\mathbb{E}_V[K]$ has a gap, as

$$\lambda_d\left(\mathbb{E}_V[K]\right)=\Omega\left(\frac{pn}{d}\right)=\omega(p), \tag{226}$$

and

$$\lambda_{d+1}\left(\mathbb{E}_V[K]\right)=\mathcal{O}\left(p\right). \tag{227}$$

This proves Eq. (211) and Eq. (212). To show Eq. (213), it suffices to note that

$$\lambda_{\min}\left(\mathbb{E}_V[K]\right)\geq\lambda_{\min}\left(M_2\right)=\Theta(p), \tag{228}$$

where the first step is true since M_1 is p.s.d., and the second step follows from Eq. (224). □

Lemma 4.4. *Let Assumptions 1 and 2 hold, and let $n=\mathcal{O}\left(p/\log^4 p\right)$, $n\log^3 n=o\left(d^{3/2}\right)$ and $n=\omega(d)$. Then, we have that*

$$\left\|\mathbb{E}_V[K]^{-1/2}\left(K-\mathbb{E}_V[K]\right)\mathbb{E}_V[K]^{-1/2}\right\|_{\text{op}}=\mathcal{O}\left(\sqrt{\frac{n}{p}}\log n\log p\right), \tag{229}$$

with probability at least $1-2\exp\left(-c\log^2 n\right)$ over X and V, where c is an absolute constant.

Proof. Consider the truncated function

$$\bar\phi(z):=\phi(z)\mathbf{1}\left(|z|\leq\frac{\log p}{L}\right), \tag{230}$$

where L is the Lipschitz constant of ϕ. Define also the truncated kernel $\bar K$ accordingly

$$\bar K_{ij}:=\bar\phi(Vx_i)^\top\bar\phi(Vx_j). \tag{231}$$

We now compare $\mathbb{E}_V\left[\bar K\right]$ and $\mathbb{E}_V[K]$. Let $v\sim\mathcal{N}(0,I/d)$ and define the random variable

$$E_{ij}:=\mathbf{1}\left(|v^\top x_i|>\frac{\log p}{L} \quad\text{or}\quad |v^\top x_j|>\frac{\log p}{L}\right). \tag{232}$$

As E_{ij} is an indicator, $E_{ij}=E_{ij}^2$ and

$$\mathbb{P}_V(E_{ij}=1)\leq\mathbb{P}_V\left(|v^\top x_i|>\frac{\log p}{L}\right)+\mathbb{P}_V\left(|v^\top x_j|>\frac{\log p}{L}\right)\leq 2\exp\left(-c_1\log^2 p\right), \tag{233}$$

where the second step holds as $v^\top x_j\sim\mathcal{N}(0,1)$ and $v^\top x_i\sim\mathcal{N}(0,1)$ in the probability space of V. Thus, a union bound over i and j gives

$$K=\bar K \tag{234}$$

with probability at least $1-2n^2\exp\left(-c_1\log^2 p\right)\geq 1-2\exp\left(-c_2\log^2 n\right)$ over V. Furthermore, we can write

$$\begin{aligned}
\left|\mathbb{E}_V\left[\bar K_{ij}\right]-\mathbb{E}_V\left[K_{ij}\right]\right| &= p\left|\mathbb{E}_v\left[\phi(v^\top x_i)\phi(v^\top x_j)\,E_{ij}\right]\right|\\
&\leq p\,\mathbb{E}_v\left[\left(\phi(v^\top x_i)\phi(v^\top x_j)\right)^2\right]^{1/2}\mathbb{E}_v\left[E_{ij}^2\right]^{1/2}\\
&\leq p\,\mathbb{E}_v\left[\left(\phi(v^\top x_i)\right)^4\right]^{1/2}\mathbb{P}_V(E_{ij}=1)^{1/2}\\
&\leq 2p\,C_1^{1/2}\exp\left(-\frac{c_1\log^2 p}{2}\right)\\
&\leq 2p\exp\left(-c_3\log^2 p\right).
\end{aligned} \tag{235}$$

Here, the second and third line follow from Cauchy-Schwartz inequality; the fourth line is a consequence of Eq. (233) and $\phi(v^\top x_i)$ being a sub-Gaussian random variable ($\phi(z)$ is Lipschitz) and thus with bounded fourth moment (see Equation 2.11 in (31)). This result holds for any $i, j \in [n]$, and therefore implies

$$\begin{aligned}\left\|\mathbb{E}_V\left[\bar{K}\right]-\mathbb{E}_V[K]\right\|_{\mathrm{op}} &\leq \left\|\mathbb{E}_V\left[\bar{K}\right]-\mathbb{E}_V[K]\right\|_F \\ &\leq 2pn\exp\left(-c_3\log^2 p\right) \\ &\leq 2\exp\left(-c_4\log^2 p\right).\end{aligned} \quad [236]$$

Then, by Weyl's inequality, we have

$$\lambda_{\min}\left(\mathbb{E}_V\left[\bar{K}\right]\right) \geq \lambda_{\min}\left(\mathbb{E}_V\left[K\right]\right) - \left\|\mathbb{E}_V\left[\bar{K}\right]-\mathbb{E}_V[K]\right\|_{\mathrm{op}} = \Omega(p), \quad [237]$$

where the last step follows from Lemma 4.3 and it holds with probability at least $1-2\exp\left(-c_5\log^2 n\right)$ over X.

We now prove the bound in Eq. (229) on the truncated kernel $\bar{K}$. Let us then define the matrix $H_k \in \mathbb{R}^{n\times n}$ as

$$H_k = \mathbb{E}_V[\bar{K}]^{-1/2}\bar{\phi}(Xv_k)\bar{\phi}(Xv_k)^\top\mathbb{E}_V[\bar{K}]^{-1/2}. \quad [238]$$

This definition requires $\mathbb{E}_V[\bar{K}]$ to be invertible, and it is therefore conditioned on the event described by Eq. (237). We will condition on this high probability event over X until the end of the proof. Then, we have

$$\mathbb{E}_V[\bar{K}]^{-1/2}\left(\bar{K}-\mathbb{E}_V[\bar{K}]\right)\mathbb{E}_V[\bar{K}]^{-1/2} = \sum_{k=1}^{p} H_k - \mathbb{E}_V\left[H_k\right]. \quad [239]$$

Note that there exists a natural constant C such that

$$\begin{aligned}\sup_{v_k}\left\|H_k-\mathbb{E}_V[H_k]\right\|_{\mathrm{op}} \leq 2\sup_{v_k}\left\|H_k\right\|_{\mathrm{op}} &\leq 2\left\|\mathbb{E}_V[\bar{K}]^{-1/2}\right\|_{\mathrm{op}}^2 \sup_{v_k}\left\|\bar{\phi}(Xv_k)\right\|_2^2 \\ &\leq C\frac{\sup_{v_k}\left\|\bar{\phi}(Xv_k)\right\|_2^2}{p} \leq C\frac{n}{p}\log^2 p,\end{aligned} \quad [240]$$

where the first step is true because of Jensen and triangle inequality, the third step is true because of Eq. (237), and the last step holds because, for every $i \in [n]$,

$$\left|\bar{\phi}(v_k^\top x_i)\right| \leq |\phi(0)| + L\frac{\log p}{L} = |\phi(0)| + \log p \leq C_3\log p, \quad [241]$$

where we use that ϕ is L-Lipschitz and the definition in Eq. (230). We also have

$$\begin{aligned}\mathbb{E}_V\left[H_k\right] &= \mathbb{E}_V[\bar{K}]^{-1/2}\mathbb{E}_V\left[\bar{\phi}(Xv_k)\bar{\phi}(Xv_k^\top)\right]\mathbb{E}_V[\bar{K}]^{-1/2} \\ &= \frac{1}{p}\mathbb{E}_V[\bar{K}]^{-1/2}\mathbb{E}_V[\bar{K}]\mathbb{E}_V[\bar{K}]^{-1/2} \\ &= \frac{1}{p}I,\end{aligned} \quad [242]$$

which allows us to write

$$\begin{aligned}\mathbb{E}_V\left[\left(H_k-\mathbb{E}_V[H_k]\right)^2\right] &= \mathbb{E}_V\left[H_k^2\right] - \mathbb{E}_V\left[H_k\right]^2 \\ &\preceq \mathbb{E}_V\left[H_k^2\right] \\ &\preceq \mathbb{E}_V\left[\sup_{v_k}\left\|H_k\right\|_{\mathrm{op}} H_k\right] \\ &\preceq C\frac{n\log^2 p}{p}\mathbb{E}_V\left[H_k\right] \\ &= C\frac{n\log^2 p}{p^2}I,\end{aligned} \quad [243]$$

where the third line holds since H_k is p.s.d. for all v_k, the fourth line is a consequence of Eq. (240), and the last step of Eq. (242). This readily gives

$$\left\|\mathbb{E}_V\left[\left(H_k-\mathbb{E}_V[H_k]\right)^2\right]\right\|_{\mathrm{op}} \leq \frac{Cn\log^2 p}{p^2}. \quad [244]$$

Thus, Eq. (239) is the sum of p independent (in the probability space of V), mean-0, $n \times n$ symmetric random matrices, such that $\left\| H_k - \mathbb{E}_V[H_k] \right\|_{\text{op}} \le \frac{Cn \log^2 p}{p}$ almost surely for all k. Furthermore, we also have $\left\| \mathbb{E}_V \left[(H_k - \mathbb{E}_V[H_k])^2 \right] \right\|_{\text{op}} \le \frac{Cn \log^2 p}{p^2}$. Then, by Theorem 5.4.1 (31), we get

$$\mathbb{P}_V \left(\left\| \mathbb{E}_V[\bar{K}]^{-1/2} \left(\bar{K} - \mathbb{E}_V[\bar{K}] \right) \mathbb{E}_V[\bar{K}]^{-1/2} \right\|_{\text{op}} \ge t \right) \le 2n \exp \left(- \frac{t^2/2}{\frac{Cn \log^2 p}{p} + \frac{Cn \log^2 p}{3p} t} \right). \tag{245}$$

Setting

$$t = \sqrt{\frac{n}{p}} \log p \log n \le C_4, \tag{246}$$

where the last step holds since $n = \mathcal{O}\left(p / \log^4 p\right)$, we get

$$\begin{aligned} \mathbb{P}_V \left(\left\| \mathbb{E}_V[\bar{K}]^{-1/2} \left(\bar{K} - \mathbb{E}_V[\bar{K}] \right) \mathbb{E}_V[\bar{K}]^{-1/2} \right\|_{\text{op}} \ge \sqrt{\frac{n}{p}} \log n \log p \right) &\le 2 \exp \left(\log n - \frac{\log^2 n}{2C + 2Ct/3} \right) \\ &\le 2 \exp \left(\log n - \frac{\log^2 n}{2C + 2CC_4/3} \right) \\ &\le 2 \exp \left(-c_6 \log^2 n \right), \end{aligned} \tag{247}$$

which gives the result in Eq. (229) on the truncated kernel $\bar{K}$. We will now translate this in the desired result. Note that, by Eq. (234), we have

$$\left\| \mathbb{E}_V[K]^{-1/2} (K - \mathbb{E}_V[K]) \mathbb{E}_V[K]^{-1/2} \right\|_{\text{op}} = \left\| \mathbb{E}_V[K]^{-1/2} \left(\bar{K} - \mathbb{E}_V[K] \right) \mathbb{E}_V[K]^{-1/2} \right\|_{\text{op}} \tag{248}$$

with probability at least $1 - 2\exp\left(-c_2 \log^2 n\right)$ over V. Applying the triangle inequality we get

$$\begin{aligned} &\left\| \mathbb{E}_V[K]^{-1/2} \left(\bar{K} - \mathbb{E}_V[K] \right) \mathbb{E}_V[K]^{-1/2} \right\|_{\text{op}} \\ &\le \left\| \mathbb{E}_V[K]^{-1/2} \left(\bar{K} - \mathbb{E}_V[\bar{K}] \right) \mathbb{E}_V[K]^{-1/2} \right\|_{\text{op}} + \left\| \mathbb{E}_V[K]^{-1/2} \left(\mathbb{E}_V[\bar{K}] - \mathbb{E}_V[K] \right) \mathbb{E}_V[K]^{-1/2} \right\|_{\text{op}} \\ &\le \left\| \mathbb{E}_V[K]^{-1/2} \left(\bar{K} - \mathbb{E}_V[\bar{K}] \right) \mathbb{E}_V[K]^{-1/2} \right\|_{\text{op}} + \left\| \mathbb{E}_V[K]^{-1/2} \right\|_{\text{op}}^2 \left\| \mathbb{E}_V[\bar{K}] - \mathbb{E}_V[K] \right\|_{\text{op}} \\ &\le \left\| \mathbb{E}_V[K]^{-1/2} \left(\bar{K} - \mathbb{E}_V[\bar{K}] \right) \mathbb{E}_V[K]^{-1/2} \right\|_{\text{op}} + \frac{C_5}{p} \exp\left(-c_4 \log^2 p\right), \end{aligned} \tag{249}$$

where the last step follows from Eq. (236) and Lemma 4.3, and it holds with probability at least $1 - 2\exp\left(-c_7 \log^2 n\right)$ over X. Then, we have

$$\begin{aligned} &\left\| \mathbb{E}_V[K]^{-1/2} \left(\bar{K} - \mathbb{E}_V[\bar{K}] \right) \mathbb{E}_V[K]^{-1/2} \right\|_{\text{op}} \\ &\left\| \mathbb{E}_V[K]^{-1/2} \mathbb{E}_V[\bar{K}]^{1/2} \mathbb{E}_V[\bar{K}]^{-1/2} \left(\bar{K} - \mathbb{E}_V[\bar{K}] \right) \mathbb{E}_V[\bar{K}]^{-1/2} \mathbb{E}_V[\bar{K}]^{1/2} \mathbb{E}_V[K]^{-1/2} \right\|_{\text{op}} \\ &\le \left\| \mathbb{E}_V[K]^{-1/2} \mathbb{E}_V[\bar{K}]^{1/2} \right\|_{\text{op}}^2 \left\| \mathbb{E}_V[\bar{K}]^{-1/2} \left(\bar{K} - \mathbb{E}_V[\bar{K}] \right) \mathbb{E}_V[\bar{K}]^{-1/2} \right\|_{\text{op}} \\ &\le 2 \left\| \mathbb{E}_V[\bar{K}]^{-1/2} \left(\bar{K} - \mathbb{E}_V[\bar{K}] \right) \mathbb{E}_V[\bar{K}]^{-1/2} \right\|_{\text{op}}, \end{aligned} \tag{250}$$

where the last step holds since

$$\begin{aligned} \left\| \mathbb{E}_V[K]^{-1/2} \mathbb{E}_V[\bar{K}]^{1/2} \right\|_{\text{op}}^2 &= \left\| \mathbb{E}_V[K]^{-1/2} \mathbb{E}_V[\bar{K}] \mathbb{E}_V[K]^{-1/2} \right\|_{\text{op}} \\ &\le \left\| I \right\|_{\text{op}} + \left\| \mathbb{E}_V[K]^{-1/2} \left(\mathbb{E}_V[\bar{K}] - \mathbb{E}_V[K] \right) \mathbb{E}_V[K]^{-1/2} \right\|_{\text{op}} \\ &\le 1 + \frac{C_5}{p} \exp\left(-c_4 \log^2 p\right), \end{aligned} \tag{251}$$

and the last step of Eq. (251) follows from Eq. (236) and Lemma 4.3, and it holds with probability at least $1 - 2\exp\left(-c_7 \log^2 n\right)$ over X.

Thus, using consecutively Eq. (248), Eq. (249), and Eq. (250), we get

$$\begin{aligned} &\left\| \mathbb{E}_V[K]^{-1/2} (K - \mathbb{E}_V[K]) \mathbb{E}_V[K]^{-1/2} \right\|_{\text{op}} \\ &= \left\| \mathbb{E}_V[K]^{-1/2} \left(\bar{K} - \mathbb{E}_V[K] \right) \mathbb{E}_V[K]^{-1/2} \right\|_{\text{op}} \\ &\le \left\| \mathbb{E}_V[K]^{-1/2} \left(\bar{K} - \mathbb{E}_V[\bar{K}] \right) \mathbb{E}_V[K]^{-1/2} \right\|_{\text{op}} + \frac{C_5}{p} \exp\left(-c_4 \log^2 p\right) \\ &\le 2 \left\| \mathbb{E}_V[\bar{K}]^{-1/2} \left(\bar{K} - \mathbb{E}_V[\bar{K}] \right) \mathbb{E}_V[\bar{K}]^{-1/2} \right\|_{\text{op}} + \frac{C_5}{p} \exp\left(-c_4 \log^2 p\right) \\ &= \mathcal{O}\left(\sqrt{\frac{n}{p}} \log n \log p \right), \end{aligned} \tag{252}$$

where the last step follows from Eq. (247), and the steps jointly hold with probability at least $1-2\exp\left(-c_8\log^2 n\right)$ over X and V. This concludes the proof. $\square$

Lemma 4.5. *Let Assumptions 1 and 2 hold, and let $n=o\left(p/\log^4 p\right)$, $n\log^3 n=o\left(d^{3/2}\right)$ and $n=\omega(d)$. Then, with probability at least $1-2\exp\left(-c\log^2 n\right)$ over X and V, all the following hold*

$$\lambda_d(K)=\Omega\left(\frac{pn}{d}\right), \tag{253}$$

$$\lambda_{d+1}(K)=\mathcal{O}\left(p\right), \tag{254}$$

$$\lambda_n(K)=\lambda_{\min}\left(K\right)=\Omega(p), \tag{255}$$

where c is an absolute constant.

Proof. By Lemma 4.4, we have that, with probability at least $1-2\exp\left(-c_1\log^2 n\right)$ over X and V, for all $u\in\mathbb{R}^n$ we have

$$u^\top\left(\mathbb{E}_V[K]^{-1/2}\left(K-\mathbb{E}_V[K]\right)\mathbb{E}_V[K]^{-1/2}\right)u\le C\sqrt{\frac{n}{p}}\log n\log p\left\|u\right\|_2^2, \tag{256}$$

where C is an absolute constant. Then, setting $u=\mathbb{E}_V[K]^{1/2}\hat{u}$, we have that, for all $\hat{u}\in\mathbb{R}^n$,

$$\hat{u}^\top\left(K-\mathbb{E}_V[K]\right)\hat{u}\le\hat{u}^\top\left(C\sqrt{\frac{n}{p}}\log n\log p\,\mathbb{E}_V[K]\right)\hat{u}, \tag{257}$$

which reads

$$K-\mathbb{E}_V[K]\preceq C\sqrt{\frac{n}{p}}\log n\log p\,\mathbb{E}_V[K]. \tag{258}$$

In the same way, considering instead $(\mathbb{E}_V[K]-K)$ in the very first equation, we can also derive

$$\mathbb{E}_V[K]-K\preceq C\sqrt{\frac{n}{p}}\log n\log p\,\mathbb{E}_V[K], \tag{259}$$

which therefore gives

$$\left(1-C\sqrt{\frac{n}{p}}\log n\log p\right)\mathbb{E}_V[K]\preceq K\preceq\left(1+C\sqrt{\frac{n}{p}}\log n\log p\right)\mathbb{E}_V[K]. \tag{260}$$

By the Courant–Fischer–Weyl min-max principle, we can write

$$\lambda_i(K)=\max_S\min_{u\in S,\,\|u\|_2=1}\left(u^\top Ku\right), \tag{261}$$

where S is any i-dimensional subspace of $\mathbb{R}^n$. Let $S^*=\arg\max_S\min_{u\in S,\,\|u\|_2=1}\left(u^\top Ku\right)$. Then, by Eq. (260), we have

$$\begin{aligned}\lambda_i(K)&=\min_{u\in S^*,\,\|u\|_2=1}\left(u^\top Ku\right)\\&\le\min_{u\in S^*,\,\|u\|_2=1}\left(u^\top\left(\left(1+C\sqrt{\frac{n}{p}}\log n\log p\right)\mathbb{E}_V[K]\right)u\right)\\&\le\max_S\min_{u\in S,\,\|u\|_2=1}\left(u^\top\left(\left(1+C\sqrt{\frac{n}{p}}\log n\log p\right)\mathbb{E}_V[K]\right)u\right)\\&=\left(1+C\sqrt{\frac{n}{p}}\log n\log p\right)\lambda_i\left(\mathbb{E}_V[K]\right),\end{aligned} \tag{262}$$

where in the last line we use the same principle to express the i-th eigenvalue of $\mathbb{E}_V[K]$. Following the same strategy, we can therefore conclude that

$$\left(1-C\sqrt{\frac{n}{p}}\log n\log p\right)\lambda_i\left(\mathbb{E}_V[K]\right)\le\lambda_i\left(K\right)\le\left(1+C\sqrt{\frac{n}{p}}\log n\log p\right)\lambda_i\left(\mathbb{E}_V[K]\right). \tag{263}$$

By Lemma 4.3, we have that, with probability at least $1-2\exp\left(-c_2\log n\right)$ over X, $\lambda_d\left(\mathbb{E}_V[K]\right)=\Omega(np/d)$, and that $\lambda_i\left(\mathbb{E}_V[K]\right)=\Theta(p)$, for $d+1\le i\le n$. This implies

$$\lambda_d(K)=\Omega\left(\left(1-C\sqrt{\frac{n}{p}}\log n\log p\right)\frac{np}{d}\right)=\Omega\left(\frac{pn}{d}\right), \tag{264}$$

$$\lambda_{d+1}(K) = \mathcal{O}\left(\left(1 + C\sqrt{\frac{n}{p}}\log n \log p\right) p\right) = \mathcal{O}\left(p\right), \tag{265}$$

$$\lambda_n(K) = \lambda_{\min}\left(K\right) = \Omega\left(\left(1 - C\sqrt{\frac{n}{p}}\log n \log p\right) p\right) = \Omega(p), \tag{266}$$

where we use $n\log^2 n = o(p/\log^2 p)$. This gives the desired result. □

Lemma 4.6. *Let Assumptions 1 and 2 hold, and let $d = o(p)$ and $d = o(n)$. Then, we have*

$$\left\|\Phi\right\|_{\mathrm{op}} = \mathcal{O}\left(\sqrt{\frac{np}{d}}\right), \tag{267}$$

with probability at least $1 - 2\exp\left(-cd\right)$ over X and V, where c is an absolute constant.

Proof. The k-th row of $\Phi^\top$ takes the form $\phi(Xv_k) \in \mathbb{R}^n$. Since the Gaussian distribution is Lipschitz concentrated (see Theorem 5.22 in (31)), we have that

$$\left\|\phi(Xv_k)\right\|_{\psi_2} = \left\|\phi(Xv_k) - \mathbb{E}_{v_k}\left[\phi(Xv_k)\right]\right\|_{\psi_2} \leq C_1 \frac{\left\|X\right\|_{\mathrm{op}}}{\sqrt{d}}, \tag{268}$$

where the first step holds since the 0-th Hermite coefficient of ϕ is zero, and the second step holds since ϕ is Lipschitz. Notice that the term $\sqrt{d}$ is due to the fact that $\sqrt{d}\, v_k$ is standard Gaussian. We remark that the sub-Gaussian norm in this equation is intended in the probability space of v_k. By Lemma 4.2, we have that

$$\left\|X\right\|_{\mathrm{op}} = \mathcal{O}\left(\sqrt{n}\right), \tag{269}$$

with probability at least $1 - 2\exp\left(-c_1 d\right)$ over X. Thus, conditioning on this high probability event, due to Eq. (268), we have that $\Phi^\top$ is a $p \times n$ matrix whose rows are i.i.d. mean-0 random vectors with sub-Gaussian norm $\mathcal{O}\left(\sqrt{n/d}\right)$. Then, by Lemma B.7 of (37), we have

$$\left\|\Phi^\top\right\|_{\mathrm{op}} = \mathcal{O}\left(\sqrt{\frac{np}{d}}\right), \tag{270}$$

with probability at least $1 - 2\exp(-c_2 n)$ over V, which provides the desired result. □

Lemma 4.7. *Let Assumptions 1 and 2 hold, and let $n\log^3 n = \mathcal{O}\left(d^{3/2}\right)$. Then, we have*

$$\left\|\mathbb{E}_V\left[\tilde{K}\right]\right\|_{\mathrm{op}} = \mathcal{O}\left(p\right), \tag{271}$$

with probability at least $1 - 2\exp\left(-c\log^2 n\right)$ over X, where c is an absolute constant.

Proof. The proof follows the same strategy as the proof of Lemma 4.3, with the difference that now there is no term M_1, as $\tilde{\phi}$ share the same Hermite coefficients with ϕ, but with $\tilde{\mu}_1 = 0$. The thesis then follows from Eq. (224). We remark that to prove this lemma, differently from the proof of lemma 4.3, $n = \omega(d)$ is not required. Also, we require only $n\log^3 n = \mathcal{O}\left(d^{3/2}\right)$ instead of $n\log^3 n = o\left(d^{3/2}\right)$. In fact, we do not need a bound on the smallest eigenvalue of M_2 (see Eq. (224)), and therefore we just need $\left\|M_2 - \tilde{M}_2\right\|_{\mathrm{op}} = \mathcal{O}\left(p\right)$ (see Eq. (223)). □

Lemma 4.8. *Let Assumptions 1 and 2 hold, and let $n = \mathcal{O}\left(p/\log^4 p\right)$ and $n\log^3 n = \mathcal{O}\left(d^{3/2}\right)$. Then, we have*

$$\left\|\tilde{\Phi}\right\|_{\mathrm{op}} = \mathcal{O}\left(\sqrt{p}\right), \tag{272}$$

with probability at least $1 - 2\exp\left(-c\log^2 n\right)$ over X and V, where c is an absolute constant.

Proof. Consider the truncated function

$$\bar{\phi}(z) := \tilde{\phi}(z)\mathbf{1}\left(|z| \leq \frac{\log p}{\tilde{L}}\right), \tag{273}$$

where $\tilde{L}$ is the Lipschitz constant of $\tilde{\phi}$. Define the truncated kernel $\bar{K}$ accordingly

$$\bar{K}_{ij} := \bar{\phi}(Vx_i)^\top \bar{\phi}(Vx_j). \tag{274}$$

We now compare $\mathbb{E}_V\left[\bar{K}\right]$ and $\mathbb{E}_V[\tilde{K}]$. Let $v \sim \mathcal{N}(0, I/d)$ and define the random variable

$$E_{ij} := \mathbf{1}\left(|v^\top x_i| > \frac{\log p}{\tilde{L}} \;\text{ or }\; |v^\top x_j| > \frac{\log p}{\tilde{L}}\right). \tag{275}$$

Note that since E_{ij} is an indicator we have $E_{ij} = E_{ij}^2$, and

$$\mathbb{P}_V(E_{ij}=1) \le \mathbb{P}_V\left(|v^\top x_i| > \frac{\log p}{\tilde{L}}\right) + \mathbb{P}_V\left(|v^\top x_j| > \frac{\log p}{\tilde{L}}\right) \le \exp\left(-c_1 \log^2 p\right), \tag{276}$$

where the second step holds as $v^\top x_i \sim \mathcal{N}(0,1)$ in the probability space of V.

Thus, we can write

$$\begin{aligned} \left|\mathbb{E}_V\left[\bar{K}_{ij}\right] - \mathbb{E}_V[\tilde{K}_{ij}]\right| &= p\left|\mathbb{E}_v\left[\tilde{\phi}(v^\top x_i)\tilde{\phi}(v^\top x_j)\, E_{ij}\right]\right| \\ &\le p\,\mathbb{E}_v\left[\left(\tilde{\phi}(v^\top x_i)\tilde{\phi}(v^\top x_j)\right)^2\right]^{1/2} \mathbb{E}_v\left[E_{ij}^2\right]^{1/2} \\ &\le p\,\mathbb{E}_v\left[\left(\tilde{\phi}(v^\top x_i)\right)^4\right]^{1/2} \mathbb{P}_V(E_{ij}=1)^{1/2} \\ &\le p\, C_1^{1/2} \exp\left(-\frac{c_1 \log^2 p}{2}\right) \\ &\le p \exp\left(-c_2 \log^2 p\right). \end{aligned} \tag{277}$$

Here, the second and third lines follow from Cauchy-Schwartz inequality; the fourth line is a consequence of Eq. (276) and $\tilde{\phi}(v^\top x_i)$ being a sub-Gaussian random variable ($\tilde{\phi}(z)$ is Lipschitz), and thus with bounded fourth moment (see Equation 2.11 in (31)). This result holds for any i, j, and therefore implies

$$\begin{aligned} \left\|\mathbb{E}_V\left[\bar{K}\right] - \mathbb{E}_V[\tilde{K}]\right\|_{\text{op}} &\le \left\|\mathbb{E}_V\left[\bar{K}\right] - \mathbb{E}_V[\tilde{K}]\right\|_F \\ &\le pn\exp\left(-c_2\log^2 p\right) \\ &\le \exp\left(-c_3 \log^2 p\right), \end{aligned} \tag{278}$$

where in the last step we used $n = \mathcal{O}(p)$, which follows from Assumption 3. This last equation, together with Lemma 4.7, gives

$$\left\|\mathbb{E}_V\left[\bar{K}\right]\right\|_{\text{op}} = \mathcal{O}(p), \tag{279}$$

with probability at least $1 - \exp\left(-c_4 \log^2 n\right)$. We will condition on this high probability event until the end of the proof.

We now define the matrix $\bar{H}_k \in \mathbb{R}^{n\times n}$ as

$$\bar{H}_k = \bar{\phi}(Xv_k)\bar{\phi}(Xv_k)^\top, \tag{280}$$

which implies

$$\bar{\Phi}\bar{\Phi}^\top - \mathbb{E}_V\left[\bar{\Phi}\bar{\Phi}^\top\right] = \sum_{k=1}^p \bar{H}_k - \mathbb{E}_V\left[\bar{H}_k\right]. \tag{281}$$

Note that there exists a natural constant C such that

$$\sup_{v_k}\left\|\bar{H}_k - \mathbb{E}_V[\bar{H}_k]\right\|_{\text{op}} \le 2\sup_{v_k}\left\|\bar{H}_k\right\|_{\text{op}} \le 2\sup_{v_k}\left\|\bar{\phi}(Xv_k)\right\|_2^2 \le 2n\log^2 p, \tag{282}$$

where the first step is true because of Jensen and triangle inequality, and the last step holds because, for every $i \in [n]$,

$$\left|\bar{\phi}(v_k^\top x_i)\right| \le \tilde{L}\frac{\log p}{\tilde{L}} = \log p, \tag{283}$$

given the definition in Eq. (273). Also, note that we have

$$\mathbb{E}_V\left[\bar{H}_k\right] = \mathbb{E}_V\left[\bar{\phi}(Xv_k)\bar{\phi}(Xv_k)^\top\right] = \frac{1}{p}\mathbb{E}_V[\bar{K}], \tag{284}$$

which gives, together with Eq. (279),

$$\left\|\mathbb{E}_V\left[\bar{H}_k\right]\right\|_{\text{op}} = \mathcal{O}(1). \tag{285}$$

Then, we can use Eq. (282) and Eq. (285), together with the same argument in Eq. (243), to get

$$\left\|\mathbb{E}_V\left[\left(\bar{H}_k - \mathbb{E}_V[\bar{H}_k]\right)^2\right]\right\|_{\text{op}} = \mathcal{O}\left(n\log^2 p\right). \tag{286}$$

Thus, Eq. (281) is the sum of p independent (in the probability space of V), mean-0, $n\times n$ symmetric random matrices, such that $\left\|\bar{H}_k - \mathbb{E}_V[\bar{H}_k]\right\|_{\text{op}} \le 2n\log^2 p$ almost surely for all k, and $\left\|\mathbb{E}_V\left[\left(\bar{H}_k - \mathbb{E}_V[\bar{H}_k]\right)^2\right]\right\|_{\text{op}} \le C_1 n \log^2 p$, for some absolute constant C_1. Then, by Theorem 5.4.1 (31), we get

$$\mathbb{P}\left(\left\|\bar{\Phi}\bar{\Phi}^\top - \mathbb{E}_V\left[\bar{\Phi}\bar{\Phi}^\top\right]\right\|_{\text{op}} \ge t\right) \le 2n\exp\left(-\frac{t^2/2}{C_1 np\log^2 p + 2tn\log^2 p/3}\right). \tag{287}$$

Setting $t = p$, we get

$$\mathbb{P}\left(\left\|\bar{\Phi}\bar{\Phi}^\top - \mathbb{E}_V\left[\bar{\Phi}\bar{\Phi}^\top\right]\right\|_{\text{op}} \geq p\right) \leq 2\exp\left(-c_5\frac{p}{n\log^2 p}\right) \leq 2\exp\left(-c_6\log^2 n\right), \tag{288}$$

where in the last step we used $n = \mathcal{O}\left(p/\log^4 p\right)$. To conclude, we have that

$$\begin{aligned}\mathbb{P}_V(\tilde{\Phi}\tilde{\Phi}^\top \neq \bar{\Phi}\bar{\Phi}^\top) &\leq \mathbb{P}_V\left(\max_{i\in[n],k\in[p]}|v_k^\top x_i| > \log p/\tilde{L}\right)\\ &\leq 2np\exp\left(-c_6\log^2 p\right)\\ &\leq 2\exp\left(-c_7\log^2 p\right),\end{aligned} \tag{289}$$

where the second step holds as $v_k^\top x_i \sim \mathcal{N}(0,1)$ in the probability space of V, and the last step holds because of the assumption $n = \mathcal{O}\left(p/\log^4 p\right)$. Putting Eq. (288) and Eq. (289) together, we finally get

$$\left\|\tilde{\Phi}\tilde{\Phi}^\top\right\|_{\text{op}} = \left\|\bar{\Phi}\bar{\Phi}^\top\right\|_{\text{op}} \leq \left\|\mathbb{E}_V\left[\bar{\Phi}\bar{\Phi}^\top\right]\right\|_{\text{op}} + \left\|\bar{\Phi}\bar{\Phi}^\top - \mathbb{E}_V\left[\bar{\Phi}\bar{\Phi}^\top\right]\right\|_{\text{op}} = \mathcal{O}(p), \tag{290}$$

with probability at least $1 - 2\exp\left(-c\log^2 n\right)$ over X and V, which gives the thesis. □

Lemma 4.9. *Let Assumptions 1 and 2 hold, and $p = \omega(n)$. Then, we have*

$$\left|\|\varphi(x_1)\|_2^2 - Mp\right| = \mathcal{O}(\sqrt{p}\log n), \tag{291}$$

$$\left|\|\tilde{\varphi}(x_1)\|_2^2 - M_1 p\right| = \mathcal{O}(\sqrt{p}\log n), \tag{292}$$

with M and M_1 being two positive constants only depending on ϕ, with probability at least $1 - 2\exp(-c\log^2 n)$ over V, where c is an absolute constant.

Furthermore, we have that $M - M_1 = \mu_1^2 > 0$, which, conditioning on the previous result, implies

$$\|\varphi(x_1)\|_2 - \|\tilde{\varphi}(x_1)\|_2 = \Omega(\sqrt{p}). \tag{293}$$

Proof. We have

$$\|\varphi(x_1)\|_2^2 = \sum_{k=1}^p \phi(v_k^\top x_1)^2, \tag{294}$$

where we use the shorthand v_k to indicate the k-th row of V. As ϕ is Lipschitz, $v_j \sim \mathcal{N}(0, I/d)$, and $\|x_1\|_2 = \sqrt{d}$, we have that $\|\varphi(x_1)\|_2^2$ is the sum of p independent sub-exponential random variables, in the probability space of V. Thus, by Bernstein inequality (see Theorem 2.8.1 in (31)), we have

$$\left|\|\varphi(x_1)\|_2^2 - \mathbb{E}_V\left[\|\varphi(x_1)\|_2^2\right]\right| = \mathcal{O}(\sqrt{p}\log n), \tag{295}$$

with probability at least $1 - \exp(-c_1\log^2 n)$, over the probability space of V. Exploiting the Hermite expansion of ϕ, we get

$$\mathbb{E}_V\left[\|\varphi(x_1)\|_2^2\right] = p\,\mathbb{E}_{\rho\sim(0,1)}\left[\phi(\rho)^2\right] = pM, \tag{296}$$

where we set $M = \sum_{l=0}^\infty \mu_l^2$.

The same argument applied on $\tilde{\phi}$, which is also Lipschitz, guarantees

$$\left|\|\tilde{\varphi}(x_1)\|_2^2 - M_1 p\right| = \mathcal{O}(\sqrt{p}\log n), \tag{297}$$

where $M_1 = \sum_{l=3}^\infty \mu_l^2$, since the 0-th, 1st and 2nd Hermite coefficients of $\tilde{\phi}$ are 0. Thus, we readily get

$$M - M_1 = \mu_1^2. \tag{298}$$

To conclude, it is sufficient to notice that, conditioning on the previous two high probability events, we have that $\|\varphi(x_1)\|_2$ and $\|\tilde{\varphi}(x_1)\|_2$ are both $\mathcal{O}\left(\sqrt{p}\right)$. Then,

$$\begin{aligned}\|\varphi(x_1)\|_2 - \|\tilde{\varphi}(x_1)\|_2 &= \frac{\|\varphi(x_1)\|_2^2 - \|\tilde{\varphi}(x_1)\|_2^2}{\|\varphi(x_1)\|_2 + \|\tilde{\varphi}(x_1)\|_2}\\ &\geq \frac{(M - M_1)p - \left|\|\varphi(x_1)\|_2^2 - pM\right| - \left|\|\tilde{\varphi}(x_1)\|_2^2 - M_1 p\right|}{\|\varphi(x_1)\|_2 + \|\tilde{\varphi}(x_1)\|_2}\\ &= \Omega(\sqrt{p}),\end{aligned} \tag{299}$$

where we use the triangle inequality twice in the second line and conclude using $\sqrt{p} = \omega(\log n)$. □

Lemma 4.10. *Let Assumptions 1 and 2 hold, and let $n = o\left(p/\log^4 p\right)$, $n\log^3 n = o\left(d^{3/2}\right)$ and $n = \omega(d)$. Then, we jointly have*

$$\left\|X^+\right\|_{\text{op}} = \mathcal{O}\left(\frac{1}{\sqrt{n}}\right), \tag{300}$$

$$\left\|V^+\right\|_{\text{op}} = \mathcal{O}\left(\sqrt{\frac{d}{p}}\right), \tag{301}$$

$$\left\|\mu_1 V^\top - X^+\Phi\right\|_{\text{op}} = \mathcal{O}\left(\sqrt{\frac{p}{n}}\right), \tag{302}$$

$$\left\|\mu_1 V^\top \Phi^+\right\|_{\text{op}} = \mathcal{O}\left(\frac{1}{\sqrt{n}}\right), \tag{303}$$

with probability at least $1 - 2\exp\left(-c\log^2 n\right)$ over X and V, where c is an absolute constant.

Proof. The first two statements easily follow from Lemma 4.2, which gives

$$\left\|X^+\right\|_{\text{op}} = \lambda_{\min}\left(X^\top X\right)^{-1/2} = \mathcal{O}\left(\frac{1}{\sqrt{n}}\right), \tag{304}$$

$$\left\|V^+\right\|_{\text{op}} = \lambda_{\min}\left(V^\top V\right)^{-1/2} = \mathcal{O}\left(\sqrt{\frac{d}{p}}\right), \tag{305}$$

with probability at least $1 - 2\exp(-c_1 d) \geq 1 - 2\exp\left(-c_2\log^2 n\right)$ over X and V. For the third statement, we condition on this high probability event, and notice that

$$\Phi = \mu_1 X V^\top + \tilde{\Phi}, \tag{306}$$

which gives

$$\left\|\mu_1 V^\top - X^+\Phi\right\|_{\text{op}} = \left\|X^+\tilde{\Phi}\right\|_{\text{op}} \leq \left\|X^+\right\|_{\text{op}}\left\|\tilde{\Phi}\right\|_{\text{op}} = \mathcal{O}\left(\sqrt{\frac{p}{n}}\right), \tag{307}$$

where the last step holds because of Lemma 4.8 with probability at least $1 - 2\exp\left(-c_3\log^2 n\right)$. For the fourth statement, we write

$$\begin{aligned}\left\|\mu_1 V^\top \Phi^+\right\|_{\text{op}} &\leq \left\|\left(\mu_1 V^\top - X^+\Phi\right)\Phi^+\right\|_{\text{op}} + \left\|X^+\Phi\Phi^+\right\|_{\text{op}} \\ &\leq \left\|\mu_1 V^\top - X^+\Phi\right\|_{\text{op}}\left\|\Phi^+\right\|_{\text{op}} + \left\|X^+\right\|_{\text{op}} \\ &= \mathcal{O}\left(\sqrt{\frac{p}{n}}\sqrt{\frac{1}{p}} + \frac{1}{\sqrt{n}}\right) = \mathcal{O}\left(\frac{1}{\sqrt{n}}\right),\end{aligned} \tag{308}$$

where the second step holds since

$$\left\|\Phi^+\right\|_{\text{op}} = \lambda_{\min}(K)^{-1/2} = \mathcal{O}\left(\frac{1}{\sqrt{p}}\right), \tag{309}$$

which holds with probability $1 - 2\exp\left(-c_4\log^2 n\right)$ because of Lemma 4.5. This final result provides the thesis. □

Lemma 4.11. *Let Assumptions 1 and 2 hold, and let $n = o\left(p/\log^4 p\right)$, $n\log^3 n = o\left(d^{3/2}\right)$ and $n = \omega(d)$. Let $P_\Lambda \in \mathbb{R}^{p\times p}$ be the projector on the span of the d eigenvectors corresponding to the d highest eigenvalues of $\Phi^\top\Phi$, and P_V be the projector on the span of the columns of V. Then, we have*

$$\left\|P_\Lambda^\perp V\right\|_{\text{op}} = \mathcal{O}\left(\sqrt{\frac{p}{n}}\right), \tag{310}$$

and

$$\left\|P_\Lambda^\perp P_V\right\|_{\text{op}} = \mathcal{O}\left(\sqrt{\frac{d}{n}}\right), \tag{311}$$

jointly hold with probability at least $1 - 2\exp\left(-c\log^2 n\right)$ over X and V, where c is an absolute constant.

Proof. Let $u \in \mathbb{R}^p$ be a vector such that $\|u\|_2 = 1$. Then, we have

$$\left\|\Phi P_\Lambda^\perp u\right\|_2 \leq \sqrt{\lambda_{d+1}(\Phi^\top\Phi)} = \sqrt{\lambda_{d+1}(K)} = \mathcal{O}\left(\sqrt{p}\right), \tag{312}$$

where the first inequality follows by the definition of P_Λ, and $\lambda_{d+1}(K)$ ($\lambda_{d+1}(\Phi^\top\Phi)$) is the $(d+1)$-th eigenvalue of K ($\Phi^\top\Phi$) when sorting them in non-increasing order. Then, the last equality holds with probability at least $1-2\exp\left(-c_1\log^2 n\right)$ over X and V, because of Lemma 4.5. Then, conditioning on the previous high probability event, since $\Phi = \tilde{\Phi} + \mu_1 X V^\top$, we can write

$$\begin{aligned}\mu_1\sqrt{\lambda_{\min}\left(X^\top X\right)}\left\|V^\top P_\Lambda^\perp u\right\|_2 &\le \left\|\mu_1 X V^\top P_\Lambda^\perp u\right\|_2 \\ &\le \left\|\tilde{\Phi} P_\Lambda^\perp u\right\|_2 + \left\|\Phi P_\Lambda^\perp u\right\|_2 \\ &\le \left\|\tilde{\Phi}\right\|_{\text{op}}\left\|P_\Lambda^\perp\right\|_{\text{op}}\|u\|_2 + \left\|\Phi P_\Lambda^\perp u\right\|_2 = \mathcal{O}\left(\sqrt{p}\right),\end{aligned} \tag{313}$$

where the last step is a consequence of Lemma 4.8, and it holds with probability $1-2\exp\left(-c_2\log^2 n\right)$. By Lemma 4.2, we have that $\lambda_{\min}\left(X^\top X\right) = \Theta(n)$ with probability at least $1-2\exp\left(-c_3 d\right)$. Conditioning on such high probability events, Eq. (313) gives

$$\left\|V^\top P_\Lambda^\perp u\right\|_2 = \mathcal{O}\left(\sqrt{\frac{p}{n}}\right), \tag{314}$$

which provides the first part of the thesis, since it uniformly holds for every u (as we did not condition on any event dependent on u itself). To conclude, since we have

$$P_V = (V^+)^\top V^\top, \tag{315}$$

we can use Lemma 4.10 to write

$$\left\|P_V P_\Lambda^\perp u\right\|_2 = \left\|(V^+)^\top V^\top P_\Lambda^\perp u\right\|_2 \le \left\|V^+\right\|_{\text{op}}\left\|V^\top P_\Lambda^\perp u\right\|_2 = \mathcal{O}\left(\sqrt{\frac{d}{n}}\right), \tag{316}$$

with probability at least $1-2\exp\left(-c_4 d\right)$ over V. Since again this result holds uniformly on all $\|u\|_2 = 1$, and $\left\|P_\Lambda^\perp P_V\right\|_{\text{op}} = \left\|P_V P_\Lambda^\perp\right\|_{\text{op}}$, the thesis readily follows. □

Lemma 4.12. *Let Assumptions 1 and 2 hold, and let $n = o\left(p/\log^4 p\right)$, $n\log^3 n = o\left(d^{3/2}\right)$ and $n = \omega(d)$. Let $P_\Lambda \in \mathbb{R}^{p\times p}$ be the projector on the span of the d eigenvectors corresponding to the d highest eigenvalues of $\Phi^\top\Phi$. Then, we have*

$$\left\|P_\Lambda \varphi(x_1)\right\|_2 = \Omega(\sqrt{p}), \tag{317}$$

with probability at least $1-2\exp\left(-c\log^2 n\right)$ over X and V, where c is an absolute constant.

Proof. Multiple applications of the triangle inequality yield

$$\begin{aligned}\left\|P_\Lambda\varphi(x_1)\right\|_2 &\ge \left\|\varphi(x_1)\right\|_2 - \left\|P_\Lambda^\perp\varphi(x_1)\right\|_2 \\ &= \left\|\varphi(x_1)\right\|_2 - \left\|P_\Lambda^\perp\left(\mu_1 V x_1 + \tilde{\varphi}(x_1)\right)\right\|_2 \\ &\ge \left\|\varphi(x_1)\right\|_2 - \mu_1\left\|P_\Lambda^\perp V x_1\right\|_2 - \left\|P_\Lambda^\perp\tilde{\varphi}(x_1)\right\|_2 \\ &\ge \left\|\varphi(x_1)\right\|_2 - \mu_1\left\|P_\Lambda^\perp V\right\|_{\text{op}}\left\|x_1\right\|_2 - \left\|\tilde{\varphi}(x_1)\right\|_2.\end{aligned} \tag{318}$$

Let's condition on the thesis of Lemma 4.11 and Lemma 4.9 to hold. Then, we have that

$$\left\|\varphi(x_1)\right\|_2 - \left\|\tilde{\varphi}(x_1)\right\|_2 = \Omega(\sqrt{p}), \tag{319}$$

and

$$\left\|P_\Lambda^\perp V\right\|_{\text{op}}\left\|x_1\right\|_2 = \mathcal{O}\left(\sqrt{\frac{p}{n}}\right)\sqrt{d} = o(\sqrt{p}), \tag{320}$$

which readily gives the thesis. □

Lemma 4.13. *We have that*

$$\left\|\tilde{\Phi} V\right\|_{\text{op}} = \mathcal{O}\left(\sqrt{\frac{pn}{d}}\log n\right), \tag{321}$$

with probability at least $1-2\exp\left(-c\log^2 n\right)$ over X and V, where c is an absolute constant.

Proof. Note that $\tilde{\phi}$ is Lipschitz (since ϕ is Lipschitz by Assumption 2). During the proof, we condition on the event $\|X\|_{\text{op}} = \mathcal{O}\left(\sqrt{n}\right)$, which is true with probability at least $1-2\exp\left(-c_1 d\right)$ by Lemma 4.2, and we use the shorthand $v \in \mathbb{R}^d$ to denote a random vector such that $\sqrt{d}\,v$ is a standard Gaussian vector, *i.e.*, it has the same distribution as the rows of V. This implies

$$\mathbb{E}_v\left[\left\|\tilde{\phi}\left(Xv\right)\right\|_2\right] = \mathcal{O}\left(\sqrt{n}\right), \qquad \left\|\left\|\tilde{\phi}\left(Xv\right)\right\|_2 - \mathbb{E}_v\left[\left\|\tilde{\phi}\left(Xv\right)\right\|_2\right]\right\|_{\psi_2} = \mathcal{O}\left(\sqrt{\frac{n}{d}}\right), \tag{322}$$

and

$$\mathbb{E}_v\left[\|v\|_2\right]=\mathcal{O}(1), \qquad \left\|\|v\|_2-\mathbb{E}_v\left[\|v\|_2\right]\right\|_{\psi_2}=\mathcal{O}\left(\frac{1}{\sqrt{d}}\right), \tag{323}$$

where both sub-Gaussian norms are meant on the probability space of v. Here, the very first equation follows from the discussion in Lemma C.3 in (37), and the upper bounds on the sub-Gaussian norms follow from the Lipschitz concentration property of $\sqrt{d}v$. Then, there exists an absolute constant C_1 such that we jointly have

$$\left\|\tilde{\phi}(Xv)\right\|_2 \leq C_1\sqrt{n}, \qquad \|v\|_2 \leq C_1, \tag{324}$$

with probability at least $1-2\exp(-c_2 d)$ over v.

Let E_k be the indicator defined on the high probability event above with respect to the random variable $v_k := V_{:k}$ (the k-th row of V), *i.e.*,

$$E_k := \mathbf{1}\left(\|v_k\|_2 \leq C_1 \text{ and } \left\|\tilde{\phi}(Xv_k)\right\|_2 \leq C_1\sqrt{n}\right), \tag{325}$$

and we define $E \in \mathbb{R}^{p\times p}$ as the diagonal matrix containing E_k in its k-th entry. Notice that we have $\|I-E\|_{\mathrm{op}}=0$ with probability at least $1-2p\exp(-c_2 d)$, and $\mathbb{E}_V\left[\|I-E\|_{\mathrm{op}}\right] \leq 2p\exp(-c_2 d)$.

Thus, we have

$$\begin{aligned}
\left\|\mathbb{E}_V\left[\tilde{\Phi}(I-E)V\right]\right\|_{\mathrm{op}} &\leq \mathbb{E}_V\left[\left\|\tilde{\Phi}\right\|_{\mathrm{op}}\|1-E\|_{\mathrm{op}}\|V\|_{\mathrm{op}}\right]\\
&\leq \mathbb{E}_V\left[\left\|\tilde{\Phi}\right\|_{\mathrm{op}}^2\|V\|_{\mathrm{op}}^2\right]^{1/2}\mathbb{E}_V\left[\|I-E\|_{\mathrm{op}}^2\right]^{1/2}\\
&\leq \mathbb{E}_V\left[\left\|\tilde{\Phi}\right\|_{\mathrm{op}}^4\right]^{1/4}\mathbb{E}_V\left[\|V\|_{\mathrm{op}}^4\right]^{1/4}(2p\exp(-c_2 d))^{1/2}\\
&\leq \mathbb{E}_V\left[\left\|\tilde{\Phi}\right\|_{F}^4\right]^{1/4}\mathbb{E}_V\left[\|V\|_{F}^4\right]^{1/4}(2p\exp(-c_2 d))^{1/2}\\
&= o(1),
\end{aligned} \tag{326}$$

where the last step holds because of our initial conditioning on X: the first two terms are the sum of finite powers of sub-Gaussian random variables (the entries of $\tilde{\Phi}$ and V), and thus (see Proposition 2.5.2 in (31)) the first two factors in the third line of the previous equation will be $\mathcal{O}(p^\alpha)$ for some finite α, which gives the last line due to Assumption 3.

Exploiting the Hermite expansion of $\tilde{\phi}$ we can write

$$\left[\mathbb{E}_V\left[\tilde{\Phi}V\right]\right]_{ij} = p\left[\mathbb{E}_v\left[\tilde{\phi}(Xv)v^\top\right]\right]_{ij} = \frac{p}{\sqrt{d}}\mathbb{E}_v\left[\tilde{\phi}\left(x_i^\top v\right)\left(e_j^\top\left(\sqrt{d}v\right)\right)\right]=0, \tag{327}$$

where the last step holds since the first Hermite coefficient of $\tilde{\phi}$ is 0. Thus, the application of the triangle inequality to this last equation and Eq. (326) gives

$$\left\|\mathbb{E}_V\left[\tilde{\Phi}EV\right]\right\|_{\mathrm{op}} \leq \left\|\mathbb{E}_V\left[\tilde{\Phi}V\right]\right\|_{\mathrm{op}} + \left\|\mathbb{E}_V\left[\tilde{\Phi}(I-E)V\right]\right\|_{\mathrm{op}} = o(1), \tag{328}$$

with probability at least $1-2\exp\left(-c_3\log^2 d\right)$ over X.

Let's now look at

$$\tilde{\Phi}EV - \mathbb{E}_V\left[\tilde{\Phi}EV\right] = \sum_{k=1}^{p}\tilde{\phi}(Xv_k)E_k v_k^\top - \mathbb{E}_{v_k}\left[\tilde{\phi}(Xv_k)E_k v_k^\top\right] =: \sum_{k=1}^{p} W_k, \tag{329}$$

where we defined the shorthand $W_k = \tilde{\phi}(Xv_k)E_k v_k^\top - \mathbb{E}_{v_k}\left[\tilde{\phi}(Xv_k)E_k v_k^\top\right]$. Eq. (329) is the sum of p i.i.d. mean-0 random matrices W_k (in the probability space of V), such that

$$\begin{aligned}
\sup_{v_k}\left\|\tilde{\phi}(Xv_k)E_k v_k^\top - \mathbb{E}_{v_k}\left[\tilde{\phi}(Xv_k)E_k v_k^\top\right]\right\|_{\mathrm{op}} &\leq 2\sup_{v_k}\left\|\tilde{\phi}(Xv_k)E_k v_k^\top\right\|_{\mathrm{op}}\\
&= 2\sup_{v_k}\left(\left\|\tilde{\phi}(Xv_k)\right\|_2\|v_k\|_2 E_k\right)\\
&\leq 2C_1^2\sqrt{n},
\end{aligned} \tag{330}$$

because of Eq. (325). Then, by matrix Bernstein's inequality for rectangular matrices (see Exercise 5.4.15 in (31)), we have that

$$\mathbb{P}_V\left(\left\|\tilde{\Phi}EV - \mathbb{E}_V\left[\tilde{\Phi}EV\right]\right\|_{\mathrm{op}} \geq t\right) \leq (n+d)\exp\left(-\frac{t^2/2}{\sigma^2+2C_1^2\sqrt{n}t/3}\right), \tag{331}$$

where σ^2 is defined as

$$\sigma^2 = p\max\left(\left\|\mathbb{E}_{v_k}\left[W_k W_k^\top\right]\right\|_{\mathrm{op}}, \left\|\mathbb{E}_{v_k}\left[W_k^\top W_k\right]\right\|_{\mathrm{op}}\right). \tag{332}$$

For every matrix A, we have $\mathbb{E}\left[(A-\mathbb{E}[A])(A-\mathbb{E}[A])^\top\right]=\mathbb{E}\left[AA^\top\right]-\mathbb{E}[A]\mathbb{E}[A]^\top\preceq\mathbb{E}\left[AA^\top\right]$. Thus,

$$\begin{aligned}\left\|\mathbb{E}_{v_k}\left[W_kW_k^\top\right]\right\|_{\rm op}&\leq\left\|\mathbb{E}_{v_k}\left[\tilde\phi(Xv_k)E_kv_k^\top v_kE_k\tilde\phi(Xv_k)^\top\right]\right\|_{\rm op}\\&\leq\left\|\mathbb{E}_{v_k}\left[\tilde\phi(Xv_k)\tilde\phi(Xv_k)^\top\right]\right\|_{\rm op}\sup_{v_k}\left(E_k\left\|v_k\right\|_2^2\right)\\&\leq C_1^2\left\|\mathbb{E}_{v_k}\left[\tilde\phi(Xv_k)\tilde\phi(Xv_k)^\top\right]\right\|_{\rm op}\\&=\frac{C_1^2}{p}\left\|\mathbb{E}_V\left[\tilde K\right]\right\|_{\rm op}\\&=\mathcal{O}\left(1\right),\end{aligned}\tag{333}$$

where the last step is a direct consequence of Lemma 4.7, and holds with probability at least $1-2\exp\left(-c_4\log^2 n\right)$ over X. For the other argument in the max in Eq. (332), we similarly have

$$\begin{aligned}\left\|\mathbb{E}\left[W_k^\top W_k\right]\right\|_{\rm op}&\leq\left\|\mathbb{E}_{v_k}\left[v_kE_k\tilde\phi(Xv_k)^\top\tilde\phi(Xv_k)E_kv_k^\top\right]\right\|_{\rm op}\\&\leq\left\|\mathbb{E}_{v_k}\left[v_kv_k^\top\right]\right\|_{\rm op}\sup_{v_k}\left(E_k\left\|\tilde\phi(Xv_k)\right\|_2^2\right)\\&\leq\frac{1}{d}C_1^2n\\&=\mathcal{O}\left(\frac{n}{d}\right).\end{aligned}\tag{334}$$

Then, plugging these last two equations in Eq. (331) we get

$$\begin{aligned}\mathbb{P}_V\left(\left\|\tilde\Phi EV-\mathbb{E}_V\left[\tilde\Phi EV\right]\right\|_{\rm op}\geq\sqrt{\frac{pn}{d}}\log n\right)&\leq(n+d)\exp\left(-\frac{(pn/d)\log^2 n/2}{C_2(pn/d)+2C_1^2\sqrt{np}\sqrt{n/d}\log n/3}\right)\\&\leq 2\exp\left(-c_5\log^2 n\right),\end{aligned}\tag{335}$$

where we used Assumption 3. Then, applying the triangle inequality and using Eq. (328) and Eq. (335), we get

$$\left\|\tilde\Phi EV\right\|_{\rm op}=\mathcal{O}\left(\sqrt{\frac{pn}{d}}\log n\right),\tag{336}$$

with probability at least $1-2\exp\left(-c_6\log^2 n\right)$ over X,V. To conclude, since $E=I$ with probability at least $1-2p\exp\left(-c_2d\right)$, using Assumption 3 gives the desired result. □

Lemma 4.14. *We have that*

$$\left\|V^\top P_\Lambda^\perp\Phi^+\right\|_{\rm op}=\mathcal{O}\left(\frac{\sqrt{d}}{n}+\frac{\sqrt{n}\log^3 d}{d^{3/2}}\right),\tag{337}$$

with probability at least $1-2\exp\left(-c\log^2 n\right)$ over X and V, where c is an absolute constant.

Proof. Let $\tilde\mu^2:=\sum_{l=3}^\infty\mu_l^2$, where μ_l denotes the l-th Hermite coefficient of ϕ, and let $E\in\mathbb{R}^{n\times n}$ be the matrix defined as

$$E=K-p\left(\mu_1^2\frac{XX^\top}{d}+\tilde\mu^2 I\right).\tag{338}$$

Note that

$$\begin{aligned}\left\|EK^{-1}\right\|_{\rm op}&\leq\left\|K-\mathbb{E}_V\left[K\right]\right\|_{\rm op}\left\|K^{-1}\right\|_{\rm op}+\left\|\mathbb{E}_V\left[K\right]-p\left(\mu_1^2\frac{XX^\top}{d}+\tilde\mu^2 I\right)\right\|_{\rm op}\left\|K^{-1}\right\|_{\rm op}\\&=\left\|\mathbb{E}_V\left[K\right]^{-1/2}\left(K-\mathbb{E}_V\left[K\right]\right)\mathbb{E}_V\left[K\right]^{-1/2}\right\|_{\rm op}\left\|\mathbb{E}_V\left[K\right]\right\|_{\rm op}\left\|K^{-1}\right\|_{\rm op}+\mathcal{O}\left(p\frac{n\log^3 n}{d^{3/2}}\frac{1}{p}\right)\\&=\mathcal{O}\left(\sqrt{\frac{n}{p}}\log n\log p\,\frac{np}{d}\frac{1}{p}\right)+\mathcal{O}\left(\frac{n\log^3 n}{d^{3/2}}\right)\\&=\mathcal{O}\left(\frac{\sqrt{n}}{d}\log^2 n+\frac{n\log^3 n}{d^{3/2}}\right)\\&=\mathcal{O}\left(\frac{n\log^3 n}{d^{3/2}}\right),\end{aligned}\tag{339}$$

where in the second line we used Eq. (223) and $\lambda_{\min}(K) = \Omega(p)$, which holds by Lemma 4.5; in the third line we used Lemma 4.4, $\lambda_{\min}(K) = \Omega(p)$ and $\|\mathbb{E}_V[K]\|_{\text{op}} = \mathcal{O}(np/d)$, which follows from Weyl inequality applied to Eq. (218) and Eq. (224) (conditioning on $\|X\|_{\text{op}} = \mathcal{O}(\sqrt{n})$, given by Lemma 4.2); in the fourth line we used $p = \Omega(n^2)$, and the last step holds due to Assumption 3. The full equation as a whole holds with probability at least $1 - 2\exp(-c_1 \log^2 n)$ over X and V.

By the Woodbury matrix identity (or Hua's identity), we have

$$\begin{aligned} K^{-1} &= \left(p\left(\mu_1^2 \frac{XX^\top}{d} + \tilde{\mu}^2 I \right) + E \right)^{-1} \\ &= \left(\mu_1^2 p \frac{XX^\top}{d} + \tilde{\mu}^2 pI \right)^{-1} - \left(\mu_1^2 p \frac{XX^\top}{d} + \tilde{\mu}^2 pI \right)^{-1} EK^{-1}. \end{aligned} \tag{340}$$

Then, since we have

$$\left\| \frac{pX^\top}{d} \left(\mu_1^2 p \frac{XX^\top}{d} + \tilde{\mu}^2 pI \right)^{-1} \right\|_{\text{op}} \leq \frac{1}{\mu_1^2 \sqrt{\lambda_{\min}(X^\top X)}} = \mathcal{O}\left(\frac{1}{\sqrt{n}} \right), \tag{341}$$

with probability at least $1 - 2\exp(-c_2 \log^2 n)$ over X due to Lemma 4.2, Eq. (340) allows us to write

$$\begin{aligned} \left\| \frac{pX^\top}{d} \left(K^{-1} - \left(\mu_1^2 p \frac{XX^\top}{d} + \tilde{\mu}^2 pI \right)^{-1} \right) \right\|_{\text{op}} &= \left\| \frac{pX^\top}{d} \left(\mu_1^2 p \frac{XX^\top}{d} + \tilde{\mu}^2 pI \right)^{-1} EK^{-1} \right\|_{\text{op}} \\ &= \mathcal{O}\left(\frac{\sqrt{n}\log^3 n}{d^{3/2}} \right), \end{aligned} \tag{342}$$

where we used Eq. (339) and Eq. (341) in the last step, which then holds with probability at least $1 - 2\exp(-c_3 \log^2 n)$ over X and V. Notice that, with this same probability, the application of the triangle inequality to Eq. (341) and Eq. (342), together with Assumption 3, gives

$$\left\| \frac{pX^\top}{d} K^{-1} \right\|_{\text{op}} = \mathcal{O}\left(\frac{1}{\sqrt{n}} \right). \tag{343}$$

Recalling that $\Phi = \mu_1 XV^\top + \tilde{\Phi}$, another triangle inequality yields

$$\begin{aligned} \left\| V^\top \Phi^\top K^{-1} - \frac{\mu_1 pX^\top}{d} K^{-1} \right\|_{\text{op}} &\leq \left\| V^\top \tilde{\Phi}^\top K^{-1} \right\|_{\text{op}} + \mu_1 \left\| \frac{d}{p} V^\top V - I \right\|_{\text{op}} \left\| \frac{pX^\top}{d} K^{-1} \right\|_{\text{op}} \\ &= \mathcal{O}\left(\sqrt{\frac{pn}{d}} \log n \, \frac{1}{p} + \sqrt{\frac{d}{p}} \frac{1}{\sqrt{n}} \right) \\ &= \mathcal{O}\left(\frac{\log n}{\sqrt{dn}} \right), \end{aligned} \tag{344}$$

where the second line holds with probability at least $1 - 2\exp(-c_4 \log^2 n)$ over X and V because of Lemma 4.13, Theorem 4.6.1 in (31) and Eq. (343), and the last step is a consequence of Assumption 3.

We now define $\varrho_\Lambda \in \mathbb{R}^{n \times n}$ as the projector on the span of the eigenvectors associated with the d largest eigenvalues of K. This implies $P_\Lambda \Phi^\top = \Phi^\top \varrho_\Lambda$, and therefore $P_\Lambda^\perp \Phi^+ = \Phi^\top K^{-1} \varrho_\Lambda^\perp$. Hence, we have

$$\left\| \mu_1 VX^\top \varrho_\Lambda^\perp \right\|_{\text{op}} \leq \left\| \Phi^\top \varrho_\Lambda^\perp \right\|_{\text{op}} + \left\| \tilde{\Phi}^\top \varrho_\Lambda^\perp \right\|_{\text{op}} \leq \sqrt{\lambda_{d+1}(K)} + \left\| \tilde{\Phi} \right\|_{\text{op}} \left\| \varrho_\Lambda^\perp \right\|_{\text{op}} = \mathcal{O}(\sqrt{p}), \tag{345}$$

where the last step is a consequence of Lemma 4.5 and Lemma 4.8, and holds with probability at least $1 - 2\exp(-c_5 \log^2 n)$ over X and V. Then, conditioning on this high probability event and on the event $\lambda_{\min}(V^\top V) = \Omega(p/d)$, which holds with probability at least $1 - 2\exp(-c_6 d)$ over V due to Lemma 4.2, we have

$$\left\| X^\top \varrho_\Lambda^\perp \right\|_{\text{op}} \leq \frac{1}{\mu_1 \sqrt{\lambda_{\min}(V^\top V)}} \left\| \mu_1 VX^\top \varrho_\Lambda^\perp \right\|_{\text{op}} = \mathcal{O}(\sqrt{d}). \tag{346}$$

Thus, with probability at least $1 - 2\exp\left(-c_7 \log^2 n\right)$ over X and V, we have

$$
\begin{aligned}
\left\|V^\top P_\Lambda^\perp \Phi^+\right\|_{\text{op}} &\le \left\|\frac{\mu_1 p X^\top}{d} K^{-1} \varrho_\Lambda^\perp\right\|_{\text{op}} + \left\|V^\top \Phi^\top K^{-1} - \frac{\mu_1 p X^\top}{d} K^{-1}\right\|_{\text{op}} \left\|\varrho_\Lambda^\perp\right\|_{\text{op}} \\
&\le \left\|\frac{\mu_1 p X^\top}{d}\left(\mu_1^2 p \frac{XX^\top}{d} + \tilde{\mu}^2 p I\right)^{-1} \varrho_\Lambda^\perp\right\|_{\text{op}} \\
&\quad + \left\|\frac{\mu_1 p X^\top}{d}\left(K^{-1} - \left(\mu_1^2 p \frac{XX^\top}{d} + \tilde{\mu}^2 p I\right)^{-1}\right)\right\|_{\text{op}} \left\|\varrho_\Lambda^\perp\right\|_{\text{op}} + \mathcal{O}\left(\frac{\log n}{\sqrt{dn}}\right) \\
&= \left\|\left(\mu_1 X^\top X + \tilde{\mu}^2 d I/\mu_1\right)^{-1} X^\top \varrho_\Lambda^\perp\right\|_{\text{op}} + \mathcal{O}\left(\frac{\sqrt{n}\log^3 n}{d^{3/2}}\right) + \mathcal{O}\left(\frac{\log n}{\sqrt{dn}}\right) \\
&\le \left\|\left(\mu_1 X^\top X + \tilde{\mu}^2 d I/\mu_1\right)^{-1}\right\|_{\text{op}} \left\|X^\top \varrho_\Lambda^\perp\right\|_{\text{op}} + \mathcal{O}\left(\frac{\sqrt{n}\log^3 n}{d^{3/2}}\right) \\
&= \mathcal{O}\left(\frac{1}{n}\sqrt{d}\right) + \mathcal{O}\left(\frac{\sqrt{n}\log^3 n}{d^{3/2}}\right) \\
&= \mathcal{O}\left(\frac{\sqrt{d}}{n} + \frac{\sqrt{n}\log^3 n}{d^{3/2}}\right),
\end{aligned}
\tag{347}
$$

where the second step holds due to Eq. (344), the third step due to Eq. (342), the fourth due to Assumption 3, and the fifth due to Lemma 4.2 and Eq. (346). This, together with Assumption 3, provides the desired result. □

Lemma 4.15. *Let Assumptions 1 and 2 hold, and let $p = \omega(d)$ and $\log p = \Theta(\log n) = \Theta(\log d)$. Let $x \sim \mathcal{P}_X$. Then, we have*

$$
\left\|\mathbb{E}_x\left[\tilde{\varphi}(x)\tilde{\varphi}(x)^\top\right]\right\|_{\text{op}} = \mathcal{O}\left(\log^4 n + \frac{p\log^3 d}{d^{3/2}}\right), \tag{348}
$$

with probability at least $1 - 2\exp\left(-c\log^2 n\right)$ over V, where c is an absolute constant.

Proof. Let N be a positive natural number that will be defined later, and let $\tilde{\Phi}_N \in \mathbb{R}^{N \times p}$ be a matrix containing $\tilde{\varphi}(\hat{x}_i)$ in its i-th row, where every $\{\hat{x}_i\}_{i=1}^N$ is sampled independently from $\mathcal{P}_X$. Importantly, $\hat{x}_i$ is different from x_i (as they are defined as auxiliary random variables only useful for the purposes of this proof), but $\tilde{\varphi}(\hat{x}_i) = \tilde{\phi}(V\hat{x}_i)$ is defined with the same random features V in Eq. (2). Set

$$
n' = \min\left(\left\lfloor\frac{p}{\log^4 p}\right\rfloor, \left\lfloor\frac{d^{3/2}}{\log^3 d}\right\rfloor\right), \qquad N = p^2 n', \tag{349}
$$

where n' is a positive integer (as p is large enough). Note that this definition guarantees that the triple (n', d, p) satisfies the scalings

$$
n' = \mathcal{O}\left(\frac{p}{\log^4 p}\right), \qquad n'\log^3 n' = \mathcal{O}\left(d^{3/2}\right), \tag{350}
$$

which provide the sufficient hypotheses to apply Lemma 4.8 on a $n' \times p$ block of $\tilde{\Phi}_N$.

Then, $\tilde{\Phi}_N$ can be seen as the vertical stacking of p^2 matrices with size $n' \times p$. All these matrices are independent copies of each other (in the probability space of the $\hat{x}_i$-s), and each of these has operator norm $\mathcal{O}\left(\sqrt{p}\right)$, by Lemma 4.8, with probability at least $1 - 2\exp\left(-c_1\log^2 n'\right)$. Thus, performing a union bound over these p^2 matrices, we get

$$
\left\|\tilde{\Phi}_N^\top \tilde{\Phi}_N\right\|_{\text{op}} = \mathcal{O}\left(p^2 p\right) = \mathcal{O}\left(\frac{Np}{n'}\right) = \mathcal{O}\left(N\log^4 p + \frac{Np\log^3 d}{d^{3/2}}\right), \tag{351}
$$

with probability at least

$$
1 - 2p^2\exp\left(-c_1\log^2 n'\right) \ge 1 - 2p^2\exp\left(-c_2\min\left(\log^2 p, \log^2 d\right)\right) \tag{352}
$$

over V and $\{\hat{x}_i\}_{i=1}^N$. Note that the rows of $\tilde{\Phi}_N$ are identically distributed and such that

$$
\sup_{\hat{x}_i}\left\|\tilde{\varphi}(\hat{x}_i)\right\|_2 \le \left\|\tilde{\phi}(\mathbf{0})\right\|_2 + \tilde{L}\sup_{\hat{x}_i}\left\|V\hat{x}_i\right\|_2 \le \left\|\tilde{\phi}(\mathbf{0})\right\|_2 + \tilde{L}\left\|V\right\|_{\text{op}}\sup_{\hat{x}_i}\left\|\hat{x}_i\right\|_2 = \mathcal{O}\left(\sqrt{p}\right), \tag{353}
$$

where we denote with $\mathbf{0} \in \mathbb{R}^p$ a vector of zeros, $\tilde{L}$ is the Lipschitz constant of $\tilde{\phi}$, and the last step holds due to the bound on $\|V\|_{\text{op}}$ given by Lemma 4.2, which holds with probability at least $1 - 2\exp\left(-c_3 d\right)$ over V (high probability event over which we will condition until the end of the proof). This readily gives, for some constant C_1,

$$
\left\|\tilde{\varphi}(\hat{x}_i)\right\|_{\psi_2} \le C_1\sup_{\hat{x}_i}\left\|\tilde{\varphi}(\hat{x}_i)\right\|_2 = \mathcal{O}\left(\sqrt{p}\right). \tag{354}
$$

We remark that the sub-Gaussian norm in Eq. (354) is intended in the probability space of $\hat{x}_i$, and that it holds jointly for every $i \in [N]$. Then, there exists a sufficiently small absolute constant C_2 such that $C_2 \tilde{\Phi}_N / \sqrt{p}$ is a matrix with independent sub-Gaussian rows, with unit sub-Gaussian norm. Then, by Theorem 5.39 in (36) (see their Remark 5.40, and Equation 5.25), we have that

$$\frac{C_2^2}{p} \left\| \frac{\tilde{\Phi}_N^\top \tilde{\Phi}_N}{N} - \mathbb{E}_x \left[\tilde{\varphi}(x) \tilde{\varphi}(x)^\top \right] \right\|_{\text{op}} = \mathcal{O}\left(\sqrt{\frac{p}{N}} \right), \tag{355}$$

with probability at least $1 - 2\exp(-c_4 p)$ over $\{\hat{x}_i\}_{i=1}^N$.

Then, we have

$$\begin{aligned} \left\| \mathbb{E}_x \left[\tilde{\varphi}(x) \tilde{\varphi}(x)^\top \right] \right\|_{\text{op}} &\le \left\| \frac{\tilde{\Phi}_N^\top \tilde{\Phi}_N}{N} - \mathbb{E}_x \left[\tilde{\varphi}(x) \tilde{\varphi}(x)^\top \right] \right\|_{\text{op}} + \frac{\left\| \tilde{\Phi}_N^\top \tilde{\Phi}_N \right\|_{\text{op}}}{N} \\ &= \mathcal{O}\left(p \sqrt{\frac{p}{N}} \right) + \mathcal{O}\left(\log^4 p + \frac{p \log^3 d}{d^{3/2}} \right) \\ &= \mathcal{O}\left(\sqrt{p} \sqrt{\frac{\log^4 p}{p}} + \sqrt{p} \sqrt{\frac{\log^3 d}{d^{3/2}}} \right) + \mathcal{O}\left(\log^4 p + \frac{p \log^3 d}{d^{3/2}} \right) \\ &= \mathcal{O}\left(\log^4 p + \frac{p \log^3 d}{d^{3/2}} \right), \end{aligned} \tag{356}$$

where the first step follows from the triangle inequality, the second step is a consequence of Eq. (355) and Eq. (351), and the third step is a consequence of Eq. (349).

Taking the intersection between the high probability events in Eq. (351) and Eq. (355), we have

$$\left\| \mathbb{E}_x \left[\tilde{\varphi}(x) \tilde{\varphi}(x)^\top \right] \right\|_{\text{op}} = \mathcal{O}\left(\log^4 n + \frac{p \log^3 d}{d^{3/2}} \right), \tag{357}$$

with probability at least $1 - 2p^2 \exp\left(-c_5 \min\left(\log^2 p, \log^2 d\right)\right) \ge 1 - 2\exp\left(-c_6 \log^2 n\right)$ over $\{\hat{x}_i\}_{i=1}^N$ and V.

Notice that, however, the LHS of the previous equation does not depend on $\{\hat{x}_i\}_{i=1}^N$, which were introduced as auxiliary random variables. Thus, the high probability bound holds restricted to the probability space of V, and the desired result follows. □

Lemma 4.16. *Let Assumptions 1 and 2 hold, and let $n = o\left(p/\log^4 p\right)$, $n \log^3 n = o\left(d^{3/2}\right)$, $n = \omega(d)$ and $\log n = \Theta(\log p)$. Then, we have*

$$\mathbb{E}_x \left[\left(\varphi(x)^\top \theta^* \right)^2 \right] = \mathcal{O}(1), \tag{358}$$

with probability at least $1 - 2\exp\left(-c \log^2 n\right)$ over X and V, where c is an absolute constant.

Proof. First, we can upper bound the LHS of the thesis as

$$\mathbb{E}_x \left[\left(\varphi(x)^\top \theta^* \right)^2 \right] \le 2\mu_1^2 \mathbb{E}_x \left[\left(x^\top V^\top \theta^* \right)^2 \right] + 2\mathbb{E}_x \left[\left(\tilde{\varphi}(x)^\top \theta^* \right)^2 \right]. \tag{359}$$

We bound the two terms separately. Since x is distributed according to $\mathcal{P}_X$, it is sub-Gaussian with $\|x\|_{\psi_2} = \mathcal{O}(1)$. Then, we can bound its second moment (see (31), Proposition 2.5.2) as follows

$$\mathbb{E}_x \left[\left(x^\top V^\top \theta^* \right)^2 \right] \le C_1 \left\| V^\top \theta^* \right\|_2^2 \le C_1 \left\| V^\top \Phi^+ \right\|_{\text{op}}^2 \|Y\|_2^2 = \mathcal{O}\left(\frac{1}{n} n \right) = \mathcal{O}(1), \tag{360}$$

where C_1 represents an absolute constant, and the third step holds with probability at least $1 - 2\exp\left(-c_1 \log^2 n\right)$ over X and V because of Lemma 4.10.

Next, we bound the second term of Eq. (359) as

$$\begin{aligned} \mathbb{E}_x \left[\left(\tilde{\varphi}(x)^\top \theta^* \right)^2 \right] &= (\theta^*)^\top \mathbb{E}_x \left[\tilde{\varphi}(x) \tilde{\varphi}(x)^\top \right] \theta^* \\ &\le \left\| \mathbb{E}_x \left[\tilde{\varphi}(x) \tilde{\varphi}(x)^\top \right] \right\|_{\text{op}} \|\theta^*\|_2^2 \\ &\le \left\| \mathbb{E}_x \left[\tilde{\varphi}(x) \tilde{\varphi}(x)^\top \right] \right\|_{\text{op}} \left\| \Phi^+ \right\|_{\text{op}}^2 \|Y\|_2^2 \\ &= \mathcal{O}\left(\left(\log^4 n + \frac{p \log^3 d}{d^{3/2}} \right) \frac{1}{p} n \right) \\ &= \mathcal{O}\left(\frac{n \log^4 n}{p} + \frac{n \log^3 d}{d^{3/2}} \right) = o(1), \end{aligned} \tag{361}$$

where the fourth line holds because of Lemmas 4.15 and 4.5 with probability at least $1 - 2\exp\left(-c_2 \log^2 n\right)$ over X and V. The last line provides the desired result. □

References

1. K Chaudhuri, C Monteleoni, Privacy-preserving logistic regression in *Advances in Neural Information Processing Systems.* (2008).
2. K Chaudhuri, C Monteleoni, AD Sarwate, Differentially private empirical risk minimization. *J. Mach. Learn. Res.* **12**, 1069–1109 (2011).
3. D Kifer, A Smith, A Thakurta, Private convex empirical risk minimization and high-dimensional regression in *Conference on Learning Theory.* (2012).
4. R Bassily, A Smith, A Thakurta, Private empirical risk minimization: Efficient algorithms and tight error bounds in *2014 IEEE 55th Annual Symposium on Foundations of Computer Science.* (2014).
5. O Williams, F Mcsherry, Probabilistic inference and differential privacy in *Advances in Neural Information Processing Systems.* (2010).
6. S Shalev-Shwartz, N Srebro, K Sridharan, Stochastic convex optimization in *Conference on Learning Theory.* (2009).
7. M Hardt, B Recht, Y Singer, Train faster, generalize better: Stability of stochastic gradient descent in *International Conference on Machine Learning.* (2016).
8. R Bassily, V Feldman, K Talwar, A Guha Thakurta, Private stochastic convex optimization with optimal rates in *Advances in Neural Information Processing Systems.* (2019).
9. TT Cai, Y Wang, L Zhang, The cost of privacy: Optimal rates of convergence for parameter estimation with differential privacy. *The Annals Stat.* **49**, 2825 – 2850 (2021).
10. K Chaudhuri, PL Loh, S Pandey, P Sarkar, On differentially private u statistics. *arXiv preprint arXiv:2407.04945* (2024).
11. M Avella-Medina, C Bradshaw, PL Loh, Differentially private inference via noisy optimization. *The Annals Stat.* **51**, 2067 – 2092 (2023).
12. P Jain, AG Thakurta, (near) dimension independent risk bounds for differentially private learning in *International Conference on Machine Learning.* (2014).
13. S Song, T Steinke, O Thakkar, A Thakurta, Evading the curse of dimensionality in unconstrained private glms in *International Conference on Artificial Intelligence and Statistics.* (2021).
14. X Li, et al., When does differentially private learning not suffer in high dimensions? in *Advances in Neural Information Processing Systems.* (2022).
15. YA Ma, TV Marinov, T Zhang, Dimension independent generalization of dp-sgd for overparameterized smooth convex optimization. *arXiv preprint arXiv:2206.01836* (2022).
16. R Arora, R Bassily, CA Guzmán, M Menart, E Ullah, Differentially private generalized linear models revisited in *Advances in Neural Information Processing Systems.* (2022).
17. P Varshney, A Thakurta, P Jain, (nearly) optimal private linear regression for sub-gaussian data via adaptive clipping in *Conference on Learning Theory.* (2022).
18. G Andrew, O Thakkar, HB McMahan, S Ramaswamy, Differentially private learning with adaptive clipping in *Advances in Neural Information Processing Systems.* (2021).
19. YX Wang, Revisiting differentially private linear regression: optimal and adaptive prediction & estimation in unbounded domain. *arXiv preprint arXiv:1803.02596* (2018).
20. J Milionis, A Kalavasis, D Fotakis, S Ioannidis, Differentially private regression with unbounded covariates in *International Conference on Artificial Intelligence and Statistics.* (2022).
21. X Liu, W Kong, S Oh, Differential privacy and robust statistics in high dimensions in *Conference on Learning Theory.* (2022).
22. X Liu, P Jain, W Kong, S Oh, AS Suggala, Near optimal private and robust linear regression. *arXiv preprint arXiv:2301.13273* (2023).
23. GR Brown, et al., Private gradient descent for linear regression: Tighter error bounds and instance-specific uncertainty estimation in *Forty-first International Conference on Machine Learning.* (2024).
24. S Bombari, S Kiyani, M Mondelli, Beyond the universal law of robustness: Sharper laws for random features and neural tangent kernels in *International Conference on Machine Learning.* (2023).
25. C Dwork, A Roth, The algorithmic foundations of differential privacy. *Foundations Trends Theor. Comput. Sci.* **9**, 211–407 (2014).
26. M Abadi, et al., Deep learning with differential privacy in *ACM SIGSAC Conference on Computer and Communications Security.* (2016).
27. P Kloeden, E Platen, *Numerical Solution of Stochastic Differential Equations*, Stochastic Modelling and Applied Probability. (Springer Berlin Heidelberg), (2011).
28. S Menozzi, A Pesce, X Zhang, Density and gradient estimates for non degenerate brownian sdes with unbounded measurable drift. *J. Differ. Equations* **272**, 330–369 (2021).
29. C Gardiner, *Handbook of Stochastic Methods for Physics, Chemistry, and the Natural Sciences*, Proceedings in Life Sciences. (Springer-Verlag), (1985).
30. S Bombari, M Mondelli, How spurious features are memorized: Precise analysis for random and ntk features in *International Conference on Machine Learning.* (2024).
31. R Vershynin, *High-dimensional probability: An introduction with applications in data science.* (Cambridge university press), (2018).

32. S Mei, T Misiakiewicz, A Montanari, Generalization error of random feature and kernel methods: Hypercontractivity and kernel matrix concentration. *Appl. Comput. Harmon. Analysis* **59**, 3–84 (2022).
33. R van Handel, Probability in high dimensions (2016).
34. D Revuz, M Yor, *Continuous Martingales and Brownian Motion*, Grundlehren der mathematischen Wissenschaften. (Springer Berlin, Heidelberg), (2010).
35. RJ Adler, JE Taylor, *Random Fields and Geometry*, Springer Monographs in Mathematics. (Springer New York, NY) Vol. 1, (2007).
36. R Vershynin, *Introduction to the non-asymptotic analysis of random matrices.* (Cambridge University Press), p. 210–268 (2012).
37. S Bombari, MH Amani, M Mondelli, Memorization and optimization in deep neural networks with minimum over-parameterization in *Advances in Neural Information Processing Systems.* (2022).
